# DDM1-mediated gene body DNA methylation is associated with inducible activation of defense-related genes in *Arabidopsis*

**DOI:** 10.1186/s13059-023-02952-7

**Published:** 2023-05-05

**Authors:** Seungchul Lee, Jaemyung Choi, Jihwan Park, Chang Pyo Hong, Daeseok Choi, Soeun Han, Kyuha Choi, Tae-Young Roh, Daehee Hwang, Ildoo Hwang

**Affiliations:** 1 Department of Life Sciences, POSTECH, Pohang, 37673 Korea; 2 Department of Cell & Developmental Biology, John Innes Centre, Norwich, NR4 7UH UK; 3 School of Interdisciplinary Bioscience and Bioengineering, POSTECH, Pohang, 37673 Korea; 4 Department of Biological Sciences, Seoul National University, Seoul, 08826 Korea

**Keywords:** Gene body methylation, Chromatin remodeler DDM1, Natural epigenetic variation, Priming

## Abstract

**Background:**

Plants memorize previous pathogen attacks and are “primed” to produce a faster and stronger defense response, which is critical for defense against pathogens. In plants, cytosines in transposons and gene bodies are reported to be frequently methylated. Demethylation of transposons can affect disease resistance by regulating the transcription of nearby genes during defense response, but the role of gene body methylation (GBM) in defense responses remains unclear.

**Results:**

Here, we find that loss of the chromatin remodeler decrease in DNA methylation 1 (*ddm1*) synergistically enhances resistance to a biotrophic pathogen under mild chemical priming. DDM1 mediates gene body methylation at a subset of stress-responsive genes with distinct chromatin properties from conventional gene body methylated genes. Decreased gene body methylation in loss of *ddm1* mutant is associated with hyperactivation of these gene body methylated genes. Knockout of glyoxysomal protein kinase 1 (*gpk1*), a hypomethylated gene in *ddm1* loss-of-function mutant, impairs priming of defense response to pathogen infection in *Arabidopsis*. We also find that DDM1-mediated gene body methylation is prone to epigenetic variation among natural *Arabidopsis* populations, and *GPK1* expression is hyperactivated in natural variants with demethylated *GPK1*.

**Conclusions:**

Based on our collective results, we propose that DDM1-mediated GBM provides a possible regulatory axis for plants to modulate the inducibility of the immune response.

**Supplementary Information:**

The online version contains supplementary material available at 10.1186/s13059-023-02952-7.

## Background

DNA methylation protects the genome from the randomized insertion of transposons (TEs) [1–4]. Propagation or jumping of TEs requires transcription of TE genes, but DNA methylation silences TE genes to prevent transcription. DNA methylation in plants occurs in three cytosine contexts: CG, CHG, and CHH (H represents A, C, or T). Three families of DNA methyltransferases methylate different cytosine contexts [5, 6]. During DNA replication, the DNA methyltransferase DNMT1 is recruited to hemimethylated CG and restores fully methylated status in mammalian cells [7, 8]. MET1 is a DNMT1 homolog in *Arabidopsis thaliana* that maintains CG methylation. Chromomethyltransferases (CMT2 and CMT3) are plant-specific DNA methyltransferases that recognize K9-methylated histone H3 (H3K9me) and then methylate CHH (CMT2) and CHG (CMT3) contexts [9–12]. DRM1 and DRM2 are DNMT3 homologs that methylate the CHH context [6, 13].

In *Arabidopsis*, CMTs and DRMs methylate heterochromatic TEs (hTEs) and euchromatic TEs (eTEs), respectively, in a mutually exclusive manner [10, 14]. hTEs are usually long and often possess TE genes. eTEs primarily consist of short, non-autonomous TEs. hTEs are concentrated at pericentromeric regions and heavily methylated by MET1 and CMTs. A linker histone H1 usually blocks the access of DNA methyltransferases to hTEs. As a Snf2 family chromatin remodeler, DDM1 can enable DNA methyltransferases to access the H1-rich heterochromatin [10, 15]. In *ddm1* plants, DNA methylation at hTEs is severely compromised in all cytosine contexts. eTEs populate near genes (chromosome arms), which can modulate the expression of nearby genes in *Arabidopsis* [16]. MET1 methylates the CG context of eTEs, which plays a vital role in recruiting DRMs to methylate the CHH context [17]. Therefore, loss of MET1 results in the severe loss of DNA methylation at eTEs. As the chromatin at eTEs is readily accessible to DNA methyltransferases, the chromatin remodeler DDM1 has a limited effect on eTE DNA methylation [10].

DNA methylation is associated with the defense response in plants. Salicylic acid (SA) signaling plays a pivotal role in defense against biotrophic pathogens [18, 19]. DNA hypomethylation results in faster and stronger activation of SA signaling to enhance resistance against biotrophic pathogens [20–22]. In particular, loss of CG and/or CHH methylation, presumably at eTEs on the promoters, is associated with the activation of nearby defense-related genes [23–25]. For instance, lack of DNA demethylase increases DNA methylation at the eTE on the promoter of *RMG1*, a potential upstream regulator of the defense response, suppressing its expression [26, 27]. Suppression of *RMG1* might affect the downstream defense response in *trans*, thereby modulating plant immunity.

In addition to TEs, the bodies of long genes are methylated in plants. Unlike methylation occurring at both CG and nonCG contexts in TEs, gene body methylation (GBM) occurs only in the CG context [28, 29]. In plants, GBM is evolutionarily conserved [30, 31] and prevents accumulation of the histone variant H2A.Z at genes. H2A.Z is associated with high responsiveness and variable expression of genes [32, 33]. Therefore, GBM is enriched at stably expressed housekeeping genes [32, 34, 35]. However, the functional role of GBM in transcriptional regulation remains elusive [6, 36, 37], as GBM is proposed to be a nonfunctional byproduct of methylation in TEs [36, 38].

Fast activation of defense-related transcriptomes is essential to effectively immunize plants against pathogens [39, 40]. For example, pre-treatment with β-aminobutyric acid (BABA) can induce a faster and stronger defense response when plants encounter pathogens. This induced resistance is called “priming” [41–43]. Here, we investigate the roles of CG methylation at TEs and gene bodies in the regulation of priming during the defense response in *Arabidopsis.* We establish that DDM1-mediated GBM suppresses inducible activation of defense-related genes, and the loss of GBM in *ddm1* activates BABA-induced resistance.

## Results

### Loss of CG methylation enables an augmented defense response in weakly primed plants

**Fig. 1:**
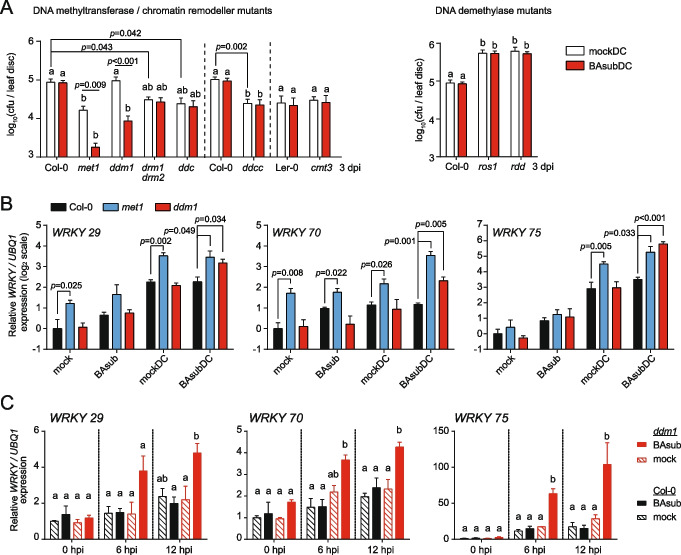
Augmented defense response against the biotrophic pathogen in CG-hypomethylated mutants. **A** Bacterial growth of *Pseudomonas syringae pv. tomato* DC3000 (DC) in DNA methylation mutants with or without sub-optimal priming. For sub-optimal priming, water (mock control) or *β*-amino butyric acid (BAsub; 10 ml of 30 μg/ml per plant) was applied near roots 2 days prior pathogen inoculation. Bacterial growth was measured 3 days post-inoculation (dpi) from at least five biological replicates (*n* ≧ 5). *ddc, ddcc,* and *rdd* indicate *drm1 drm2 cmt3, drm1 drm2 cmt2 cmt3,* and *ros1 dml2 dml3* plants, respectively. *p*-values by Student’s* t* test. cfu, colony-forming units. **B** Expression of *WRKY* genes in Col-0*, ddm1*, and *met1* plants before (0 dpi; mock and BAsub) and after (1 dpi; mockDC and BAsubDC) DC inoculation (*n* ≧ 3). *p*-values by Student’s* t* test. Expression levels for each gene were normalized by that of an internal control *UBQ1*, which were further normalized with respect to those in Col-0 under the mock condition. Log_2_(normalized expression levels) are presented. **C** Expression of *WRKY* genes in Col-0 and *ddm1* plants at early time points after DC inoculation (0, 6, and 12 h post-inoculation, hpi). *UBQ1* expression was used as an internal control (*n* = 3). **A, C** Different letters indicate significant differences at *p*≦0.05, from two-way (A) and one-way (C) analysis of variance (ANOVA) with Tukey’s correction (*α* = 0.05). **A–C** Error bars indicate the standard error of the mean (SEM)

We first confirmed the function of DNA methylation in the defense response by testing the growth of a bacterial pathogen *Pseudomonas syringae pathovar* (*pv.*) *tomato* strain DC3000 (DC) in wild-type plants (Col-0 and Ler-0) and DNA methylation mutants (Fig. 1A). The DNA demethylase mutants had increased DNA methylation and compromised resistance (Fig. 1A, white bar; *ros1* and *ros1 dml2 dml3* (*rdd*) [26, 27, 44]), whereas the *met1* mutation, which decreases CG, significantly enhanced resistance (Fig. 1A, white bar; *met1*). Consistent with a previous report [23], CHH demethylation (*drm1 drm2* plants) and nonCG (CHG and CHH) demethylation (*drm1 drm2 cmt3* plants (*ddc*) and *drm1 drm2 cmt2 cmt3* plants (*ddcc*)) suppressed pathogen growth (Fig. 1A, white bar; *drm1 drm2, ddc*). Specific loss of CHG methylation did not affect resistance (Fig. 1A, white bar; *cmt3*). These results suggest that global CG hypomethylation (*met1*) or CHH hypomethylation (*met1*, *drm1 drm2*, *ddc*, and *ddcc*) enhances resistance to DC, whereas loss of CHG methylation (*cmt3*) has a limited effect.

DDM1 mediates both CG and nonCG methylation, and *ddm1* knockout thus partially compromises DNA methylation in all cytosine contexts [10, 15]. A previous study showed that *ddm1* plants exhibit enhanced resistance to DC [23]. However, we did not observe altered immunity in *ddm1* plants (Fig. 1A, white bar, *ddm1*), suggesting that the partial DNA methylation loss was insufficient to induce disease resistance under our experimental conditions. Thus, we assisted the defense response by applying BABA. High-dose BABA treatment (10 ml of 35 μg/ml; BAopt) for 3 days can prime an immune response in Col-0 plants [41–43] (Additional file 1: Fig. S1A and S1B). However, lowering the dose (10 ml of 30 μg/ml BABA) and shortening the treatment time (2 days), named “sub-optimal” (BAsub), did not enhance resistance in Col-0 plants (Fig. 1A and Additional file 1: Fig. S1C); therefore, whether the DNA methylation mutants suppress bacterial growth can be effectively evaluated in BAsub. BAsub treatment enhanced resistance only in *met1* and *ddm1* plants (Fig. 1A, red bars) and did not affect resistance in *drm1 drm2, cmt3, ddc,* and *ddcc* plants, suggesting that CG hypomethylation and BABA-induced chemical priming can synergistically induce resistance independently of CHG and CHH methylation.

We next examined the expression levels of three *WRKY* defense marker genes (*WRKY29*, *WRKY70*, and *WRKY75*) during defense response [45–47] (Fig. 1B and Additional file 1: Fig. S1D). In a water-treated mock control (mock), *WRKY29* and *WRKY70* expression was already upregulated in *met1* plants compared to Col-0 (Fig. 1B). When we inoculated mock- and BAsub-treated plants with DC (mockDC and BAsubDC), *WRKY* expression was further hyperactivated at 1 day post-inoculation (1 dpi) in *met1* plants (Fig. 1B and Additional file 1: Fig. S1D). By contrast, the expression of *WRKY* genes was not changed in *ddm1* compared to Col-0 after mock, BAsub, and mockDC treatments, but hyperactivated after BAsubDC treatment (Fig. 1B and Additional file 1: Fig. S1D). These data are consistent with the resistance phenotype (Fig. 1A) because *ddm1* plants were more resistant to DC only after being weakly primed with BABA, whereas *met1* plants showed enhanced resistance under the mockDC condition, and BAsub treatment further fortified the immunity of plants. The *WRKY* expression level increased from 6 h post-DC inoculation (6 hpi) in weakly primed *ddm1* plants, whereas Col-0 plants and non-primed *ddm1* plants showed no or subtle upregulation of *WRKY* expression at 6 hpi (Fig. 1C). Therefore, the loss of *ddm1* induced faster and stronger transcription of *WRKY* genes during a weakly primed defense response.

### Effector-triggered immune response genes are hyperactivated in weakly primed *ddm1* plants

**Fig. 2:**
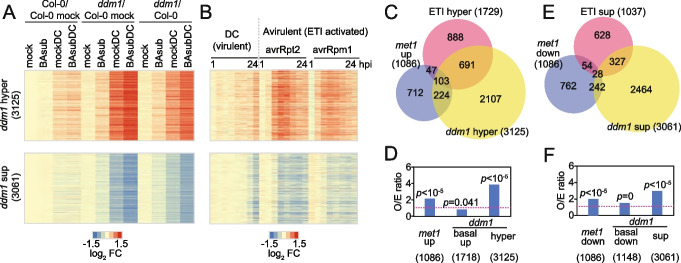
Effector-triggered immune responsive genes were hyperactivated in weakly primed *ddm1* plants. **A** Heatmaps of gene expression in Col-0 and *ddm1* plants before (0 dpi; mock and BAsub) and after (1 dpi; mockDC and BAsubDC) DC inoculation. For Col-0 and *ddm1* columns, log_2_ fold change (FC) against Col-0 mock samples were plotted. For *ddm1*/Col-0 columns, log_2_ fold change in *ddm1* plants against Col-0 under four different conditions were plotted. **B** Heatmaps of gene expression changes after inoculation of avirulent (avrRpt2 and avrRpm1) or virulent (DC) pathogens. Log_2_ fold change (FC) against mock control at each time point was plotted. **C** Venn diagram of hyperactivated genes in *ddm1* (vs. Col-0; *ddm1* hyper), hyperactivated genes after avirulent pathogen inoculation (avrRpt2 or avrRpm1 vs. DC; ETI hyper), and upregulated genes in *met1* (vs. Col-0; *met1* up). **D** Observed to expected ratio of the number of overlaps between ETI hyper genes and other gene groups in **C**. *ddm1* basal up indicates basally upregulated genes in *ddm1* (vs. Col-0) under mock condition. **E** Venn diagram of suppressed genes in *ddm1* (vs. Col-0; *ddm1* sup), suppressed genes after avirulent pathogen inoculation (avrRpt2 or avrRpm1 vs. DC; ETI sup), and downregulated genes in *met1* (vs. Col-0; *met1* down). **F** Observed to expected ratio of the number of overlaps between ETI sup genes and other gene groups in **E**. *ddm1* basal down indicates basally downregulated genes in *ddm1* (vs. Col-0) under mock condition. **D, F** *p*-values by Fisher’s exact test

To gain insight into how DDM1 modulates the defense transcriptome, we measured gene expression under four treatment conditions (mock, BAsub, mockDC, and BAsubDC) in Col-0 and *ddm1* plants using microarray analyses (Fig. 2A and Additional file 1: Fig. S2A and S2B). Among 26,327 genes on the microarray, 11,138 were identified as differentially expressed genes (DEGs) between *ddm1* and Col-0 under at least one treatment condition (Additional file 1: Fig. S2A). We categorized these genes into four groups based on their differential expression patterns under mock and BAsubDC conditions: (1) basally upregulated (*ddm1* basal up; 1,718 genes in Additional file 1: Fig. S2A and S2B) or (2) downregulated genes (*ddm1* basal down; 1148 genes) in *ddm1* plants (vs. Col-0) under the mock condition; and (3) hyperactivated (*ddm1* hyper; 3125 genes in Fig. 2A) or (4) suppressed (*ddm1* sup; 3061 genes) genes in *ddm1* plants (vs. Col-0) under the BAsubDC condition, but not under the mock condition (Figs. 2A and Additional file 1: Fig. S2A). Intriguingly, *ddm1* hyper and sup genes showed no significant changes in *ddm1* (vs. Col-0) under BAsub, but apparent up- or downregulation under mockDC, which was further boosted under BAsubDC, indicating potent priming effects (Fig. 2A). To investigate which group of genes contributed to enhanced resistance in *ddm1*, we analyzed the enriched Gene Ontology (GO) terms and found that *ddm1* hyper genes were enriched with “defense response,” “response to external biotic stimulus,” and “defense response to fungus” while the other groups had no enrichment of defense-related GO terms (Additional file 1: Fig. S2D).

Avirulent pathogens possessing effectors trigger faster and stronger activation of defense-related genes than virulent pathogens, eventually fortifying plant immunity [48–50]. This boosted immune response is called effector-triggered immunity (ETI). To examine whether *ddm1* hyper genes are also activated during ETI, we reanalyzed previously reported datasets [40] generated during ETI. Col-0 plants were inoculated with avirulent (*Pseudomonas syringae pv. tomato* avrRpt2 and avrRpm1) or virulent (DC) pathogens, and gene expression was measured from 1 to 24 hpi (Figs. 2B and Additional file 1: Fig. S2C). We then examined temporal gene expression patterns of *ddm1* hyper genes in these datasets. *ddm1* hyper genes were activated as early as 4 hpi with avrRpt2 and avrRpm1, whereas their expression peaked at 24 hpi with DC (Fig. 2B, top). *ddm1* sup genes were quickly suppressed compared with DC (Fig. 2B, bottom). Both basal up and down genes in *ddm1* were suppressed by inoculation with avirulent pathogens (Additional file 1: Fig. S2C). We next identified hyperactivated (ETI hyper) or suppressed (ETI sup) genes after treatment with avrRpt2 and avrRpm1 (vs*.* DC, differentially expressed at 6 or 9 hpi; *p* ≦ 0.01) and compared them with the above four gene groups in *ddm1*. We found that 46% of ETI hyper genes overlapped with *ddm1* hyper genes, which was 3.86-fold more than the expected portion of random overlap (*p* < 10^–5^, Fig. 2C, D, Additional file 2: Supplementary data 1), and 34% of ETI sup genes overlapped with *ddm1* sup genes (2.94-fold and* p* < 10^–5^, Fig. 2E, F, Additional file 2: Supplementary data 1). In contrast, only 9% of *ddm1* basal up genes overlapped with ETI hyper genes, and 11% of *ddm1* basal down genes overlapped with ETI sup genes (Fig. 2D, F, Additional file 2: Supplementary data 1). The early activation of *ddm1* hyper genes and their significant overlap with ETI hyper genes suggest potential associations of *ddm1* hyper genes with induced immunity in ETI.

Moreover, *met1*, *drm1 drm2*, *ddc*, and *ddcc* plants showed enhanced resistance to DC without BAsub (Fig. 1A). Comparison of the resistance between mutants and their corresponding wild-type plants under mockDC condition revealed that both CG demethylation (*met1*) or CHH demethylation (*met1*, *drm1 drm2*, *ddc*, and *ddcc*) enhanced resistance to DC, whereas CHG demethylation (*cmt3*) has little effect on resistance to DC (Fig. 1A). Consistent with resistance in *met1* plants, up- and downregulated genes in *met1* plants under the mock condition [51] significantly overlapped with ETI hyper and ETI sup genes (Fig. 2C–F). However, comparison of the resistance between mockDC and BAsubDC showed that BAsub treatment boosted resistance to DC only in mutants showing CG demethylation (*met1* and *ddm1*), consistent with significant overlaps of ETI-responsive genes with *ddm1* hyper or sup genes in weakly primed conditions. These results suggest that global loss of CG methylation in *met1* and *ddm1* plants contributed to the enhanced disease resistance with weak BAsub priming, whereas loss of CHH methylation enhanced resistance to DC independent of priming.

### DDM1 mediates both TE-like and gene body-like methylation

To investigate how BAsub treatment boosted immunity in CG-hypomethylated plants, we focused on *ddm1* because *met1* plants also showed enhanced resistance without BAsub. We first produced genome-wide DNA methylation profiles in Col-0 and *ddm1* plants with no treatment of BAsub or DC. We then identified CG or nonCG methylated regions from the profiles in Col-0 by selecting 50-bp windows with significant DNA methylation in CG or nonCG (CHG or CHH) contexts and then merging nearby windows (within 500 bp). The distributions of CG, CHG, and CHH methylation levels in the merged windows are shown in Additional file 1: Fig. S3A. Based on these distributions, the merged windows having more than 10% CG, 5% CHG, and 2% CHH methylation were defined as TE-like methylation (TEM-like) regions while those having more than 10% CG but less than 5% CHG and 2% CHH methylation were as gene body-like methylation (GBM-like) regions. In Col-0 plants, 67% of methylated regions were TEM-like, and 33% were GBM-like ones (Additional file 1: Fig. S3B).

**Fig. 3:**
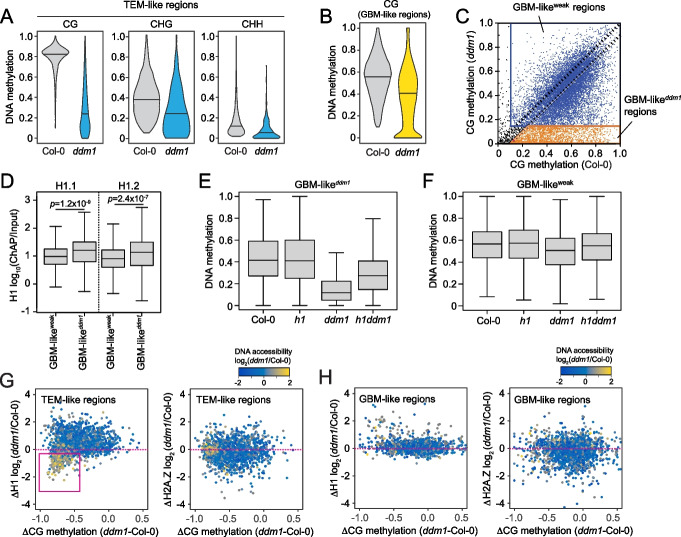
Transposon-like methylation (TEM-like) and gene body-like methylation (GBM-like) patterns in *ddm1* plants. **A** Violin plots of DNA methylation levels of TEM-like regions in Col-0 and *ddm1* plants. **B** Violin plots of CG methylation levels of GBM-like regions in Col-0 and *ddm1* plants. **C** Scatter plot of CG methylation level of GBM-like regions in Col-0 (*x*-axis) and *ddm1* (*y*-axis) plants. Each dot represents a gene body methylation locus. Orange dots indicates GBM-like^*ddm1*^, and blue dots indicate GBM-like^weak^. GBM-like^*ddm1*^; strongly demethylated GBM-like regions in *ddm1*. GBM-like^weak^; GBM-like regions that their methylation levels are weakly affected or not affected in *ddm1*. **D** Histone H1.1 and H1.2 level at GBM-like^*ddm1*^ and GBM-like^weak^. *p*-values from Student’s *t* test. **E, F** Gene body CG DNA methylation level at GBM-like^*ddm1*^ (**E**) and GBM-like.^weak^ (**F**) in Col-0, *h1*, *ddm1*, and *h1ddm1* plants. **G** Scatter plots of CG methylation change at TEM-like regions in relation to histone H1 and H2A.Z change in *ddm1*. Magenta box indicates highly demethylated TEM-like regions with increased DNA accessibility in *ddm1*. **H** Scatter plots of CG methylation change at GBM-like regions in relation to histone H1 and H2A.Z change in *ddm1*. **G, H** Dot color shows log_2_ ratio of DNA accessibility in Col-0 to *ddm1*

As expected, DNA methylation was severely disrupted at TEM-like sites in *ddm1* plants, indicating the crucial role of DDM1 in maintaining DNA methylation at TEs (Fig. 3A) [10, 15]. For integrated analysis of TEM-like in *ddm1*, *met1*, and *drm2*, we reanalyzed previous bisulfite datasets [10, 52] generated from *met1* and *drm2* plants to identify TEM-like in these mutants. When we isolated DDM1- and MET1-mediated TEM-like that simultaneously lost CG, CHG, and CHH methylation in *ddm1* and *met1* plants (Additional file 1: Fig. S3C), demethylated TEM-like in *ddm1* (TEM-like^*ddm1*^) had high levels of H3K9me2 and histone H1 in Col-0. In contrast, demethylated TEM-like in *met1* (TEM-like^*met1*^) had low levels of H3K9me2 and H1 (Additional file 1: Fig. S3D) [10, 53], significantly overlapping with CHH demethylated TEM-like in *drm2* (TEM-like^*drm2*^) (Additional file 1: Fig. S3C and S3E).

On the other hand, consistent with a previous finding [15], DDM1 had a limited effect on GBM-like regions (Fig. 3B). The distribution of CG methylation levels in Col-0 and *ddm1* showed two groups of GBM-like regions (Figs. 3C and Additional file 1: Fig. S3F, Additional file 2: Supplementary data 1): (1) GBM-like^*ddm1*^ group showing significantly lost CG methylation in *ddm1* (Fig. 3C, orange dots in bottom box) and (2) GBM-like^weak^ group showing relatively weak or no CG methylation changes (Fig. 3C, blue dots in top box). As *ddm1* plants can exhibit random fluctuations of DNA methylation, we tested whether GBM-like demethylation in *ddm1* occurred randomly. CG methylation changes in these two groups were consistent to those observed in other independent data sets (Additional file 1: Fig. S3G for GBM-like^*ddm1*^ and Additional file 1: Fig. S3H for GBM-like^weak^). Previous studies suggested that DDM1 enables the access of DNA methylases to the H1-rich DNA. Hence, *ddm1* plants lose DNA methylation in H1-rich regions, and the lost methylation is rescued by knockdown of *h1* [10, 15]. Consistent with this, GBM-like^*ddm1*^ regions had higher H1 levels than GBM-like^weak^ regions (Fig. 3D), and regained DNA methylation in *h1ddm1* (Fig. 3E) [51]. In contrast, DNA methylation at GBM-like^weak^ regions showed subtle changes in *ddm1* or *h1ddm1* plants (Fig. 3F) [10, 15]. These results imply that DDM1 mediates GBM-like at specific loci, e.g., H1-rich regions.

DDM1, a chromatin remodeler, affects DNA accessibility, DNA methylation, H3K9me2 levels, and H2A.W deposition at heterochromatin [10, 51, 54], and perturbation of DNA methylation has been suggested to indirectly modulate H1 and H2A.Z distribution in *Arabidopsis* [33, 55]. Therefore, we next investigated whether changes of DNA methylation in *ddm1* correlated with those of H1, H2A.Z, H2A.W, and H3K9me2. To this end, we performed chromatin immunoprecipitation (ChIP) sequencing of H1 and H2A.Z in Col-0 and *ddm1* plants and used previously reported ChIP sequencing data for H2A.W and H3K9me2 [54]. For the TEM-like, changes of CG methylation levels between *ddm1* and Col-0 showed positive correlations for highly demethylated TEM-like in *ddm1* (more than 0.5 loss of CG methylation in *ddm1*) with those of H1, H2A.W, and H3K9me2 (Figs. 3G and Additional file 1: Fig. S3I, magenta box). On the other hand, there was no significant correlation for H2A.Z (Fig. 3G). However, when the same analysis was done using a previously reported H2A.Z profile generated from Col-0 and *met1* [33, 52], CG methylation changes in *met1* (vs. Col-0) showed a stronger correlation for highly demethylated TEM-like (Additional file 1: Fig. S3K, magenta box) compared to in *ddm1*, potentially due to suppression of H2A.Z accumulation by the remaining CG methylation after the limited demethylation in *ddm1*, unlike extensive demethylation in *met1* (compare CG methylation levels of *ddm1* (Fig. 3A) and *met1* (Additional file 1: Fig. S3L)). These data suggest that demethylation at TEM-like in *ddm1* is independent to H2A.Z changes and partially correlated with changes of H1, H2A.W, and H3K9me2 for highly demethylated TEM-like. Moreover, we also examined the association of CG methylation changes with those of H1, H2A.Z, H2A.W, and H3K9me2 in all GBM-like regions. Unlike TEM-like, CG methylation changes in GBM-like regions showed no correlation with those of and H1, H2A.Z, H2A.W, and H3K9me2 (Figs. 3H and Additional file 1: Fig. S3J). Differently from the negative correlation between CG methylation and H2A.Z changes for highly demethylated TEM-like, in case of GBM-like, there was no significant correlation in *met1* regardless of demethylation extents (Additional file 1: Fig. S3K). Therefore, at GBM-like regions, *ddm1* knockout specifically regulated DNA methylation, but independently with histone composition changes, unlike the diverse correlation patterns of *ddm1* knockout with histone composition changes at TEM-like.

### DDM1-mediated TE-like methylation at gene bodies and transcription start sites potentiates transcription

**Fig. 4:**
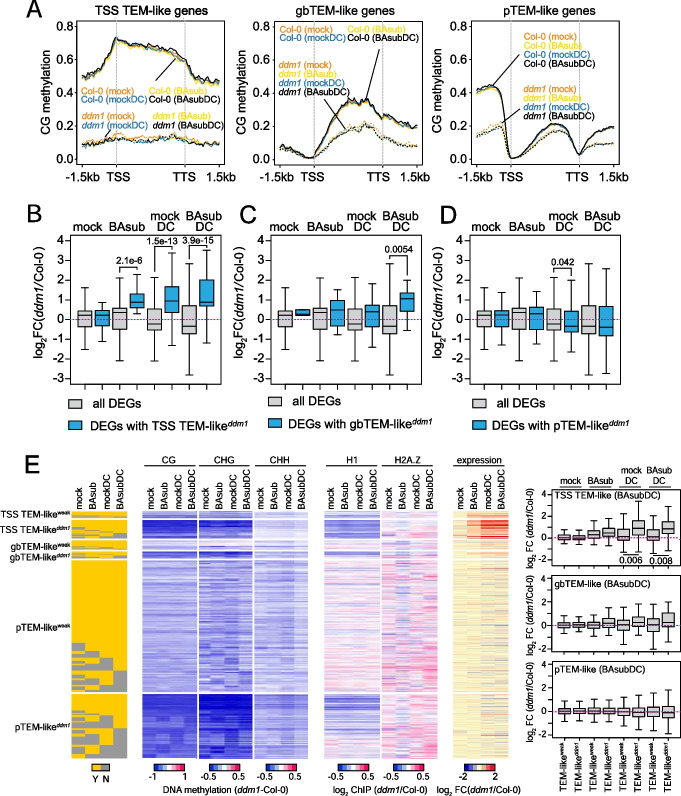
DDM1-mediated transposon-like methylation (TEM-like) at transcription start sites (TSS) and gene bodies is associated with augmented gene expression during defense response under sub-optimal priming conditions (BAsubDC). **A** Averaged CG DNA methylation level around genes with TEM-like at TSS, gene bodies, or promoters. **B–D** Boxplots of gene expression of DEGs with strongly demethylated TEM-like in *ddm1* (TEM-like^*ddm1*^) at TSS, gene body, and promoter (TSS TEM-like^*ddm1*^, gbTEM-like^*ddm1*^, pTEM-like^*ddm1*^). Expression fold change (log_2_ (*ddm1*/Col-0)) of all DEGs at each condition was plotted as control. DEG, differentially expressed gene. **E** CG, CHG, CHH methylation, histone H1, H2A.Z, and gene expression profiles of genes with TEM-like^*ddm1*^ at TSS, gene body, and promoter (TSS TEM-like^*ddm1*^, gbTEM-like^*ddm1*^, pTEM-like^*ddm1*^) and TEM-like^weak^ at TSS, gene body, and promoter (TSS TEM-like^weak^, gbTEM-like^weak^, pTEM-like^weak^) in Col-0 and *ddm1* under the four treatment conditions. TEM-like^*ddm1*^; strongly demethylated TEM-like in *ddm1*, TEM-like^weak^; TEM-like that their methylation levels are weakly affected or not affected in *ddm1*. **B–E** *p*-values by Student’s *t* test

TEs can exist in the promoter and gene body to affect expression of nearby genes. Thus, we next investigated whether TEM-like^*ddm1*^ is associated with gene expression in a weakly primed defense response. To precisely evaluate the unique effect of DDM1-mediated TEM-like (TEM-like^*ddm1*^) at promoter, transcription start site (TSS), or gene body to gene expression, we selected the following three mutually exclusive groups of genes (Additional file 1: Fig. S4A): (1) 4702 genes with TEM-like only at the promoter (pTEM-like), (2) 1250 genes with TEM-like only at TSS (TSS TEM-like), (3) 1187 genes with TEM-like only at gene body (gbTEM-like). We then examined the DNA methylation patterns of these three gene groups in Col-0 and *ddm1* plants over the gene structure under four treatment conditions (mock, BAsub, mockDC, and BAsubDC) (Fig. 4A). The CG and nonCG methylation patterns around genes were similar across treatment conditions (Fig. 4A and Additional file 1: Fig. S4B–S4D). Correspondingly, the methylation difference between *ddm1* and Col-0 over the gene structure were similar across different conditions with high correlation coefficients (*r* = 0.73 ~ 0.94; Additional file 1: Fig. S4E–S4G).

Next, we analyzed the effects of the three gene groups on gene expression. To this end, we first selected all DEGs between *ddm1* and Col-0 plants in each treatment condition (e.g., BAsubDC). From the three gene groups, we then selected the DEGs with demethylated pTEM-like (pTEM-like^*ddm1*^), TSS TEM-like (TSS TEM-like^*ddm1*^), and gbTEM-like (gbTEM-like^*ddm1*^) in *ddm1* (vs. Col-0) under the treatment condition (e.g., BAsubDC). We next compared the expression changes of all DEGs and DEGs with each of the three TEM-like^*ddm1*^ (Fig. 4B–D and Additional file 2: Supplementary data 1) in *ddm1* (vs. Col-0) under the treatment condition (e.g., BAsubDC). When one of the three TEM-like^*ddm1*^ had strong effects on gene expression, DEGs with the TEM-like^*ddm1*^ would show higher expression changes than all DEGs. This procedure was performed for all four treatment conditions (mock, BAsub, mockDC, and BAsubDC). DEGs with TSS TEM-like^*ddm1*^ were hyperactivated under BAsub, mockDC, and BAsubDC conditions, although they showed no changes under the mock condition, compared to all DEGs (Fig. 4B). Also, DEGs with gbTEM-like^*ddm1*^ were hyperactivated predominantly in the BAsubDC condition (Fig. 4C). In contrast, pTEM-like^*ddm1*^ did not affect gene expression, except under the mockDC condition (Fig. 4D).

We then integrated DNA methylation, H1, H2A.Z, and gene expression levels at TEM-like under the four conditions to examine correlations of DNA methylation, H1, and H2A.Z changes with expression changes of genes with the above three groups of TEM-like^*ddm1*^ or TEM-like^weak^. Among the three methylation contexts, genes with TSS TEM-like^*ddm1*^ showed hyperactivation, which was much stronger than genes with TSS TEM1-like^weak^ (Fig. 4E, expression and the boxplot on top), and CG demethylation was stronger in these genes than CHG demethylation while CHH methylation showed no significant changes (Fig. 4E, CG, CHG, and CHH), consistent with the finding in Fig. 3A. Moreover, H2A.Z levels showed no correlation with hyperactivation of genes with TSS TEM-like^*ddm1*^ while H1 showed a partial correlation (Fig. 4E and Additional file 1: Fig. S4H, H1 and H2A.Z), consistent with the finding in Fig. 3G. Taken together, TSS TEM-like^*ddm1*^ and gbTEM-like^*ddm1*^, to a lesser extent, augmented transcription especially under the BAsubDC condition.

### DDM1-mediated GBM potentiates transcription

**Fig. 5:**
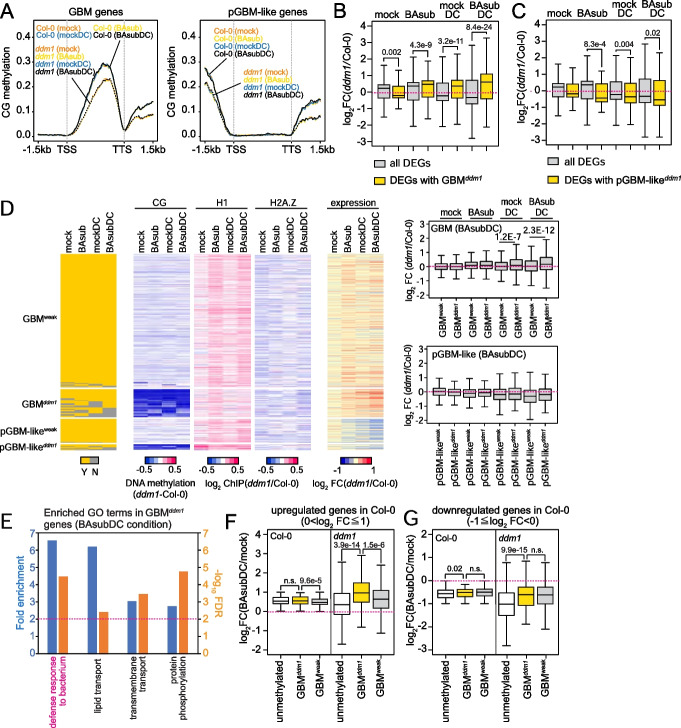
DDM1-mediated gene body methylation is associated with augmented gene expression during defense response under sub-optimal priming conditions. **A** Averaged CG DNA methylation level around genes with GBM-like at gene bodies (GBM) or promoters (pGBM-like). **B,C** Boxplots of gene expression of DEGs with strongly demethylated GBM-like in *ddm1* (GBM-like^*ddm1*^) at gene body and promoter (GBM^*ddm1*^, pGBM-like^*ddm1*^). Expression fold change (log_2_ (*ddm1*/Col-0)) of all DEGs at each condition was plotted as control. DEG, differentially expressed gene. **D** CG methylation, histone H1, H2A.Z, and gene expression profiles of genes with GBM-like^*ddm1*^ at gene body and promoter (GBM^*ddm1*^, pGBM-like^*ddm1*^) and GBM-like^weak^ at gene body and promoter (GBM^weak^, pGBM-like^weak^) in Col-0 and *ddm1* under the four treatment conditions. GBM-like^*ddm1*^; strongly demethylated GBM-like in *ddm1*, GBM-like^weak^; GBM-like that their methylation levels are weakly affected or not affected in *ddm1*. **E** Top 4 enriched GO terms among genes with GBM^*ddm1*^ under BAsubDC condition. **F,G** Boxplots of gene expression fold change (log_2_ (BAsubDC/mock)) in Col-0 and *ddm1* plants. Genes subtly upregulated (**F**) and downregulated (**G**) in Col-0 were grouped and their expression fold change in Col-0 and *ddm1* were plotted. unmethylated; unmethylated genes in both Col-0 and *ddm1*, GBM^*ddm1*^; genes with strongly demethylated GBM in *ddm1* (vs. Col-0, BAsubDC condition), GBM^weak^; genes with GBM that their methylation levels are weakly affected or not affected in *ddm1* (vs. Col-0, BAsubDC condition). **B–D, F,G** *p*-values by Student’s *t* test. n.s.; not significant (*p* > 0.05)

We next investigated the effect of GBM-like^*ddm1*^ on gene expression. GBM-like can also occur in the promoter and TSS to affect expression of target genes. Similar to genes with TEM-like^*ddm1*^, we thus selected three mutually exclusive groups of genes (Additional file 1: Fig. S5A): (1) 2157 genes only with pGBM-like, (2) 83 genes only with TSS GBM-like, and (3) 6816 genes only with gbGBM-like (i.e., GBM). The TSS GBM-like gene group, including only 83 genes, was excluded for the downstream analyses because they may provide insufficient statistical power. Like TEM-like, CG methylation levels of the GBM-like were reduced in *ddm1* plants and showed similar patterns over gene structure across the treatment conditions (Fig. 5A).

We compared the expression changes of all DEGs and DEGs with pGBM-like^*ddm1*^ or GBM^*ddm1*^ (Fig. 5B,C and Additional file 2: Supplementary data 1). DEGs with GBM^*ddm1*^ were decreased compared to all DEGs under the mock condition, but hyperactivated under the BAsub, mockDC, and BAsubDC conditions (Fig. 5B). In contrast, DEGs with pGBM-like^*ddm1*^ tended to be suppressed compared to all DEGs while they showed no expression changes under the mock condition (Fig. 5C). Similar to TEM-like, we integrated DNA methylation, H1, H2A.Z, and gene expression levels at GBM-like regions under the four conditions to examine correlations of DNA methylation, H1, and H2A.Z changes with expression changes of genes with the above two groups of GBM-like^*ddm1*^ or GBM-like^weak^. The H1 levels showed no correlation with hyperactivation of genes with GBM^*ddm1*^, consistent with the finding in Fig. 3H (Fig. 5D and Additional file 1: Fig. S5B). The H1 and H2A.Z levels were both reduced at GBM^*ddm1*^ genes than GBM^weak^ genes under BAsubDC condition (*ddm1* vs. Col-0, Fig. 5D and Additional file 1: Fig. S5B), suggesting that H1 or H2A.Z levels might negatively correlate with hyperactivation of GBM^*ddm1*^ genes. Therefore, we further tested whether H1 or H2A.Z levels correlate with the expression change of GBM^*ddm1*^ genes (Additional file 1: Fig. S5C). We grouped GBM^*ddm1*^ genes under BAsubDC condition into three: (1) GBM^*ddm1*^ genes hyperactivated in *ddm1* compared to Col-0 (*ddm1* hyper GBM^*ddm1*^; overlap with *ddm1* hyper genes from Fig. 2A); (2) GBM^*ddm1*^ genes suppressed in *ddm1* compared to Col-0 (*ddm1* sup GBM^*ddm1*^; overlap with *ddm1* sup genes from Fig. 2A); (3) GBM^*ddm1*^ genes with no expression change (noDEG GBM^*ddm1*^, adj. *p*-value > 0.1, Col-0 vs. *ddm1*). We then compared H1 and H2A.Z level change in *ddm1* plants (vs. Col-0) under four conditions in three groups (Additional file 1: Fig. S5C). There was a significant difference in H1 level change between “noDEG” and “*ddm1* hyper” groups under BAsubDC. However, no significant difference between “*ddm1* hyper” and “*ddm1* sup” groups indicates that such difference appears to be not systematic. Moreover, H1 levels at “*ddm1* hyper” groups showed no changes across the conditions compared to Col-0 except for BAsub condition. H2A.Z levels at “*ddm1* hyper” and “noDEG” groups changed similarly under four conditions, indicating minimal variations of H2A.Z among the DEG and noDEG groups, which are consistent with the findings from Fig. 3H above. Together with the results from TEM-like^*ddm1*^, TSS TEM-like^*ddm1*^, gbTEM-like^*ddm1*^, and GBM^*ddm1*^ had significant effects on gene expression especially under the BAsubDC condition, and H1 and H2A.Z levels did not show significant association with gene expression changes at these genes.

### GBM^*ddm1 *^is associated with defense response

We next examined whether genes with TSS TEM-like^*ddm1*^, gbTEM-like^*ddm1*^, or GBM^*ddm1*^ under BAsubDC were associated with defense response. To this end, we performed the enrichment analysis for these genes and found that “defense response to bacterium” genes were significantly enriched in genes with GBM^*ddm1*^ (Fig. 5E). Unlike genes with GBM^*ddm1*^, however, genes with TSS TEM-like^*ddm1*^ or gbTEM-like^*ddm1*^ had no enriched GO terms because a majority of them are pseudogenes or transposons with no GO terms annotated. Based on these results, we focused on the genes with GBM^*ddm1*^ hereafter. The enrichment of defense response raises a possibility that defense-related genes with GBM^*ddm1*^ may lead to hyperactivation upon DC inoculation. To test this possibility, we selected genes that showed subtle activation [0 < log_2_(fold change) ≤ 1, *p* ≤ 0.01] or suppression [ –1 ≤ log_2_(fold change) < 0, *p* ≤ 0.01] in Col-0 plants under the BAsubDC condition compared to under the mock condition. Among these genes showing subtle activation in Col-0 under BAsubDC, we then selected three groups of genes: (1) unmethylated genes, (2) genes with GBM^*ddm1*^, and (3) genes with GBM^weak^ (Additional file 2: Supplementary data 1). We then examined which gene groups showed hyperactivation in *ddm1* plants. While all three gene groups showed subtle activation in Col-0 under BAsubDC (Fig. 5F, left), genes with GBM^*ddm1*^ were more significantly hyperactivated in *ddm1* under the BAsubDC condition than unmethylated genes and genes with GBM^weak^ (Fig. 5F, right), suggesting that GBM could suppress the transcriptional potential of these genes in Col-0 plants. The same grouping and analysis were done for the genes with subtle suppression in Col-0 under BAsubDC. Unmethylated genes showed the most significant suppression, and genes with GBM^*ddm1*^ showed similar extents of suppression to genes with GBM^weak^ in *ddm1* plants, suggesting that GBM demethylation had limited effects on expression of suppressed genes under BAsubDC (Fig. 5G, right). Taken together, GBM^*ddm1*^ augments transcriptional activation of defense-related genes under BAsubDC.

### Unconventional GBM genes are demethylated in *ddm1*

**Fig. 6:**
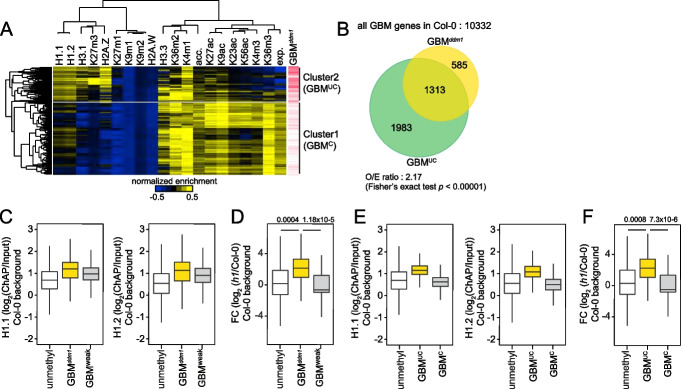
DDM1 mediates gene body methylation at genes with unconventional chromatin features. **A** Heatmaps of normalized histone variant levels, modified histone H3 levels, gene expression (exp.), and DNA accessibility (acc.) in 10,332 gene body methylated genes in Col-0. Data were centered to 0 and normalized. GBM^UC^; unconventional GBM cluster genes, GBM^C^; conventional GBM cluster genes. Red lines indicate overlap to genes with strongly demethylated GBM in *ddm1* (GBM^*ddm1*^). **B** Venn diagram of genes with strongly demethylated GBM in *ddm1* (GBM^*ddm1*^) and unconventional GBM cluster genes (GBM^UC^). *p*-value by Fisher’s exact test. **C** Boxplots of H1 levels of unmethylated genes in both Col-0 and *ddm1* (unmethyl), genes with strongly demethylated GBM in *ddm1* (GBM^*ddm1*^), and genes with GBM that their methylation levels are weakly affected or not affected in *ddm1* (GBM^weak^). **D** A boxplot of gene expression fold change (FC) of differentially expressed genes in *h1* plants (compared to Col-0, *q*-value < 0.05) overlapping with unmethylated genes in both Col-0 and *ddm1* (unmethyl), genes with strongly demethylated GBM in *ddm1* (GBM^*ddm1*^), and genes with GBM that their methylation levels are weakly affected or not affected in *ddm1* (GBM^weak^). *p*-values by Student’s *t* test. **E** Boxplots of H1 levels of unmethylated genes both Col-0 and *ddm1* (unmethyl), unconventional GBM cluster genes (GBM^UC^), and conventional GBM cluster genes (GBM^C^). **F** A boxplot of gene expression fold change (FC) of differentially expressed genes in *h1* plants (compared to Col-0, *q*-value < 0.05) overlapping with unmethylated genes in both Col-0 and *ddm1* (unmethyl), unconventional GBM cluster genes (GBM^UC^), and conventional GBM cluster genes (GBM^C^). *p*-values by Student’s *t* test

We then investigated the chromatin environment at 10,332 genes with CG methylation at gene body in Col-0. We calculated average levels of histone variants (H1.1, H1.2, H2A.W, H2A.Z, H3.1, and H3.3), histone H3 methylation (H3K4me1 and me3, H3K9me1 and me2, H3K27me1 and me3, and H3K36me2 and me3), and histone H3 acetylation (H3K9ac, H3K23ac, H3K27ac, and H3K56ac). We also measured average DNA accessibility and RNA expression (Fig. 6A, acc. and exp.). Unsupervised hierarchical clustering using the average levels of these chromatin features revealed two clusters (Clusters 1–2) of the genes with GBM (Fig. 6A). Cluster 1 was enriched with the genes with high levels of H3K36me3 methylation, an active chromatin mark to suppress cryptic transcriptional initiation within gene bodies in eukaryotes [56], called conventional GBM cluster (Fig. 6A, GBM^C^). These genes with GBM^C^ had high levels of H3 acetylation and H3K4me3, consistent with their high RNA expression. As GBM and H2A.Z are mutually exclusive, these genes were depleted of H2A.Z (Fig. 6A). Cluster 2 was enriched with the genes with vastly variable chromatin characteristics, called unconventional GBM cluster (Fig. 6A, GBM^UC^). These genes with GBM^UC^ had relatively low levels of RNA expression and transcription-associated histone markers (H3 acetylation, H3K4me3, and H3K36me3). Also, these genes had high levels of H1, H3.1, and H3K27me3, which are associated with silent genes or developmentally regulated genes [51, 57–59]. Moreover, these genes were enriched with GBM^*ddm1*^ (Fig. 6A, red lines), and 69% of GBM^*ddm1*^ genes overlapped with these genes with GBM^UC^ (Fig. 6B).

The large overlap between genes with GBM^*ddm1*^ and genes with GBM^UC^ prompted us to examine whether they had shared properties. Genes with GBM^*ddm1*^ were shorter than genes with GBM^weak^ and had low H3K36me3 levels (Additional file 1: Fig. S6A). H1 levels were higher in genes with GBM^*ddm1*^ than genes with GBM^weak^ (Fig. 6C), and loss of H1 increased expression of genes with GBM^*ddm1*^, indicating that H1 suppresses their expression (Fig. 6D, Additional file 2: Supplementary data 1). Previous reports suggest that GBM genes with high H2A.Z are associated with greater transcriptional responsiveness to stress [32, 60]. Consistent with this, genes with GBM^*ddm1*^ had high H2A.Z levels and were more responsive to external and developmental stimuli than genes with GBM^weak^ (Additional file 1: Fig. S6B). Their expression tended to be more variable than that of genes with GBM^weak^ (Additional file 1: Fig. S6B), which exhibited stable expression like housekeeping genes [32, 34, 35, 37]. As expected, all these properties were shared with genes with GBM^UC^ (Fig. 6E,F and Additional file 1: Fig. S6C-D).

Genes with GBM^*ddm1*^ were enriched with H2A.Z compared to genes with GBM^weak^, similar to genes with GBM^UC^. However, changes of H2A.Z levels at GBM-like regions were not correlated with GBM changes in *ddm1* plants (Fig. 3H), suggesting the limited role of H2A.Z in the augmented activation of genes with GBM^*ddm1*^ during defense response. In support, *h2a.z* triple plants did not show altered disease resistance under BAsubDC compared to mockDC (Additional file 1: Fig. S6E). Moreover, the basal expression levels of genes with GBM^UC^ were elevated in *met1* and *h1met1* plants than genes with GBM^C^ (Additional file 1: Fig. S6F, Additional file 2: Supplementary data 1), suggesting that GBM suppresses the expression of genes with GBM^UC^, but not genes with GBM^C^. Given the shared chromatin properties between genes with GBM^UC^ and GBM^*ddm1*^, this result is consistent with our finding that GBM suppresses defense-related genes with GBM^*ddm1*^ having high responsiveness to BAsub during defense response.

### DDM1-mediated GBM gene contributes to defense response priming

To identify priming regulator candidates regulated by DDM1, we first selected *ddm1* hyper genes with GBM^*ddm1*^ or GBM^weak^. The latter genes were included as potential indirect targets of DDM1. We then built a defense priming–associated network model (Additional file 1: Fig. S7) describing the interactions among the selected genes [61]. From the interaction network, among 237 *ddm1* hyper GBM^*ddm1*^ genes and 1754 *ddm1* hyper GBM^weak^ genes, we selected 133 hub-like genes (Additional file 3: Supplementary data 2). A subnetwork model for the hub-like molecules (Additional file 1: Fig. S7) included defense-related processes: (1) receptor candidates during the defense response, such as receptor-like kinases (CRKs) and leucine-rich repeat domain-containing proteins; (2) their downstream signaling components (CIPKs, CPKs, lectin protein kinases, MAP kinases, and other protein kinases); (3) defense-related hormone response factors, such as factors related to salicylic acid (NPR1 and ICS1), ethylene (EIN3), abscisic acid (ABI1, RAF11, and ABA3), and brassinosteroid (BRL3) hormone signaling; and (4) stress response factors (response to heat stress and reactive oxygen species). Among these 133 hub-like molecules, we selected five priming regulator candidates involved in the above defense-related processes and whose loss-of-function mutants are available (*GPK1*, *CPK4*, *KIN11*, AT1G17230, and *BRL3*). Two of these genes had GBM^*ddm1*^ (*GPK1* and *CPK4*) while and the other three (*KIN11*, *AT1G17230*, and *BRL3*) had GBM^weak^.

**Fig. 7:**
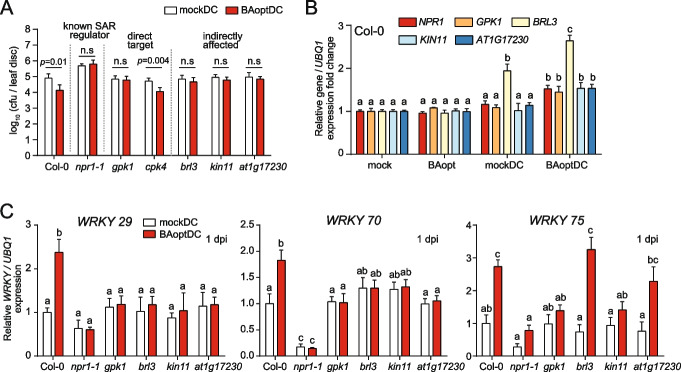
Hyperactivated network hub genes in *ddm1* contribute to primed defense response. **A** Bacterial growth in the mutants of five hyperactivated network hub genes in *ddm1* during defense response with optimal priming. We included *npr1-1* as a positive control. Experiments were performed as described in the legend of Fig. 1, except that a higher concentration of BABA (BAopt; 10 ml of 35 μg/ml per plant) was applied 3 days before DC inoculation to fully prime plants. *p*-values by Student’s* t* test. n.s.; not significant (*p* > 0.05). Error bars indicate SEM (*n* ≧ 5). SAR, systemic acquired resistance. cfu, colony-forming units. **B** Gene expression levels of the putative primed defense regulators and *NPR1* in Col-0 under four treatment conditions. Different letters indicate significant differences at *p*≦0.05 from two-way ANOVA within each gene, compare treatment conditions with Tukey’s correction (*α* = 0.05). Error bars indicate SEM (*n* ≧ 3). **C** *WRKY* gene expression in putative primed defense regulator mutants and *npr1-1* with or without priming. Gene expression was measured 1-day post-inoculation (dpi). *UBQ1* expression was used as an internal control. Different letters indicate significant differences at *p*≦0.05 from two-way ANOVA with Tukey’s correction (*α* = 0.05). Error bars indicate SEM (*n* = 4)

Using reverse-transcription quantitative PCR (RT-qPCR) analysis, we first confirmed their upregulation in *ddm1* plants under the BAsubDC condition and the lack of changes under the mock condition (Additional file 1: Fig. S8). We then obtained loss-of-function mutants of the five candidates, as well as mutants of the non-hub-like genes *AAO3*, *AT1G61690*, and *ZAR1* in the priming-associated network model as negative controls and a known systemic acquired resistance regulator, *NPR1*, as a positive control [62, 63] (Additional file 1: Fig. S8). To test the function of priming regulator candidates through their mutants in Col-0 background, we used a higher dose of BABA (10 ml of 35 μg/ml BABA; BAopt) than that used for weak priming in the BAsub condition, which fully primes the defense response in Col-0 (BAoptDC in Additional file 1: Fig. S1A; BAopt, BAoptDC in Additional file 1: Fig. S1B). Under such condition, whether the mutants of the candidates compromise BABA-induced priming can be effectively evaluated. Compared to mock samples, fully primed Col-0 plants exhibited decreased bacterial growth and upregulated *WRKY* expression, indicating that defense priming was induced in Col-0 under the BAopt condition (Fig. 7A and Additional file 1: Fig. S9A). *npr1* plants were more susceptible to DC, and the BAopt treatment failed to induce disease resistance (Fig. 7A). *gpk1*, *brl3*, *kin11*, and *at1g17230* plants did not alter resistance to DC without BAopt treatment but failed to induce resistance by chemical priming (Fig. 7A; BAopt, *p* > 0.05). In contrast, knockout of *cpk4* and three non-hub-like genes did not affect chemical priming of disease resistance against DC (Fig. 7A, Additional file 1: Fig. S9B and S10).

Furthermore, the expression of *NPR1*, *GPK1*, *KIN11*, and *AT1G17230* was upregulated (*p* ≤ 0.05) in Col-0 only when DC was inoculated after BAopt treatment (BAoptDC condition) (Fig. 7B), suggesting that their expression is hyperactivated during chemically primed defense response. *BRL3* expression was activated in the mockDC condition but further hyperactivated in the BAoptDC condition. We also examined *WRKY* expression in the *gpk1*, *brl3*, *kin11*, and *at1g17230* knockout plants (Fig. 7C). All three *WRKY* genes failed to be hyperactivated by BAoptDC in *gpk1*, whereas *brl3* and *at1g17230* plants showed hyperactivation of *WRKY75* by BAoptDC. Further research is required to elucidate the relationship among these candidates, such as their genetic hierarchy and redundancy.

### Unconventional, DDM1-mediated GBM genes have variable GBM levels within natural populations

**Fig. 8:**
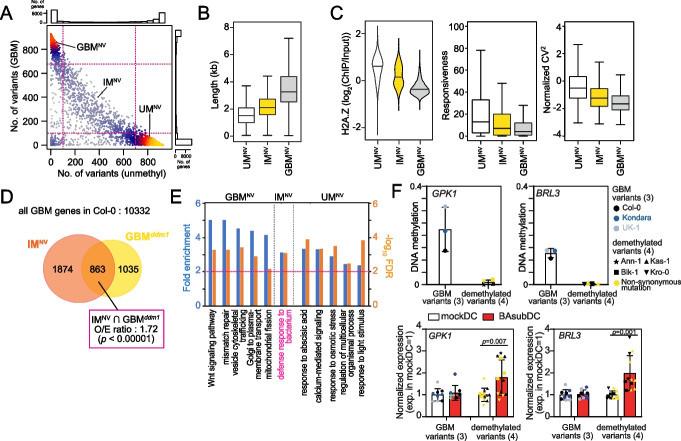
Genes with highly variable gene body methylation in the natural population are overlapped with DDM1-mediated gene body methylation. **A** Scatter plot of the number of natural variants of which methylation status is unmethylated (*x*-axis) or GBM (*y*-axis). Each dot represents a gene. The yellow color indicates the highest density, and blue means the low density of genes. GBM^NV^; genes with stable GBM over 700 natural variants, IM^NV^; genes with interchangeable GBM (possess GBM at 100–700 natural variants), UM^NV^; genes unmethylated in over 700 natural variants. Marginal histograms show the distribution for the number of genes. **B** A boxplot of gene length of gene groups in **A**. **C** A violin plot and boxplots of H2A.Z level, responsiveness, and normalized CV^2^ of gene groups in **A**. Responsiveness score and normalized CV^2^ were calculated as Aceituno et al. [69] and Cortijo et al. [60]. **D** Venn diagram of DDM1-mediated GBM genes (GBM^*ddm1*^) and genes with interchangeable DNA methylation in the natural population (IM^NV^) [64, 68]. *p*-value by Fisher’s exact test. **E** Top 5 enriched GO terms of gene groups in **A**. **F** Relative expression of novel priming regulators in natural variants. (Top) The average GBM level of *GPK1* and *BRL3* in GBM variants and demethylated variants. (Bottom) Expression of *GPK1* or *BRL3* under mockDC condition was normalized to 1. Six natural variants and Col-0 were grouped by their GBM level at *GPK1* and *BRL3*. Col-0, Kondara, and UK-1 maintained GBM at both genes, and Ann-1, Bik-1, Kas-1, and Kro-0 lost GBM at *GPK1* and *BRL3*. RT-qPCR data from three replicates per natural variant were averaged (*n* = 9 for GBM variants, *n* = 12 for demethylated variants). Yellow color indicates demethylated variants with non-synonymous mutation in each gene (Bik-1, Kro-0 in *GPK1*; Ann-1, Kas-1 in *BRL3*). Error bars indicate standard deviation. *p*-values by Student’s *t* test

CG methylation is reported to be variable among natural populations [64–67]. Therefore, we investigated the variability of GBM among a natural population of 927 individuals collected from different places in the world [64, 68]. To simplify the analysis, we identified two groups of genes in each individual based on their average methylation levels in different cytosine contexts: genes with (1) unmethylated gene body (CG ≦ 0.05, CHG ≦ 0.05, and CHH ≦ 0.02) and (2) GBM (CG ≧ 0.1, CHG ≦ 0.05, and CHH ≦ 0.02). Among 27,445 annotated genes, 7055 genes retained GBM in more than 700 natural variants (GBM^NV^; 26%) while 11,495 genes remained unmethylated in more than 700 natural variants (UM^NV^; 42%). There were 4603 genes (17%) with GBM in 100–700 natural variants, indicating that GBM is variable in the natural population (interchangeable GBM (IM^NV^); Fig. 8A).

GBM^NV^ genes were long and highly expressed, consistent with the previously reported characteristics of genes with GBM [70, 71] (Fig. 8B and Additional file 1: Fig. S11A). IM^NV^ genes were shorter than GBM^NV^ genes (Fig. 8B). IM^NV^ genes had higher H1 and H2A.Z levels, and their expression was more responsive to various stresses and more variable than that of GBM^NV^ genes (Fig. 8C). Expression of genes with GBM^*ddm1*^ was also more responsive and variable than that of genes with GBM^weak^ (Additional file 1: Fig. S6B). These results imply that IM^NV^ genes have similar characteristics to genes with GBM^*ddm1*^, which prompted us to test whether the DNA methylation at IM^NV^ genes is dependent on DDM1. We first isolated IM^NV^ and GBM^NV^ genes containing GBM in Col-0. Then we calculated the overlap between IM^NV^ and genes with GBM^*ddm1*^, between GBM^NV^ and genes with GBM^weak^ in Col-0. IM^NV^ genes significantly (*p* < 1 × 10^−5^) overlapped with genes with GBM^*ddm1*^ (Fig. 8D). In contrast, GBM^NV^ genes significantly (*p* < 1 × 10^−5^) overlapped with GBM^weak^ genes (Additional file 1: Fig. S11B). Following GO term analysis of each gene group (GBM^NV^, IM^NV^, and UM^NV^), we established that only “defense response to bacterium” genes were enriched in the IM^NV^ genes (Fig. 8E). Among the aforementioned priming regulators, *GPK1* and *BRL3* belonged to IM^NV^ (Additional file 1: Fig. S11C). These results suggest that modulation of DDM1 activity can affect the GBM levels of defense-related genes in IM^NV^ group, which may serve as an adaptation strategy to modulate responsiveness to pathogens in natural populations.

To test whether variable GBM levels of the priming regulators could modulate transcriptional responsiveness to pathogens in natural variants, we first investigated whether the regulators had GBM in 927 natural variants and found that 87 variants showed demethylation (average gene mCG < 0.05) at more than two regulators while 172 variants had GBM at all four regulators [64]. Among these variants, we randomly selected four natural variants (Ann-1, Bik-1, Kas-1, Kro-0) that both *GPK1* and *BRL3* were demethylated and two natural variants (UK-1 and Kondara) that had GBM at all the regulators (average gene mCG ≥ 0.1). We then examined the expression of four regulators under the BAsubDC condition in six natural variants and Col-0 (Fig. 8F). As expected, *GPK1* and *BRL3* showed augmented expression under BAsubDC only in variants that lost GBM (Fig. 8F, vs. mockDC). *AT1G17230* and *KIN11* maintained GBM in all six natural variants we tested, suggesting no significant transcriptional change in BAsubDC compared to in mockDC (Additional file 1: Fig. S11D). Finally, when we checked the expression of novel priming regulators in *h1, met1*, and *h1met1* plants, we found that *GPK1* and *BRL3* expression was upregulated by loss of CG methylation (Additional file 1: Fig. S11E), again suggesting that GBM at the priming regulators suppresses their expression.

There are possibilities that polymorphisms or histone modification in natural variants could affect augmented expression of the priming regulators. To test these possibilities, we first examined polymorphisms of natural variants. Although there are single-nucleotide polymorphisms in natural variants (Bik-1 and Kro-0 in *GPK1*, Ann-1 ad Kas-1 in *BRL3*), transcriptional differences of *GPK1* and *BRL3* among Col-0 and the natural variants were not observed in the mock condition (Additional file 1: Fig. S11F, G). We also investigated H3 histone modification patterns around the priming regulators in four natural variants (Ann-1, Bik-1, Kas-1, Kro-0). Except H3K27me3 of *AT1G17230* in Bik-1, H3 histone modification patterns in the four demethylated natural variants at the priming regulator genes were comparable to those of Col-0 (Additional file 1: Fig. S12A). These results suggest that polymorphisms or H3 histone modifications at the priming regulator genes are not associated with transcriptional potentiation of the priming regulator genes in natural variants. We further investigated H3 histone modification patterns around the priming regulators (*GPK1*, *BRL3*, *AT1G17230*, and *KIN11*) in *ddm1* plants under mockDC and BAsubDC conditions (Additional file 1: Fig. S12B-D). H3K4me3 levels were significantly higher in *ddm1* plants than Col-0, but H3K4me3 levels under mockDC and BAsubDC conditions showed no correlation with their expression changes in *ddm1* (Additional file 1: Fig. S12B). GPK1, BRL3, and KIN11 accumulated more H3K27me3 in *ddm1* (Additional file 1: Fig. S12C), and GPK1 and AT1G17230 had more H3K9me2 in *ddm1* plants (Additional file 1: Fig. S12D). Because H3K27me3 and H3K9me2 are repressive histone markers, their elevated levels in *ddm1* are not correlated with the hyperactivation of priming regulators. Therefore, H3 histone modification patterns appear not to be associated with the transcriptional potentiation of priming regulators in *ddm1*.

## Discussion

A recent study [72] in oil palm plants suggests that the demethylation of mCHG (“*Karma*” loci) within the gene body causes hyperactivation of a novel splice form of the *DEF1* gene. Intriguingly, DNA hypomethylation is stable throughout the inflorescence development, but the expression of *DEF1* is induced only after stage 3 during inflorescence development, even given the demethylation at *Karma* loci. Similarly, demethylation of TE-like methylation at gene bodies was associated with hyperactivation of demethylated genes under BAsubDC condition, but it did not affect steady-state gene expression (Fig. 4C). This is consistent with hyperactivation of GBM-demethylated genes in *ddm1* plants only after BAsub treatment or inoculation with DC (Fig. 5B). These results suggest that DNA hypomethylation in the gene body only potentiates transcription of defense-related genes, and these genes are finally induced when plants encounter sufficient defense signals to overcome the threshold set by the degree of DNA hypomethylation.

Histone variants might be involved in the augmented expression of genes with GBM^*ddm1*^ upon pathogen attack along with DNA methylation. However, we could not find apparent links of H1 and H2 variants (H2A.Z, and H2A.W) to GBM-related transcriptional potentiation in *ddm1* (Figs. 3, 4, and 5, Additional file 1: Fig. S3 to S6). H3 histone modification patterns around the priming regulators (*GPK1*, *BRL3*, *AT1G17230*, and *KIN11*) showed no apparent association with gene expression changes in *ddm1* (Additional file 1: Fig. S12B-D). On the other hand, interestingly, the levels of the repressive marks H3K9me2 and H3K27me3 were increased in *ddm1* mutants and tended to increase further under BAsubDC condition, suggesting alternate possibilities that these marks could indirectly affect transcription through pathways that remain to be explored. We also investigated H3 histone modification patterns around the priming regulators in four natural variants (Ann-1, Bik-1, Kas-1, Kro-0) that both *GPK1* and *BRL3* were demethylated. Except for H3K27me3 of *AT1G17230* in Bik-1, H3 histone modifications in the four demethylated natural variants exhibited similar patterns at the priming regulator genes to those of Col-0 (Additional file 1: Fig. S12A). These results again suggest that H3 histone modifications do not affect the transcriptional potentiation of gene-body-demethylated priming regulator genes in natural variants.

Given little associations of histones with GBM-related transcriptional potentiation-associated defense priming response, although there could be other chromatin properties that we have not tested, we carefully propose a following model for GBM-related transcriptional potentiation. In *met1* plants, CG methylation is completely erased, thus GBM^*ddm1*^ genes are fully potentiated, resulting in spontaneous induction of these genes with zero threshold for induction. Therefore, *met1* plants showed enhanced disease resistance without priming, and sub-optimal priming further fortified defense response against DC (Fig. 1), and the expression of *GPK1* and *BRL3* was upregulated in these plants (Additional file 1: Fig. S11E), which further supports the idea that global loss of CG methylation enhanced transcriptional potential. In *ddm1* plants, because GBM is partially demethylated, resulting in a non-zero threshold for induction of GBM^*ddm1*^ genes. Thus, transcriptional induction of GBM^*ddm1*^ genes still require additional activation signal, but sub-optimal concentration of BABA is sufficient to overcome threshold for transcriptional induction (Figs. 1 and 5 and Additional file 1: Fig. S13). In Col-0 plants, there are basal levels of GBM, which are much higher than those of GBM in *ddm1*, resulting in a higher threshold for induction of genes than that in *ddm1*. The higher threshold thus requires a stronger activation signal, namely treatment of an optimal concentration (higher than sub-optimal concentration) of BABA (BAoptDC) for induction of defense-related genes (Additional file 1: Fig. S9, S10 and S13). However, a sub-optimal BABA treatment is not sufficient to overcome a high induction threshold of defense-related genes.

GBM is essentially invariable throughout plant development [73, 74], but it is relatively unstable over generations compared to the genetic mutation rate [75–77]. Therefore, the proportion of genes with GBM fluctuated from ~ 9 to 20% of all genes in *Arabidopsis* accessions [65]. DNA methylation levels of GBM^*ddm1*^ were variable among natural variants (Fig. 8). Because GBM^*ddm1*^ could affect the transcriptional potential of defense priming regulators, epigenetic variations on GBM^*ddm1*^ could be a strategy of plants to adopt environments with various stress levels.

Recently, Shahzad et al. showed mild, positive correlation between GBM and expression level at hundreds of conventional GBM genes in natural *Arabidopsis* populations [78]. In contrast, DDM1-mediated GBM did not affect the steady-state gene expression level but suppressed the inducibility of associated genes. Unlike conventional GBM genes, GBM^*ddm1*^ genes were enriched with H2A.Z, and their H1 levels are higher than conventional GBM genes (Fig. 6C and Additional file 1: Fig. S6B). These results indicate that GBM can play different roles depending on its chromatin environment. Consistent with this, euchromatic transposon methylation, which generally represses transcription, can activate gene expression when SU(VAR)3–9 homolog proteins (SUVH1 and SUVH3) bind to methylated DNA and recruit DNAJ proteins [79].

There are several reports that GBM might affect transcriptional elongation [80–82]. DNA methylation can modulate transcriptional elongation and splicing by inhibiting the binding of other proteins to methylated regions. For example, in mammalian cells, DNA methylation inhibits the binding of CTCF, which causes RNA polymerase II pausing and inclusion of weak exons [83], and MeCP2 binds to the methylated gene body to suppress transcription by impeding transcriptional elongation [84]. Therefore, GBM may affect elongation efficiency or occupancy of RNA polymerase II in the gene body of target genes, and loss of GBM enables the rapid induction of these genes upon pathogen challenge (Additional file 1: Fig. S13). The presence of GBM might hinder the effect of weak priming by BABA, and selective loss of GBM in *ddm1* plants enables efficient transcriptional elongation of target genes such as *GPK1* (Additional file 1: Fig. S13).

Gene body regions often contain functional elements, such as enhancers [85, 86]. Thus, decreased GBM of these elements in the *ddm1* mutant may contribute to the expression of the target genes. For example, GBM at enhancer elements suppresses the expression of the target genes in B-cells of chronic lymphocytic leukemia patients [87]. Lie et al. reported that the gene body enhancer of human epidermal growth factor receptor-2 (HER2) and hypomethylation of HER2 gene body enhancer region in breast cancers contribute to HER2 expression by increasing its accessibility to transcription factors [88]. The validity of these models needs further investigation. Since the constitutive activation of defense responses causes severe growth defects, it is essential to precisely control the timing of defense responses to maximize plant fitness. Regulating GBM level may contribute to enhancing the inducible activation of defense-related genes without unnecessary, constitutive activation of these genes.

## Conclusion

In this study, we report that DDM1-mediated GBM suppresses inducible activation of defense-related genes, and the loss of GBM in *ddm1* potentiates BABA-induced resistance. The augmented activation of defense-related genes in *ddm1* is associated with the loss of DDM1-mediated GBM. We also discover that knockout of candidate genes, which were hypomethylated in *ddm1* mutants, in Col-0 background can impair BABA-induced resistance. Unlike conventional GBM genes, genes with DDM1-mediated GBM have high H1 level, H2A.Z level, and variable expression. Furthermore, we find that genes with highly variable GBM in the natural population of *Arabidopsis* are significantly overlapped with DDM1-mediated GBM, and demethylation of *GPK1* in natural variants is associated with augmented transcription. Of note, DDM1-mediated TE-like methylation located in the transcription start site and gene body also augmented transcription during defense priming, but there is no significant enrichment of defense-related genes among them. Overall, we hypothesize that DDM1-mediated GBM modulates the inducibility of the immune response, functioning as a fundamental mechanism for faster and stronger defense induction upon pathogen challenge.

## Methods

### Plant materials and growth conditions

*Arabidopsis thaliana* wild-type (Col-0 and Ler-0), natural variants (UK-1, Kondara, Bik-1, Kro-0, Ann-1, Kas-1), and mutant seeds (F_1_ generation) were germinated on 1/2 × B5 medium. Seedlings were planted on soil 1 week after germination and grown under short-day conditions (22 °C, 10 h light/14 h dark) for 3 to 4 weeks. *cmt3-7* (CS6365), *met1-3* (CS16394), *ddm1* (SALK_000590), *drm1 drm2* (CS16383), *npr1-1* (CS3726), *gpk1* (SALK_047485C), *cpk4* (SALK_081860C), UK-1 (CS76620), Kondara (CS22651), Bik-1 (CS76449), Kro-0 (CS76533), Ann-1 (CS76438), Kas-1 (CS79018), *h2a.z* (CS69073) seeds were obtained from the Arabidopsis Biological Resource Center (ABRC). Dr. Jin Hoe Huh provided *ros1, ddc,* and *rdd* seeds, Dr. Hong Kyu Choi provided *kin11* seeds, Dr. Sunghwa Choe provided *brl3* seeds, Dr. Hiroo Fukuda provided *at1g17230* seeds, Dr. Olivier Mathieu provided *h2a.w* sees, and Dr. Steve Jacobsen provided *ddcc* seeds.

### BABA treatment and pathogen growth assays

To examine the defense priming phenotypes, gene expression, and DNA methylation level, 3–4-week-old plants were irrigated with 10 ml of 30 μg/ml *β*-aminobutyric acid (BABA), referred to as a sub-optimal priming condition (BAsub). Two days after the BAsub treatment, plants were sprayed with 40 ml of 5 × 10^7^ colony-forming units (cfu)/ml of *Pseudomonas syringae pv. tomato* strain DC3000 (DC) per pot (pot volume = 1200 ml; 12 plants per pot). For optimal priming, wild-type plants and mutant lines were irrigated with 10 ml of 35 μg/ml BABA (BAopt). Three days after the BAopt treatment, plants were sprayed with 40 ml of 5 × 10^7^ cfu/ml of DC per pot (pot volume = 1200 ml; 12 plants per pot). We performed pathogen growth assays 3 days post-inoculation, as previously described [89] with minor modifications. Leaf discs from three different leaves were ground and serially diluted at a ratio of 1:10 (v/v) in 10 mM MgCl_2_. Diluted samples were plated on King’s B medium.

### RNA isolation and reverse-transcription quantitative PCR (RT-qPCR)

Total RNA was isolated using Trizol (Invitrogen, cat. No. 15596026) according to the manufacturer’s instructions. Contaminating DNA in the isolated RNA was removed with Turbo DNA-free kit (Ambion, cat. no. AM1907). cDNA was produced with the ImProm-II Reverse Transcription System (Promega, cat. No. A3800). SYBR Premix Ex Taq (Takara, cat. No. RR420A) and LightCycler 2.0 (Roche, cat. No. 03531414001), StepOnePlus™ Real-Time PCR System (Applied Biosystems, cat. No. 4376600) were used for RT-qPCR. Primer sequences are listed in Additional file 4: Table S1.

### Chromatin immunoprecipitation quantitative PCR (ChIP-qPCR), ChIP sequencing, and sequence alignments

Chromatin immunoprecipitation (ChIP) assays were performed as previously described with minor modifications [90]. In brief, 3 ~ 4 g of 3 to 4-week-old plant leaves were crosslinked with 1% formaldehyde for 20 min under vacuum, quenched with 125 mM glycine and ground into fine powder in liquid nitrogen. Chromatins were isolated and sheared into 200- to 500-bp DNA fragments by sonication. The sonicated chromatin was immunoprecipitated with 2 μg of anti-H3K4me3 (Abcam, ab8580), anti-H3K9me2 (Abcam, ab1220), or anti-H3K27me3 (Abcam, ab6002) antibody and with 25 μl of Dynabeads Protein G (Invitrogen, 10003D) for 12 h at 4 °C with rotation. The precipitated chromatin DNA was then purified by phenol–chloroform-isoamyl alcohol extraction and recovered by ethanol precipitation. ChIP-qPCR was performed, and results were calculated as a percentage of input DNA. Primer sequences for ChIP-qPCR are listed in Additional file 4: Table S1. To construct ChIP sequencing library, the sonicated chromatin was immunoprecipitated with 5 μg of anti-H2A.Z (Abcam, ab4174) or anti-H1 (Agrisera, AS11 1801) antibody and with 40 μl of Dynabeads Protein G (Invitrogen, 10003D). ChIP sequencing libraries were constructed using NEBNext Ultra II DNA Library Prep Kit (New England Biolabs Inc, E7103) following the manufacturer’s instructions. Constructed libraries were sequenced using Illumina NovaSeq 6000 and paired-end reads were obtained at Geninus (https://www.kr-geninus.com). Sequenced reads were mapped with Bowtie [91]. We calculated average coverage of 100-bp windows by bedtools multicov [92]. The coverages were normalized by quantile using limma R package [93], log_2_ ChIP/input ratio were calculated using awk [94].

### Genome-wide bisulfite sequencing and sequence alignments

Total genomic DNA (gDNA) was extracted using acid phenol:chloroform and further purified by ethanol precipitation. To construct the bisulfite sequencing library, gDNA was sonicated into 500-bp to 1-kb fragments. 5ʹ-Monophosphorylated, blunt-ended, double-stranded gDNA fragments were generated using an End-It DNA End-Repair Kit (Lucigen (Epicentre), Cat. No. ER0720) following the manufacturer’s instructions. End-repaired DNA was ligated with cytosine-methylated Illumina adapters. An EpiTect Bisulfite Kit (Qiagen, cat. No. 59104) was employed twice for bisulfite conversion of adapter-ligated DNA following the manufacturer’s instructions. After conversion, libraries were amplified by PCR. The bisulfite sequencing libraries were sequenced for three biological replicates per condition using the Illumina HiSeq 2500 system. Sequencing was performed at the Vincent J. Coates Genomic Sequencing Laboratory at the University of California, Berkeley. Sequenced reads were mapped with the bs-sequel pipeline (https://zilbermanlab.net/tools/) that incorporates Bowtie [91] for sequence alignment. Bisulfite conversion rates were estimated to be higher than 99% using chloroplast DNA methylation levels.

### Microarray experiments and data analysis

For genome-wide gene expression analysis, total RNA was isolated from plants under mock, BAsub, mockDC, and BAsubDC conditions, as described above. The integrity of total RNA was evaluated using a Bioanalyzer 2100 (Agilent, cat. No. G2939BA). The RNA integrity of all samples was sufficient for gene expression analysis (RNA integrity number ≧ 8.5). RNA was reverse-transcribed, amplified, and then hybridized to the Arabidopsis 4 Oligo Microarray (Agilent, cat. No. G2519F-021169) according to standard Agilent protocols. The levels of mRNAs were measured for two biological replicates of plants under each condition. Probe intensities were obtained using the Agilent microarray scanner. Log_2_ intensities were normalized using quantile normalization [95]. To identify differentially expressed genes (DEGs) between *ddm1* and Col-0 plants, *p*-values were computed as previously described [96]. Briefly, (1) T-values were calculated from the two-tailed *t*-test for individual genes by assuming unequal variance; (2) log_2_ median differences were calculated for individual genes; (3) empirical null distributions of T-values and log_2_ median differences were generated from random permutations of all the samples; (4) adjusted *p*-values of T-values and log_2_ median differences observed for individual genes were computed using their corresponding empirical null distributions; (5) *p*-values from the *t*-test and log_2_ median difference test were combined using the Liptak-Stouffer’s Z method [97]. For gene groups in heatmap in Figs. 2 and Additional file 1: Fig. S2, genes with combined *p*-value≦0.01, *t*-test *p*-value≦0.1, and absolute log_2_ median difference≧0.58 (1.5-fold) in comparison between *ddm1* and Col-0 under four treatment condition (mock, BAsub, DC, BAsubDC) were defined as DEGs. Expression patterns were classified by the criteria described in Additional file 1: Fig. S2A. Briefly, expression patterns were categorized by the expression in the mock and BAsubDC condition (hyperactivated in *ddm1* (*ddm1* hyper), suppressed in *ddm1* (*ddm1* sup), consistently upregulated in *ddm1* plants (vs. Col-0), or consistently downregulated in *ddm1* plants (vs. Col-0). To analyze the association between DNA methylation and gene expression (Figs. 4 and 5 and Additional file 1: Fig. S5), we defined DEGs as the genes with a combined *p*-value≦0.01 to incorporate genes with subtle but significant expression change.

### Identification of gene body-like methylation and transposon-like methylation

To define gene body-like methylation (GBM-like) and transposon-like methylation (TEM-like) in *Arabidopsis*, we first identified significantly methylated 50-bp windows for each C context (CG, CHG, and CHH). To identify CG DNA methylated windows, 50-bp windows with significant CG methylation in wild-type plants (vs. *met1* plants; *p*≦10^−14^; Fisher’s exact test) were identified. To identify CHG and CHH DNA methylated windows, 50-bp windows with significant CHG or CHH methylation in wild-type plants (vs. *drm1 drm2 cmt2 cmt3* plants; *p*≦10^−4^ for CHG and *p*≦10^−8^ for CHH; Fisher’s exact test) were identified. The 50-bp windows with significant DNA methylation at each cytosine context were merged into a single window if the distance to the nearest methylated window (of same C context) was closer than 500 bp. GBM-like was defined as the merged CG methylated windows that do not overlap with CHG or CHH methylated windows and have CG≧0.1, CHG≦0.05, and CHH≦0.02 on average. GBM-like within the gene body that does not overlap with TSS was called GBM. To define TEM-like regions, we first unified merged CHG and CHH methylated windows, then identified regions that overlap with merged CG methylated windows. Only the windows with CG≧0.1, CHG≧0.05, and CHH≧0.02 were called TEM-like. Hypomethylated GBM-Like regions in *ddm1* plants (Fig. 3 and Additional file 1: Fig. S3) were defined as the regions with CG methylation loss in *ddm1*≦ − 0.1 (*p*≦0.001 by Fisher’s exact test (fisher_exact_test.pl in https://zilbermanlab.net/tools/)), and CG≦0.15 in *ddm1* plants. Hypomethylated TEM-like in *met1* and *ddm1* plants were defined as the regions with CG methylation loss in mutants≦ − 0.1, CHG loss≦ − 0.05, and CHH loss≦ − 0.02 (*p*≦0.001 by Fisher’s exact test), and CG methylation in mutants≦0.15, CHG≦0.1, CHH≦0.05. CHH hypomethylated TEM-like in *drm2* plants (vs. Col-0) were defined as the regions with CHH methylation loss in *drm2* plants≦-0.02 (*p*≦0.001 by Fisher’s exact test) and CHH in *drm2* plants≦0.05. To identify differentially methylated TEM-like, GBM-like, and GBM in relation to genes (Figs. 4 and 5 and Additional file 1: Fig. S4, S5), the sum of the number of methylated Cs and unmethylated Cs on multiple TEM-like or GBM-like/GBMs on a gene was calculated. Differentially methylated regions were identified as above.

### Categorization of genes with TEM-like and GBM-like loci

We first selected genes with TEM-like DNA methylation at the promoter, TSS, or gene body. To evaluate the unique effect of TEM-like DNA methylation at the promoter, TSS, and gene bodies on gene expression, we then removed genes with TEM-like DNA methylation at multiple regions (e.g., both promoter and gene body), resulting in pTEM-like, TSS TEM-like, and gbTEM-like (Fig. 4). The same selection procedure was used for genes with GBM-like DNA methylation (Fig. 5). Of note, if a gene had both TEM-like and GBM-like DNA methylation, this gene was excluded from our analysis.

### Selection of potential regulators of defense priming

To identify potential defense priming regulators with GBM, genes with GBM in Col-0 were isolated from *ddm1* hyper genes (Fig. 2A). We then constructed a network model describing interactions among the selected GBM genes based on the interactomes in iNID [61] and NetworkX [98]. Next, based on the network model, we further selected hub-like molecules with high numbers of interactors in the network model as previously described [61]. A subnetwork model describing interactions among the hub-like molecules was then extracted from the network model (Additional file 1: Fig. S7). Finally, we selected five of the 133 hub-like regulator candidates for which mutant lines were available.

### Functional enrichment analysis

PANTHER classification system was used to identify significantly enriched gene ontology terms (PANTHER GO-slim biological processes) in a list of genes (e.g., genes with TSS TEM-like) [99, 100]. GO terms with FDR≦0.01 and fold enrichment≧2 were considered as significantly enriched.

### Statistical testing

For statistical analysis for *t*-test and Fisher’s exact test, we used Microsoft Excel and the online software Easy Fisher Exact Test Calculator (accessed December 22, 2022-retrieved from https://www.socscistatistics.com/tests/fisher/default2.aspx). For ANOVA test, GraphPad Prism software was used.

## Supplementary Information

**Additional file 1: Figure S1.** Weakly primed defense response in *ddm1* and *met1* plants. **Figure S2.** Exploring gene expression change during weakly primed defense response in *ddm1* (vs. Col-0). **Figure S3.** Transposon-like methylationand (TEM-like) and gene body-like methylation (GBM-like) in Col-0 and its dependency on DDM1, MET1, and DRM2. **Figure S4.** Stable CG and nonCG DNA methylation level in *ddm1* plants during defense response under sub-optimal priming condition. **Figure S5.** Removal of gene body methylation enhances inducible activation of genes. **Figure S6.** Characteristics of strongly demethylated GBM in *ddm1* (GBM^*ddm1*^), unconventional GBM cluster genes (GBM^uc^) and conventional GBM cluster genes (GBM^C^). **Figure S7.** Interaction network associated with *ddm1 *hyperactivated genes during defense response under suboptimal priming condition. **Figure S8.** RT-qPCR confirmation of gene expression change from microarray data. **Figure S9.** *WRKY* genes expression in Col-0 and *cpk4* after DC inoculation to mock (mockDC) and BABA primed (BAoptDC) plants. **Figure S10.** non-hub-like genes did not affect primed defense response. **Figure S11.** Characteristics of GBM genes with interchangeable or stable methylation in natural variants. **Figure S12.** Histone modification patterns around novel priming regulators. **Figure S13.** The contribution of gene body CG-hypomethylation to transcriptional potentiation during weakly-primed and fully primed defense response.

**Additional file 2: Supplementary data 1.** Number of interrogated genes and DNA methylation regions for the analyses in Figs. 2 to 6 and Additional file 1: Fig. S6.

**Additional file 3: Supplementary data 2.** Selected 133 hub-like genes for subnetwork model in Additional file 1: Fig. S7.

**Additional file 4: Table S1.** Primers used for RT-qPCR and ChIP-qPCR.

**Additional file 5.** Review history.

## Data Availability

The bisulfite sequencing, histone H1, H2A.Z ChIP sequencing, and microarray data generated from the Col-0 and *ddm1* plants under the mock, BAsub, mockDC, and BAsubDC conditions were deposited in the NCBI Short Read Archive for bisulfite sequencing data (accession ID: GSE98162) [101], for ChIP sequencing data (accession ID: PRJNA915195) [102], and NCBI Gene Expression Omnibus for microarray data (accession ID: GSE59914) [103]. RNA sequencing (RNA-seq) data for avrRpt2, avrRpm1, and DC inoculation [40], *h1*, *met1*, and *h1met1* RNA-seq data, histone H1 and H3K9me2 data [15, 51], DNA methylation data for *ddm1*, *h1ddm1*, and *drm2* [10, 15], DNA methylation data for *drm1 drm2 cmt2 cmt3* and *met1* plants [11, 52], the average gene-body DNA methylation level (CG, CHG, and CHH) of 927 Arabidopsis natural population [64, 68] were downloaded from GEO (GSE88798 [104], GSE122394 [105], GSE41302 [106], GSE96994 [107], GSE51304 [108], GSE39901 [109], GSE43857 [110]). Histone variants and modified histone H3 data were obtained from GEO and ArrayExpress [54, 58, 111–115] (H2A.Z, H3K4me1, H3K27me3, H3K36me3, and H3K56ac (GSE128434 [116]), H2A.W (GSE150436 [117]), H3.1 and H3.3 (GSE34840 [118]), H3K4me3 (E-MTAB-5048 [119]), H3K9ac and H3K27ac (GSE79524 [120]), H3K9me1, H3K23ac (GSE51304 [108]), H3K27me1 (GSE111811 [121]), H3K36me2 (GSE28398 [122])). DNA accessibility data for Col-0 and *ddm1* were downloaded from GEO [123] (GSE155503 [124]). The gene responsiveness score and gene expression variation data (normalized CV^2^) were retrieved from Aceituno et al. [69] and Cortijo et al. [60]. H2A.Z ChIP-chip data were retrieved from Zilberman et al. [33] (GSE12212 [125]). No custom scripts and software was used other than those mentioned in the “Methods” section.
