# Supplementary material for: DDM1-mediated gene body DNA methylation is associated with inducible activation of defense-related genes in Arabidopsis
Source: Genome Biol. 2023 May 5;24:106. doi: 10.1186/s13059-023-02952-7 (PMC10161647; doi:10.1186/s13059-023-02952-7)
Supplement: Supplementary file 1 — Additional file 1: Figure S1. Weakly primed defense response in ddm1 and met1 plants. Figure S2. Exploring gene expression change during weakly primed defense response in ddm1 (vs. Col-0). Figure S3. Transposon-like methylationand (TEM-like) and gene body-like methylation (GBM-like) in Col-0 and its dependency on DDM1, MET1, and DRM2. Figure S4. Stable CG and nonCG DNA methylation level in ddm1 plants during defense response under sub-optimal priming condition. Figure S5. Removal of gene body methylation enhances inducible activation of genes. Figure S6. Characteristics of strongly demethylated GBM in ddm1 (GBMddm1), unconventional GBM cluster genes (GBMuc) and conventional GBM cluster genes (GBMC). Figure S7. Interaction network associated with ddm1 hyperactivated genes during defense response under suboptimal priming condition. Figure S8. RT-qPCR confirmation of gene expression change from microarray data. Figure S9. WRKY genes expression in Col-0 and cpk4 after DC inoculation to mock (mockDC) and BABA primed (BAoptDC) plants. Figure S10. non-hub-like genes did not affect primed defense response. Figure S11. Characteristics of GBM genes with interchangeable or stable methylation in natural variants. Figure S12. Histone modification patterns around novel priming regulators. Figure S13. The contribution of gene body CG-hypomethylation to transcriptional potentiation during weakly-primed and fully primed defense response. [file 13059_2023_2952_MOESM1_ESM.pdf]

Fig S1

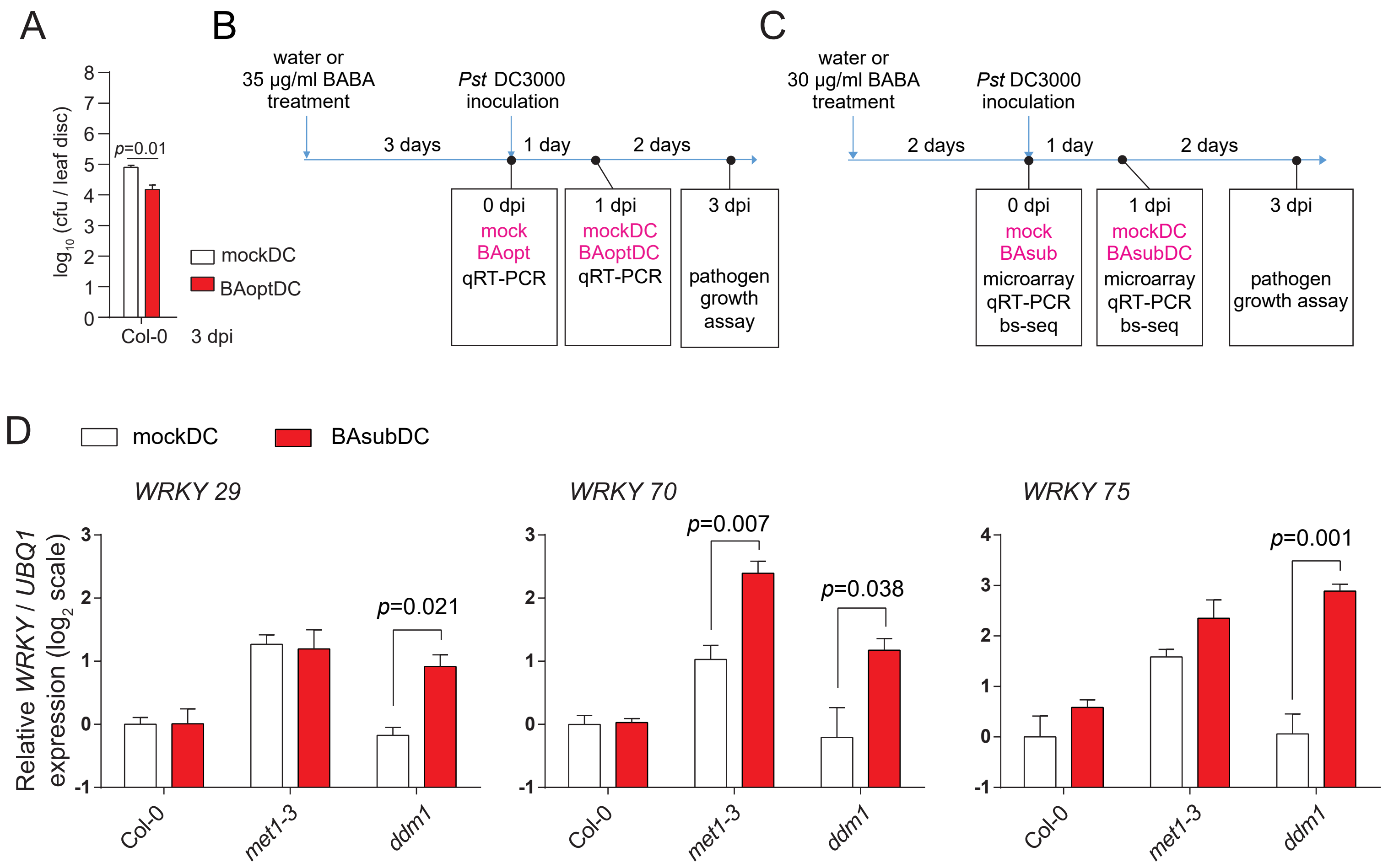


**Figure S1**. Weakly primed defense response in *ddm1* and *met1* plants.

**(A)** Bacterial growth test under optimal priming condition. Experiments were performed as described in the legend of Figure 1 except that a higher concentration of BABA (BAopt; 10 ml of 35 µg/ml per plant) was applied three days before DC inoculation to fully prime plants. *p*-value by Student's *t*-test; Error bars indicate SEM (n = 10). Data from Figure 7A. cfu, colony forming unit.

**(B)** Experimental design to investigate the defense response under optimal priming conditions.

**(C)** Experimental design to investigate the defense response under sub-optimal priming conditions.

**(D)** Expression of *WRKY* genes in Col-0, *ddm1*, and *met1* plants one day post *Pseudomonas syringae pv. tomato* DC3000 inoculation with (BAsubDC) or without (mockDC) sub-optimal concentration of BABA treatment. Error bars indicate SEM (n ≧ 3). *p*-values by Student's *t*-test. *UBQ1* expression was used as an internal control. Expression levels for each gene were normalized by that of an internal control *UBQ1*, which were further normalized with respect to those in Col-0 under mockDC. $Log_2$(normalized expression levels) are presented.

Fig S2

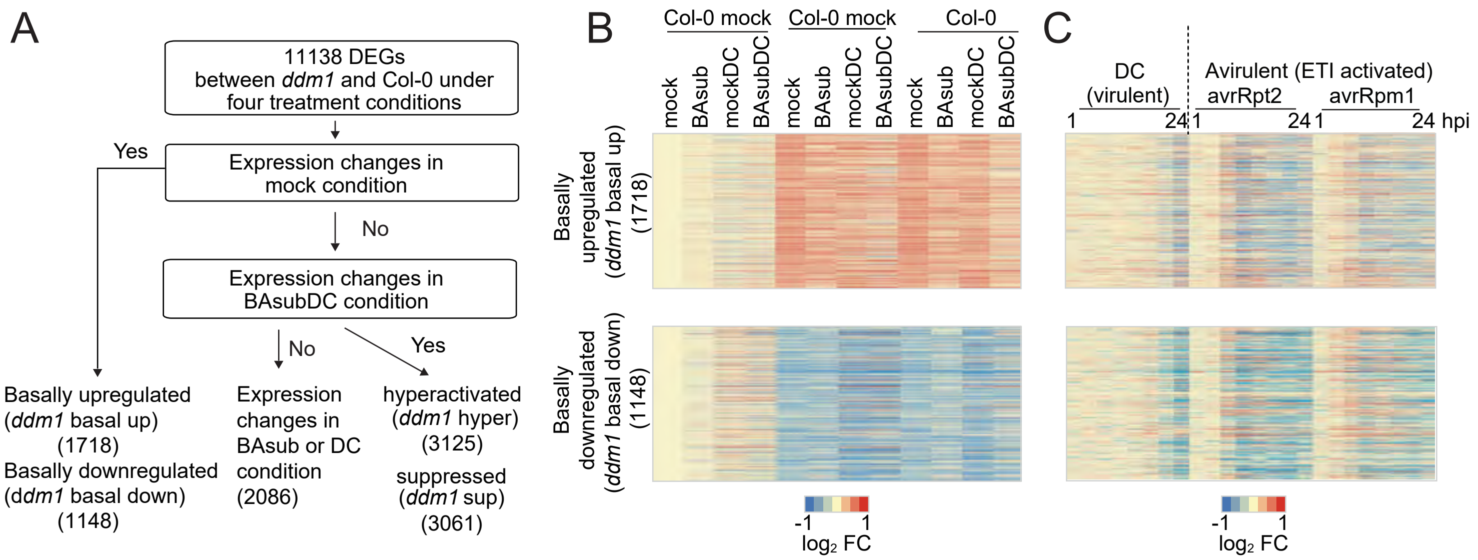


## D

| GO-Slim Biological Process (*ddm1* basal up) | Fold enrichment | FDR ($-\log_{10}$) |
|---|---|---|
| cellular modified amino acid metabolic process | 3.1 | 2.2 |
| sulfur compound metabolic process | 2.99 | 4.9 |
| alpha-amino acid metabolic process | 2.44 | 2.2 |
| microtubule-based process | 2.3 | 2.2 |
| anatomical structure development | 2.27 | 2.2 |
| cellular amino acid metabolic process | 2.16 | 2.6 |
| carboxylic acid metabolic process | 2.16 | 5.2 |
| organic acid metabolic process | 2.13 | 5.2 |
| oxoacid metabolic process | 2.13 | 5.3 |
| developmental process | 2.11 | 2.2 |

| GO-Slim Biological Process (*ddm1* hyper) | Fold enrichment | FDR ($-\log_{10}$) |
|---|---|---|
| **defense response to fungus** | **9.25** | **2.2** |
| endoplasmic reticulum unfolded protein response | 6.17 | 3.0 |
| glutathione metabolic process | 4.01 | 2.6 |
| carbohydrate derivative catabolic process | 3.59 | 2.8 |
| response to endoplasmic reticulum stress | 3.47 | 3.1 |
| aerobic respiration | 3.38 | 2.8 |
| vacuolar transport | 3.28 | 2.7 |
| regulation of signal transduction | 3.26 | 2.2 |
| lipid modification | 3.25 | 2.3 |
| **response to external biotic stimulus** | **3.21** | **4.2** |
| **defense response** | **2.95** | **4.7** |

| GO-Slim Biological Process (*ddm1* basal down) | Fold enrichment | FDR ($-\log_{10}$) |
|---|---|---|
| ribosomal small subunit biogenesis | 3.93 | 3.0 |
| organelle assembly | 3.33 | 4.7 |
| ribonucleoprotein complex assembly | 2.92 | 2.6 |
| ribosome biogenesis | 2.54 | 3.8 |
| peptide metabolic process | 2.08 | 2.5 |
| protein-containing complex assembly | 2.08 | 2.1 |

| GO-Slim Biological Process (*ddm1* sup) | Fold enrichment | FDR ($-\log_{10}$) |
|---|---|---|
| photosynthesis | 6.43 | 4.5 |

**Figure S2.** Exploring gene expression change during weakly primed defense response in *ddm1* (vs. Col-0).

**(A)** Categorization of differentially expressed genes in *ddm1* (vs. Col-0) during weakly primed defense response.

**(B)** Heatmaps of gene expression (basally up- or downregulated in *ddm1*) in Col-0 and *ddm1* plants before (0 dpi; mock and BAsub) and after (1 dpi; mockDC and BAsubDC) DC inoculation. For Col-0 and *ddm1* columns, $\log_2$ fold change (FC) against Col-0 mock samples were plotted. For *ddm1*/Col-0 columns, $\log_2$ fold change in *ddm1* plants against Col-0 under four different conditions were plotted.

**(C)** Heatmaps of gene expression changes after inoculation of virulent (DC) or avirulent (avrRpt2 and avrRpm1) pathogens. $\mathrm{Log}_2$ fold change (FC) against mock control at each time point was plotted.

**(D)** Gene ontology terms of four groups categorized in Figure S2A. *ddm1* hyper genes were enriched with the function related to defense response.

Fig S3

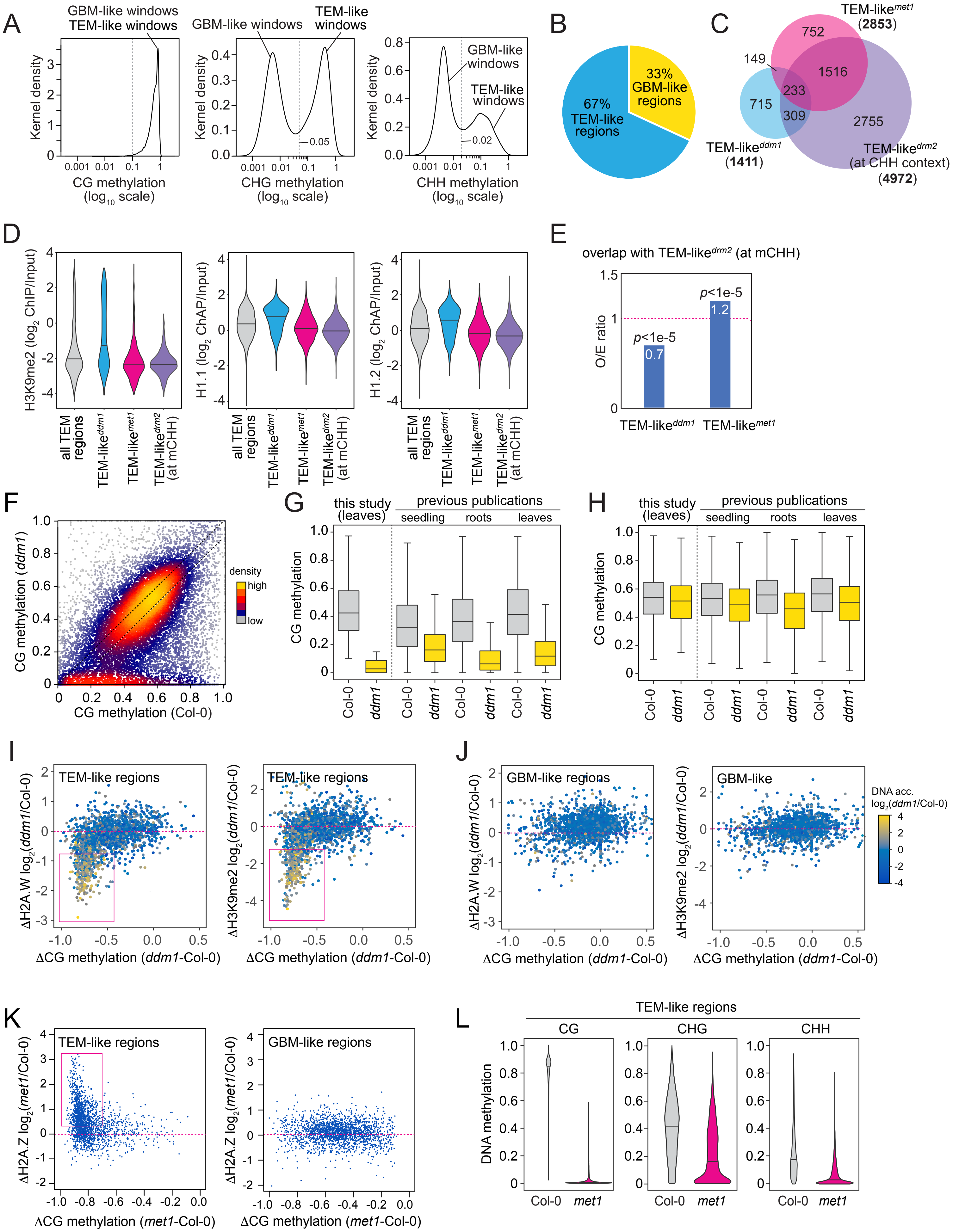

A
GBM-like windows
TEM-like windows
Kernel density
CG methylation (log10 scale)
GBM-like windows
TEM-like windows
CHG methylation (log10 scale)
0.05
GBM-like windows
TEM-like windows
CHH methylation (log10 scale)
0.02
B
67% TEM-like regions
33% GBM-like regions
C
TEM-like met1 (2853)
752
149
1516
233
715
309
2755
TEM-like ddm1 (1411)
TEM-like drm2 (at CHH context) (4972)
D
H3K9me2 (log2 ChIP/Input)
H1.1 (log2 ChAP/Input)
H1.2 (log2 ChAP/Input)
all TEM regions
TEM-like ddm1
TEM-like met1
TEM-like drm2 (at mCHH)
E
overlap with TEM-like drm2 (at mCHH)
O/E ratio
p<1e-5
0.7
p<1e-5
1.2
TEM-like ddm1
TEM-like met1
F
CG methylation (ddm1)
CG methylation (Col-0)
density
high
low
G
this study (leaves)
previous publications
seedling
roots
leaves
CG methylation
Col-0
ddm1
H
this study (leaves)
previous publications
seedling
roots
leaves
CG methylation
Col-0
ddm1
I
TEM-like regions
ΔH2A.W log2(ddm1/Col-0)
ΔCG methylation (ddm1-Col-0)
TEM-like regions
ΔH3K9me2 log2(ddm1/Col-0)
ΔCG methylation (ddm1-Col-0)
J
GBM-like regions
ΔH2A.W log2(ddm1/Col-0)
ΔCG methylation (ddm1-Col-0)
GBM-like
ΔH3K9me2 log2(ddm1/Col-0)
ΔCG methylation (ddm1-Col-0)
DNA acc. log2(ddm1/Col-0)
K
TEM-like regions
ΔH2A.Z log2(met1/Col-0)
ΔCG methylation (met1-Col-0)
GBM-like regions
ΔH2A.Z log2(met1/Col-0)
ΔCG methylation (met1-Col-0)
L
TEM-like regions
CG
CHG
CHH
DNA methylation
Col-0
met1

**Figure S3.** Transposon-like methylation (TEM-like) and gene body-like methylation (GBM-like) in Col-0 and its dependency on DDM1, MET1, and DRM2.

**(A)** Kernel density plots of CG, CHG, and CHH DNA methylation at TEM-like and GBM-like windows. Dotted lines indicate methylation threshold to distinguish TEM-like and GBM-like. Windows with no methylation were removed from the analysis.

**(B)** The proportion of TEM-like and GBM-like regions in Col-0 (ratio calculated by the total length of TEM-like regions and GBM-like regions).

**(C)** A Venn diagram of demethylated TEM-like in *met1* (TEM-like$^{met1}$), *ddm1* plants (TEM-like$^{ddm1}$), and *drm2* plants (TEM-like$^{drm2}$, at CHH context).

**(D)** Violin plots of indicated chromatin feature levels in Col-0 at all TEM-like and demethylated TEM-like in the mutants.

**(E)** Overlap between TEM-like$^{drm2}$ and TEM-like$^{ddm1}$ or TEM-like$^{met1}$ plants (*p*-values by Fisher's exact test).

**(F)** Scatter plot of CG methylation level of GBM-like regions in Col-0 (x-axis) and *ddm1* (y-axis) plants. Each dot represents a gene body methylation locus. Dot colors indicate density of dots.

**(G)** CG methylation level of strongly demethylated GBM-like in *ddm1* (GBM-like$^{ddm1}$ in Figure 3C) in this study and other *ddm1* data.

**(H)** CG methylation level of GBM-like that their methylation levels are weakly affected or not affected in *ddm1* (GBM-like$^{weak}$ in Figure 3C) in this study and publicly available data.

**(G-H)** Seedling and root DNA methylation data are from Zemach et al. [10]. Leaf DNA methylation data are from Lyons et al. [15].

**(I)** Scatter plots of CG methylation change at TEM-like in relation to H2A.W and H3K9me2 change in *ddm1*. Magenta box indicates highly demethylated TEM-like regions with increased DNA accessibility in *ddm1*.

**(J)** Scatter plot of CG methylation change at GBM-like in relation to H2A.W and H3K9me2 change in *ddm1.*

**(K)** Scatter plot of CG methylation change at TEM-like and GBM-like in relation to H2A.Z change in *met1*. Magenta box indicates highly demethylated TEM-like regions with increased histone H2A.Z levels in *met1*. DNA methylation data and H2A.Z data are from Stroud et al. [52] and Zilberman et al. [33]

**(L)** Violin plots of DNA methylation levels of TEM-like regions in Col-0 and *met1* plants. DNA methylation data are from Stroud et al. [52].

Fig S4

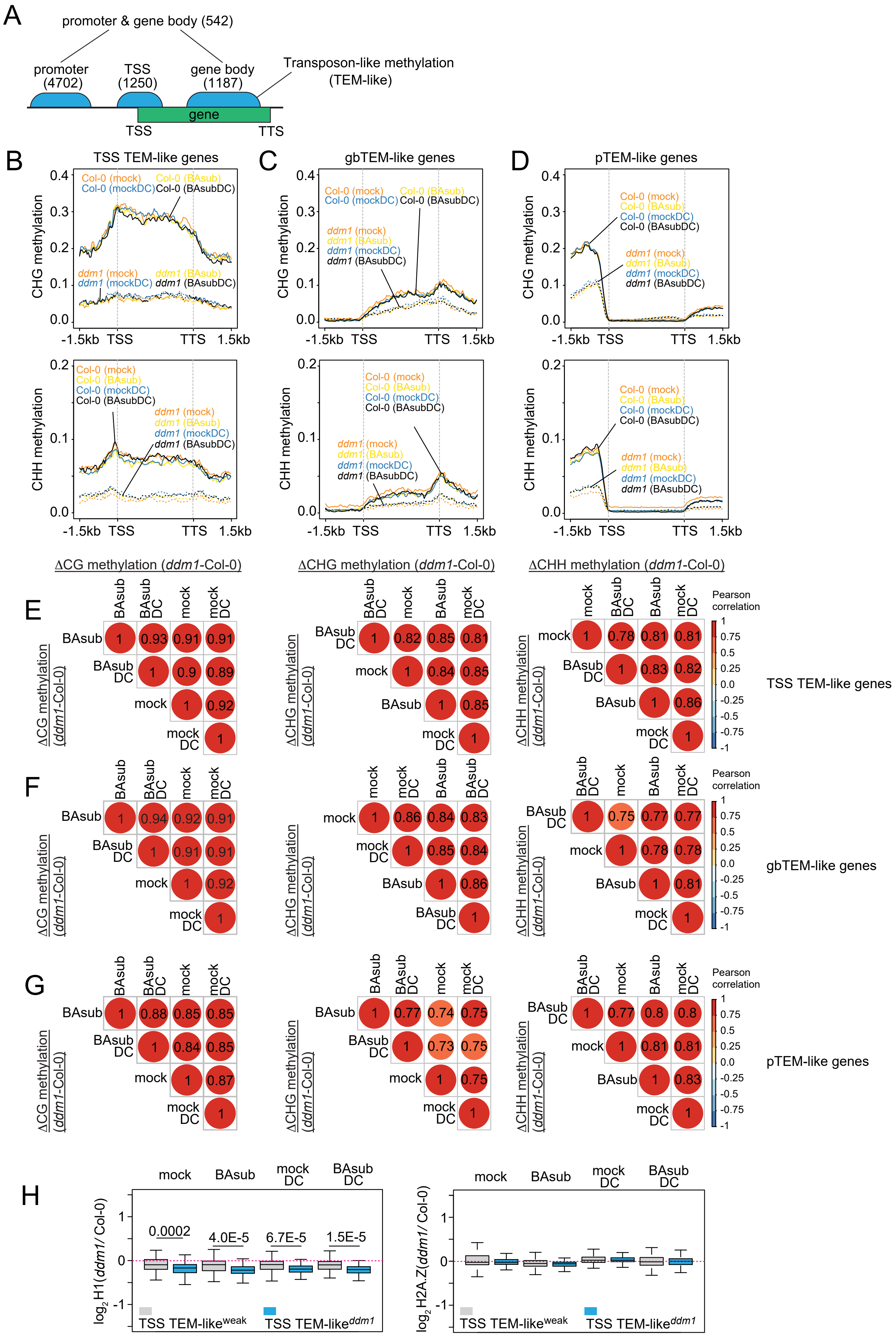
A
promoter & gene body (542)
promoter (4702)
TSS (1250)
gene body (1187)
Transposon-like methylation (TEM-like)
gene
TSS
TTS
B
TSS TEM-like genes
C
gbTEM-like genes
D
pTEM-like genes
CHG methylation
CHH methylation
Col-0 (mock)
Col-0 (BAsub)
Col-0 (mockDC)
Col-0 (BAsubDC)
ddm1 (mock)
ddm1 (BAsub)
ddm1 (mockDC)
ddm1 (BAsubDC)
-1.5kb
TSS
TTS
1.5kb
ΔCG methylation (ddm1-Col-0)
ΔCHG methylation (ddm1-Col-0)
ΔCHH methylation (ddm1-Col-0)
E
F
G
Pearson correlation
TSS TEM-like genes
gbTEM-like genes
pTEM-like genes
H
mock
BAsub
mock DC
BAsub DC
0.0002
4.0E-5
6.7E-5
1.5E-5
log2 H1(ddm1/ Col-0)
log2 H2A.Z(ddm1/ Col-0)
TSS TEM-like^weak
TSS TEM-like^ddm1

**Figure S4.** Stable CG and nonCG DNA methylation level in *ddm1* plants during defense response under sub-optimal priming condition.

**(A)** Number of Araport11 gene annotations with TEM-like. Promoter TEM-like; TEM-like located at 0 to 1.5 kb upstream of genes, TSS TEM-like; TEM-like overlap with TSS, gene body TEM-like; TEM-like overlap with gene body but do not overlap with TSS. TTS, transcription termination sites.

**(B-D)** Averaged CHG and CHH DNA methylation level around genes that have TEM-like at TSS (B), gene bodies (C), or promoters (D). TSS, transcription start sites. TTS, transcription termination sites.

**(E-G)** Pairwise Pearson's correlation coefficients of CG and non-CG methylation change (*ddm1* - Col-0) at indicated conditions during defense response with sub-optimal priming. Pairwise Pearson's correlation coefficients were plotted by genes with TEM-like at TSS (E), gene body (F), and promoters (G). TSS, transcription start sites.

**(H)** Boxplots of histone H1 (left) and H2A.Z (right) levels of genes with TSS TEM-like$^{weak}$ and TSS TEM-like$^{\textit{ddm1}}$ in each treatment conditions.

# Fig S5

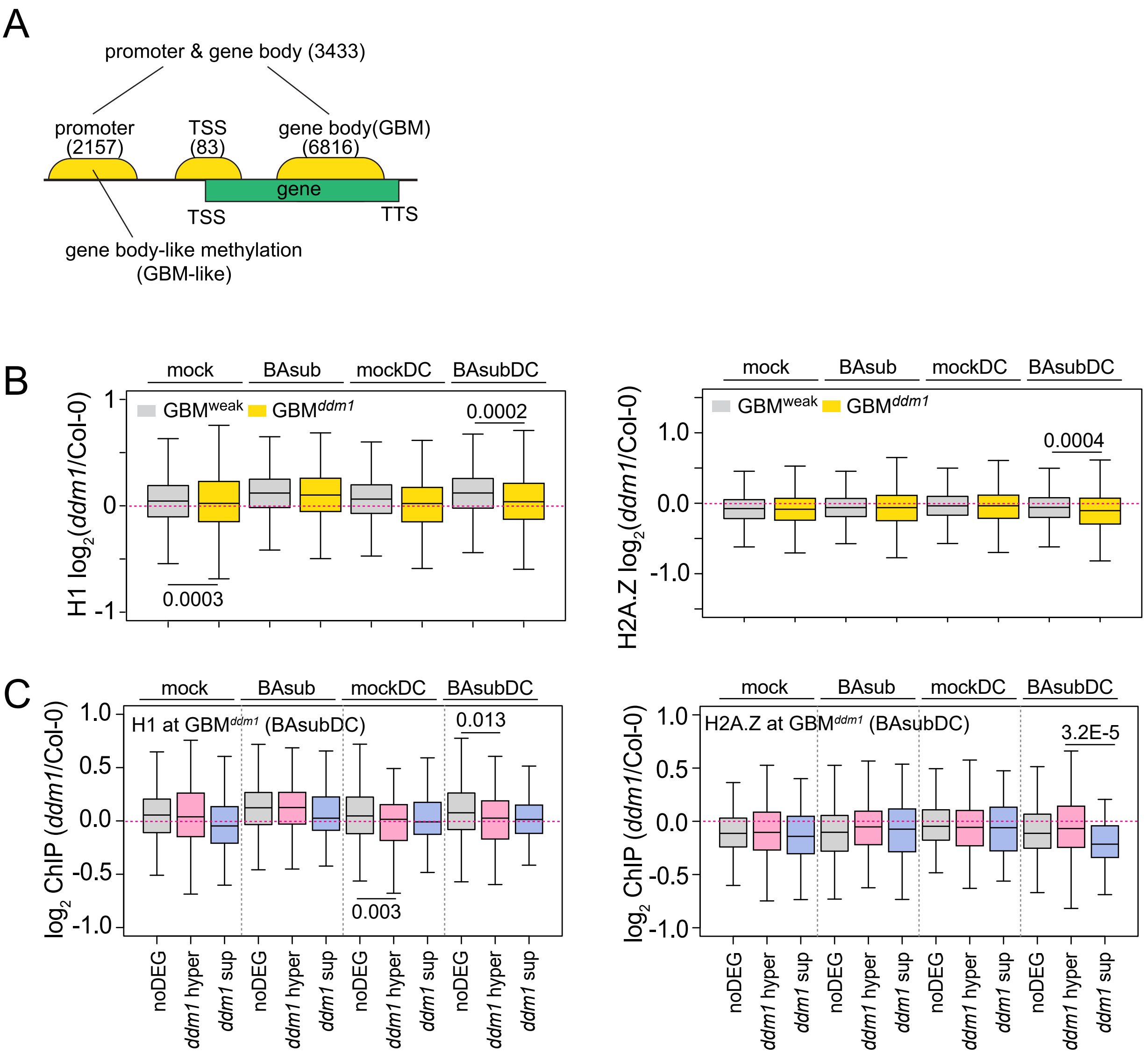


## D

**Enriched GO terms in GBM$^{weak}$ genes (BAsubDC condition)**

| GO-Slim Biological Process | Fold enrichment | FDR ($-log_{10}$) |
|---|---|---|
| protein palmitoylation | 4.02 | 2.7 |
| peroxisome organization | 3.69 | 2.6 |
| movement of cell or subcellular component | 3.05 | 3.2 |
| cytokinesis | 2.98 | 2.1 |
| cell division | 2.76 | 2.3 |
| lipoprotein biosynthetic process | 2.73 | 2.4 |
| protein deubiquitination | 2.63 | 2.3 |

| GO-Slim Biological Process | Fold enrichment | FDR ($-log_{10}$) |
|---|---|---|
| glycoprotein metabolic process | 2.62 | 2.0 |
| protein acylation | 2.59 | 2.4 |
| protein autophosphorylation | 2.58 | 3.6 |
| plant-type cell wall organization or biogenesis | 2.44 | 2.1 |
| endocytosis | 2.44 | 2.1 |
| monocarboxylic acid metabolic process | 2.1 | 3.4 |

**Figure S5.** Removal of gene body methylation enhances inducible activation of genes.

**(A)** Number of Araport11 gene annotations with gene body-like methylation (GBM-like). Promoter GBM-like; GBM-like located at 0 to 1.5 kb upstream of genes, TSS GBM-like; GBM-like overlap with TSS, gene body GBM-like (GBM); GBM-like overlap with gene body but do not overlap with TSS, conventionally GBM. TTS, transcription termination sites.

**(B)** Boxplots of histone H1 (left) and H2A.Z (right) levels of genes with $GBM^{weak}$ and $GBM^{ddm1}$ in each treatment conditions.

**(C)** Boxplots of histone H1 (left) and H2A.Z (right) levels of $GBM^{ddm1}$ genes (*ddm1* vs. Col-0, BAsubDC) overlap with DEGs (*ddm1* hyper, *ddm1* sup) and $GBM^{ddm1}$ genes that do not overlap with DEGs (noDEG) in each treatment conditions.

**(D)** Enriched gene ontology terms among genes with GBM that their methylation levels are weakly affected or not affected in *ddm1* ($GBM^{weak}$) under BAsubDC condition.

Fig S6

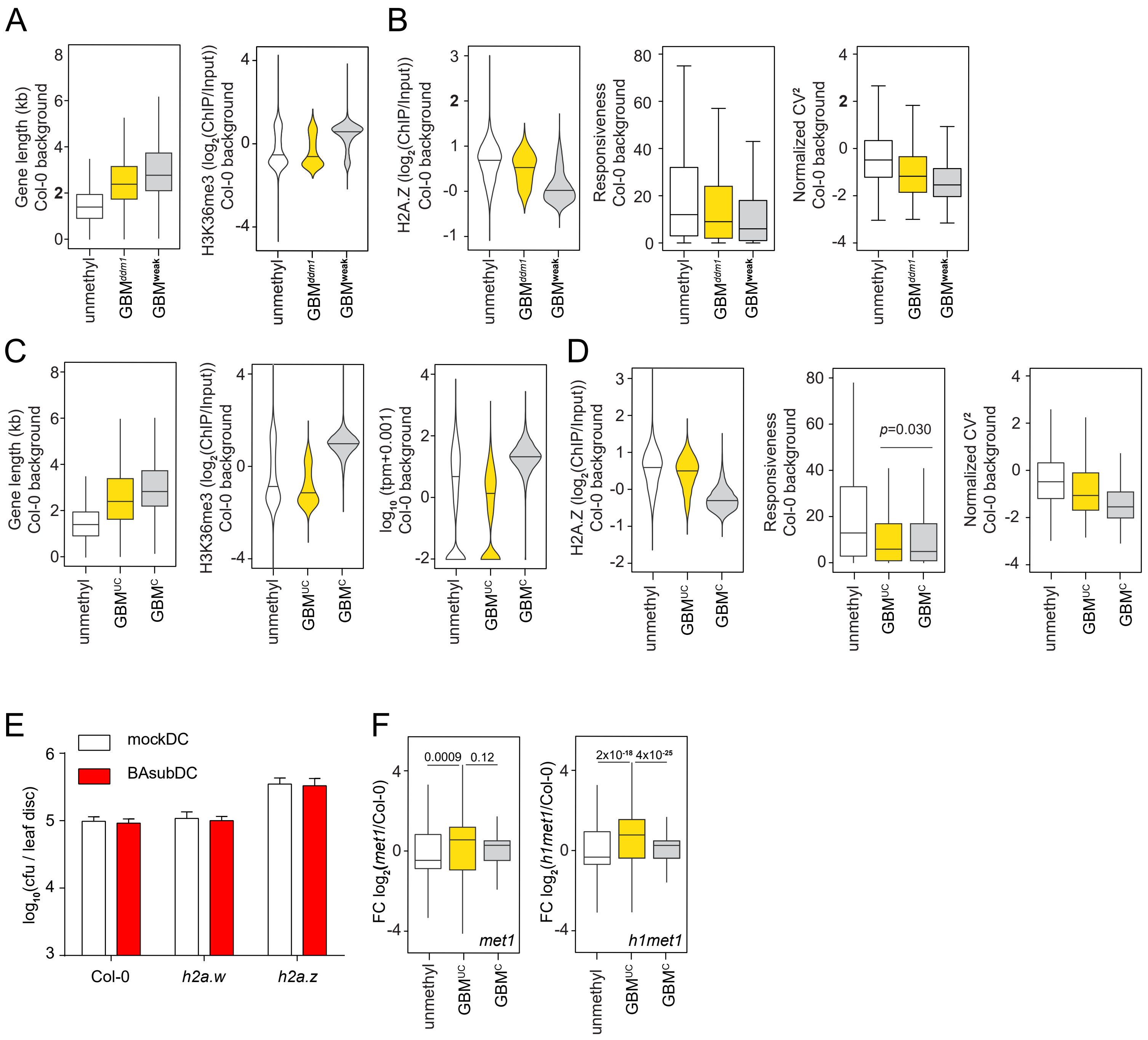
A
B
C
D
E
F
Gene length (kb) Col-0 background
H3K36me3 (log2(ChIP/Input)) Col-0 background
H2A.Z (log2(ChIP/Input)) Col-0 background
Responsiveness Col-0 background
Normalized CV² Col-0 background
log10 (tpm+0.001) Col-0 background
unmethyl
GBMddm1
GBMweak
GBMUC
GBMC
p=0.030
mockDC
BAsubDC
log10(cfu / leaf disc)
Col-0
h2a.w
h2a.z
FC log2(met1/Col-0)
FC log2(h1met1/Col-0)
0.0009
0.12
2x10-18
4x10-25
met1
h1met1

**Figure S6.** Characteristics of strongly demethylated GBM in *ddm1* ($GBM^{ddm1}$), unconventional GBM cluster genes ($GBM^{UC}$) and conventional GBM cluster genes ($GBM^{C}$).

**(A)** A boxplot and a violin plot of gene length and H3K36me3 level of unmethylated genes in both Col-0 and *ddm1* (unmethyl), genes with strongly demethylated GBM in *ddm1* ($GBM^{ddm1}$), and genes with GBM that their methylation levels are weakly affected or not affected in *ddm1* ($GBM^{weak}$).

**(B)** A violin plot of H2A.Z level and boxplots of responsiveness and normalized $CV^2$ of unmethylated genes in both Col-0 and *ddm1* (unmethyl), genes with strongly demethylated GBM in *ddm1* ($GBM^{ddm1}$), and genes with GBM that their methylation levels are weakly affected or not affected in *ddm1* ($GBM^{weak}$). Responsiveness score and normalized $CV^2$ were calculated as Aceituno et al. [69] and Cortijo et al. [60].

**(C)** A boxplot and violin plots of gene length, H3K36me3 level, and expression level of unmethylated genes in both Col-0 and *ddm1* (unmethyl), unconventional GBM cluster genes ($GBM^{UC}$), and conventional GBM cluster genes ($GBM^{C}$). tpm indicates transcripts per million.

**(D)** A violin plot and boxplots of H2A.Z level, responsiveness and normalized $CV^2$ unmethylated genes in both Col-0 and *ddm1* (unmethyl), unconventional GBM cluster genes ($GBM^{UC}$), and conventional GBM cluster genes ($GBM^{C}$). Responsiveness score and normalized $CV^2$ were calculated as Aceituno et al. [69] and Cortijo et al. [60]. *p*-values by Student's *t*-test.

**(E)** Bacterial growth of *Pseudomonas syringae pv. tomato* DC3000 (DC) in H2A variant mutants with or without sub-optimal priming. For sub-optimal priming, water (mock control) or *β*-amino butyric acid (BAsub; 10 ml of 30 μg/ml per plant) was applied near roots two days prior pathogen inoculation. Bacterial growth was measured three days post-inoculation (dpi) (n = 5). cfu, colony forming units.

**(F)** Boxplots of gene expression fold change (FC) of differentially expressed genes in *met1* (left) and *h1met1* (right) plants (compared to Col-0, *q*-value<0.05) overlapping with unmethylated genes in both Col-0 and *ddm1* (unmethyl), unconventional GBM cluster genes ($GBM^{UC}$), and conventional GBM cluster genes ($GBM^{C}$). Numbers indicate *p*-values by Student's *t*-test.

Fig S7

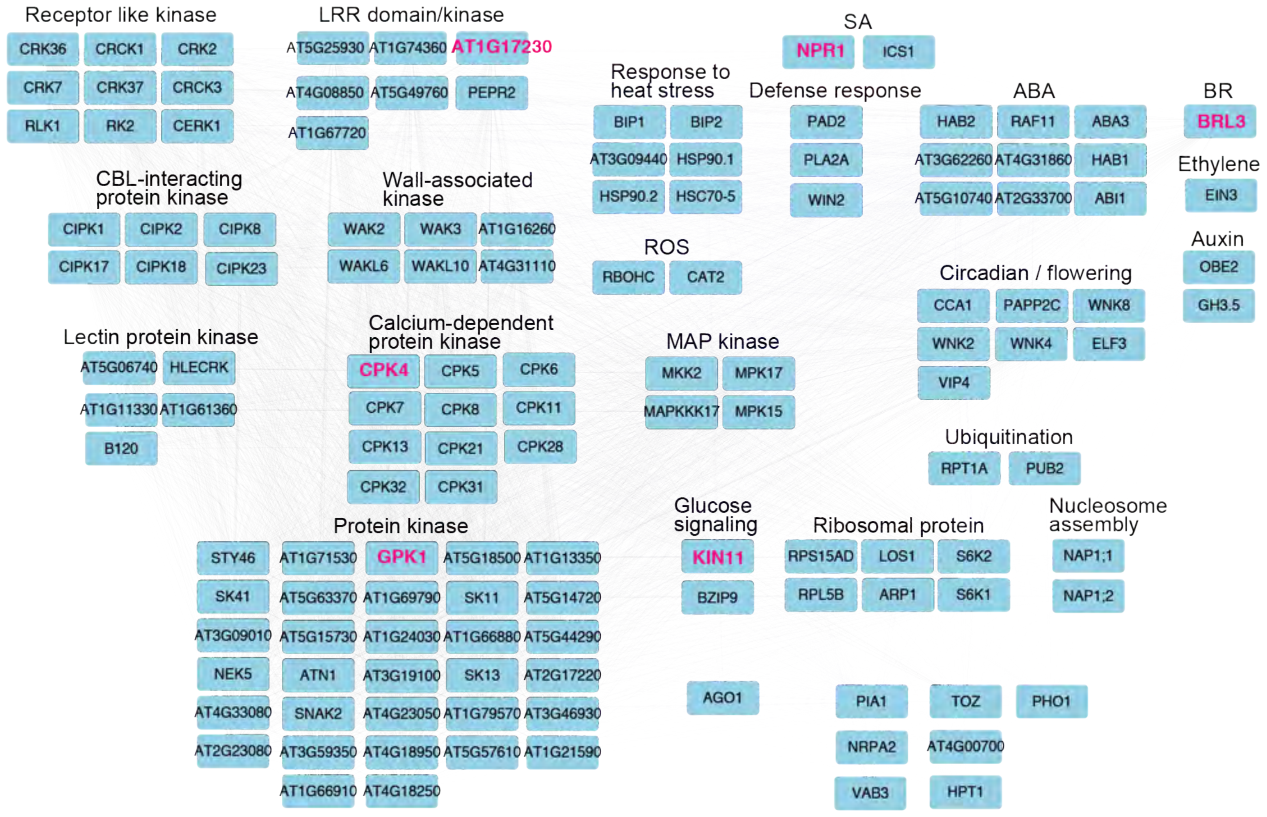


**Figure S7.** Interaction network associated with *ddm1* hyperactivated genes during defense response under suboptimal priming condition.

Gene-to-gene interactions were retrieved from iNID database [61]. SA, salicylic acid; ABA, abscisic acid; BR, brassinosteroids; ROS, reactive oxygen species. Nodes were arranged and grouped into functional modules based on their Gene Ontology molecular functions or biological processes terms. Associated GO terms were indicated above nodes.

Fig S8

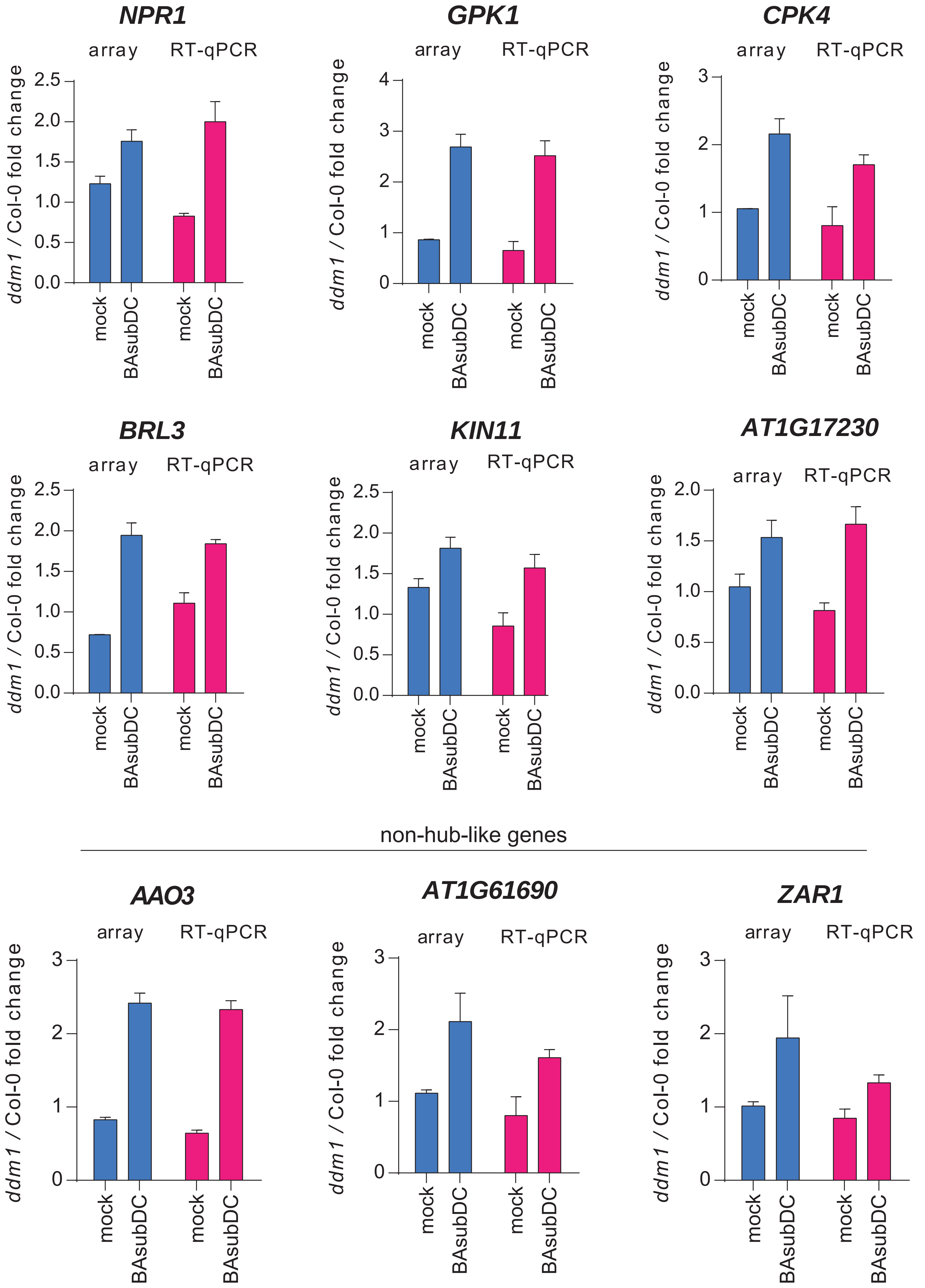


**Figure S8.** RT-qPCR confirmation of gene expression change from microarray data.

Gene expression fold-changes of the selected targets (from Figure S7) and non-hub-like genes in *ddm1* and Col-0 were plotted. *UBQ1* expression was used as an internal control. Error bars indicate SEM (n=3).

Fig S9

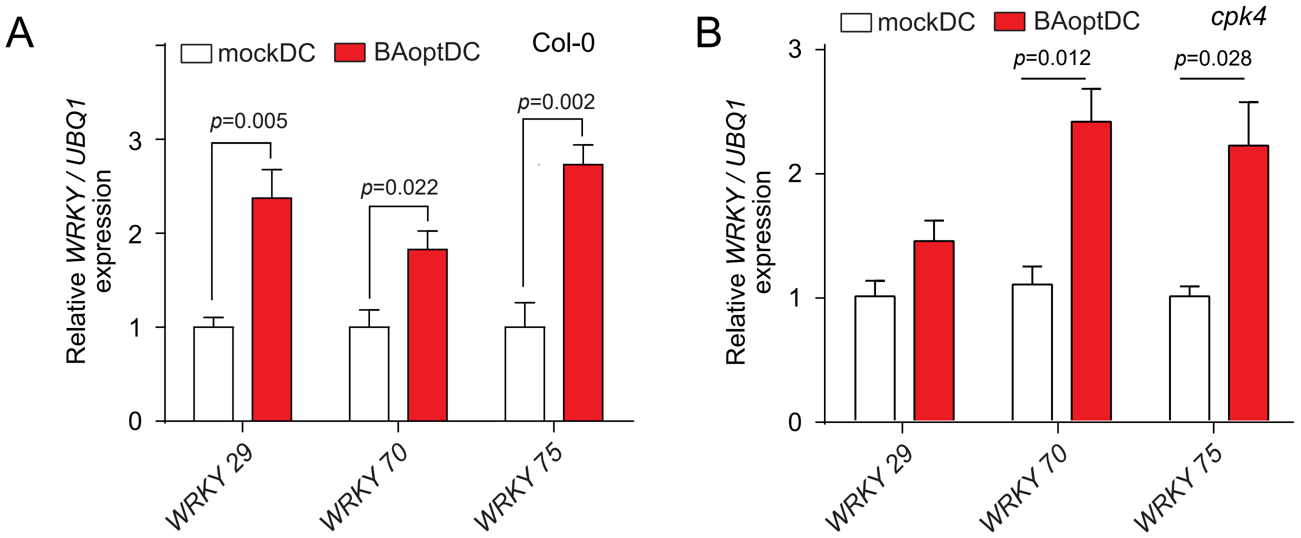


**Figure S9.** *WRKY* genes expression in Col-0 and *cpk4* after DC inoculation to mock (mockDC) and BABA primed (BAoptDC) plants.

**(A and B)** *WRKY* genes expression in Col-0 (A) and *cpk4* (B) was measured 1-day post-inoculation (dpi). *UBQ1* expression was used as an internal control. Error bars indicate SEM (n = 4 in A and n = 3 in B). Col-0 data from Figure 7C. *p*-values by Student's *t*-test.

Fig S10

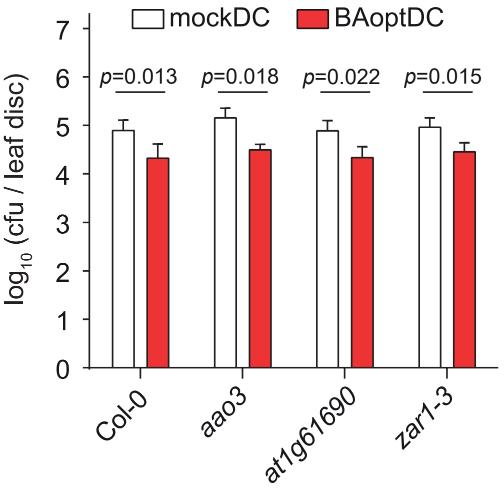


**Figure S10.** non-hub-like genes did not affect primed defense response.

Measuring the bacterial growth in three non-hub-like gene knockout plants was performed as described in the legend of Figure 1, except that a higher concentration of BABA (BAopt; 10 ml of 35 μg/ml per plant) was applied three days before DC inoculation to fully prime plants. *p*-value by Student's *t*-test. Error bars indicate SEM (n ≧ 5). cfu, colony forming units.

Fig S11

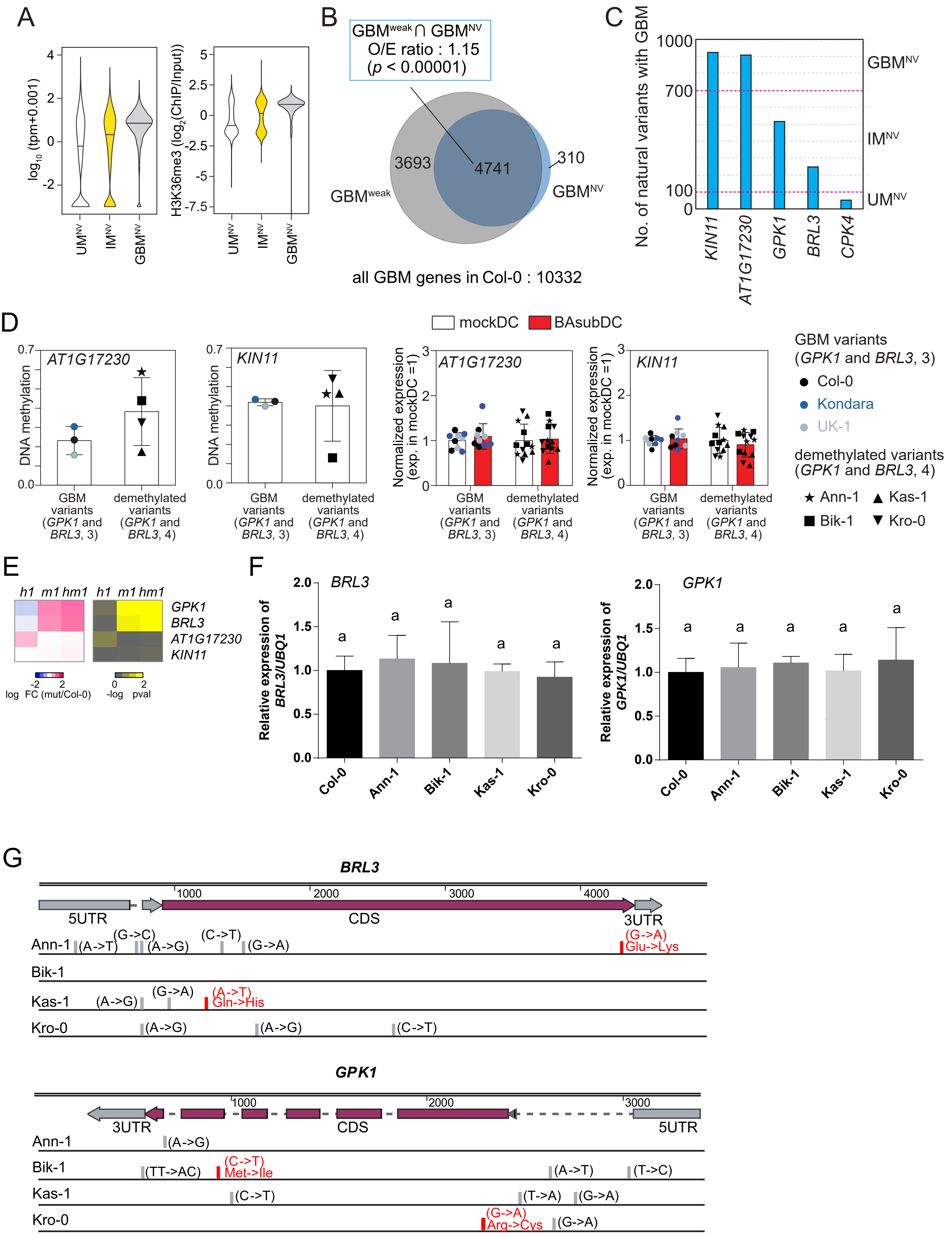

A
log10 (tpm+0.001)
H3K36me3 (log2(ChIP/Input))
UMNV
IMNV
GBMNV
B
GBMweak ∩ GBMNV
O/E ratio : 1.15
(p < 0.00001)
3693
4741
310
GBMweak
GBMNV
all GBM genes in Col-0 : 10332
C
No. of natural variants with GBM
GBMNV
IMNV
UMNV
KIN11
AT1G17230
GPK1
BRL3
CPK4
D
mockDC
BAsubDC
AT1G17230
KIN11
DNA methylation
Normalized expression (exp. in mockDC =1)
GBM variants (GPK1 and BRL3, 3)
demethylated variants (GPK1 and BRL3, 4)
Col-0
Kondara
UK-1
Ann-1
Kas-1
Bik-1
Kro-0
E
h1 m1 hm1
GPK1
BRL3
AT1G17230
KIN11
log FC (mut/Col-0)
-log pval
F
BRL3
GPK1
Relative expression of BRL3/UBQ1
Relative expression of GPK1/UBQ1
Col-0
Ann-1
Bik-1
Kas-1
Kro-0
G
BRL3
5UTR
CDS
3UTR
Ann-1 (A->T) (G->C) (A->G) (C->T) (G->A) (G->A) Glu->Lys
Bik-1
Kas-1 (A->G) (G->A) (A->T) Gln->His
Kro-0 (A->G) (A->G) (C->T)
GPK1
3UTR
CDS
5UTR
Ann-1 (A->G)
Bik-1 (TT->AC) (C->T) Met->Ile (A->T) (T->C)
Kas-1 (C->T) (T->A) (G->A)
Kro-0 (G->A) Arg->Cys (G->A)

**Figure S11.** Characteristics of GBM genes with interchangeable or stable methylation in natural variants.

**(A)** Violin plots of gene expression and H3K36me3 level at genes unmethylated in over 700 natural variants ($UM^{NV}$), genes with interchangeable GBM ($IM^{NV}$; possess GBM at 100-700 natural variants), and genes with stable GBM over 700 natural variants ($GBM^{NV}$). tpm indicates transcripts per million.

**(B)** Venn diagram of gene with GBM that their methylation levels are weakly affected or not affected in *ddm1* ($GBM^{weak}$) and genes with stable gene body DNA methylation in the natural population ($GBM^{NV}$) [64, 68]. *p*-value by Fisher's exact test.

**(C)** The number of individuals from the *Arabidopsis* natural populations that possess GBM at indicated genes was plotted [64, 68].

**(D)** Relative expression of novel priming regulators in natural variants. (Left) The average GBM level of *AT1G17230* and *KIN11* in GBM variants in *GPK1* and *BRL3* and demethylated variants in *GPK1* and *BRL3*. (Right) Expression of *AT1G17230 and KIN11* under mockDC condition was normalized to 1. Six natural variants and Col-0 were grouped by their GBM level at *GPK1* and *BRL3*. Col-0, Kondara, and UK-1 maintained GBM at both genes, and Ann-1, Bik-1, Kas-1, Kro-0 lost GBM at *GPK1* and *BRL3*. All natural variants maintained GBM at *AT1G17230* and *KIN11*. RT-qPCR data from three replicates per natural variants were averaged (n=9 for GBM variants, n=12 for demethylated variants). Error bars indicate standard deviation.

**(E)** Heatmaps of expression fold change (left; $log_2$(mutant/Col-0)) and *p*-values for differential expression (right) of novel priming regulators in *h1, met1* (*m1*), and *h1met1* (*hm1)* plants. RNA-seq data from Choi et al. 2020 [51].

**(F)** Gene expression of *BRL3* and *GPK1* in Col-0 and four naturally demethylated variants of *BRL3* and *GPK1* (Ann-1, Bik-1, Kas-1, and Kro-0). Gene expressions were measured in mock condition. *UBQ1* expression was used as an internal control. Different letters indicate significant differences at $p \leqq 0.05$ from one-way ANOVA with Tukey's correction ($\alpha = 0.05$). Error bars indicate standard deviation (n = 3).

**(G)** Polymorphisms of *BRL3* and *GPK1* in gene region among four naturally demethylated variants of *BRL3* and *GPK1* (Ann-1, Bik-1, Kas-1, and Kro-0). Red colors indicate non-synonymous mutation and altered amino acid. Polymorphism data were retrieved from 1001 Genomes Project (https://1001genomes.org/)

# Fig S12

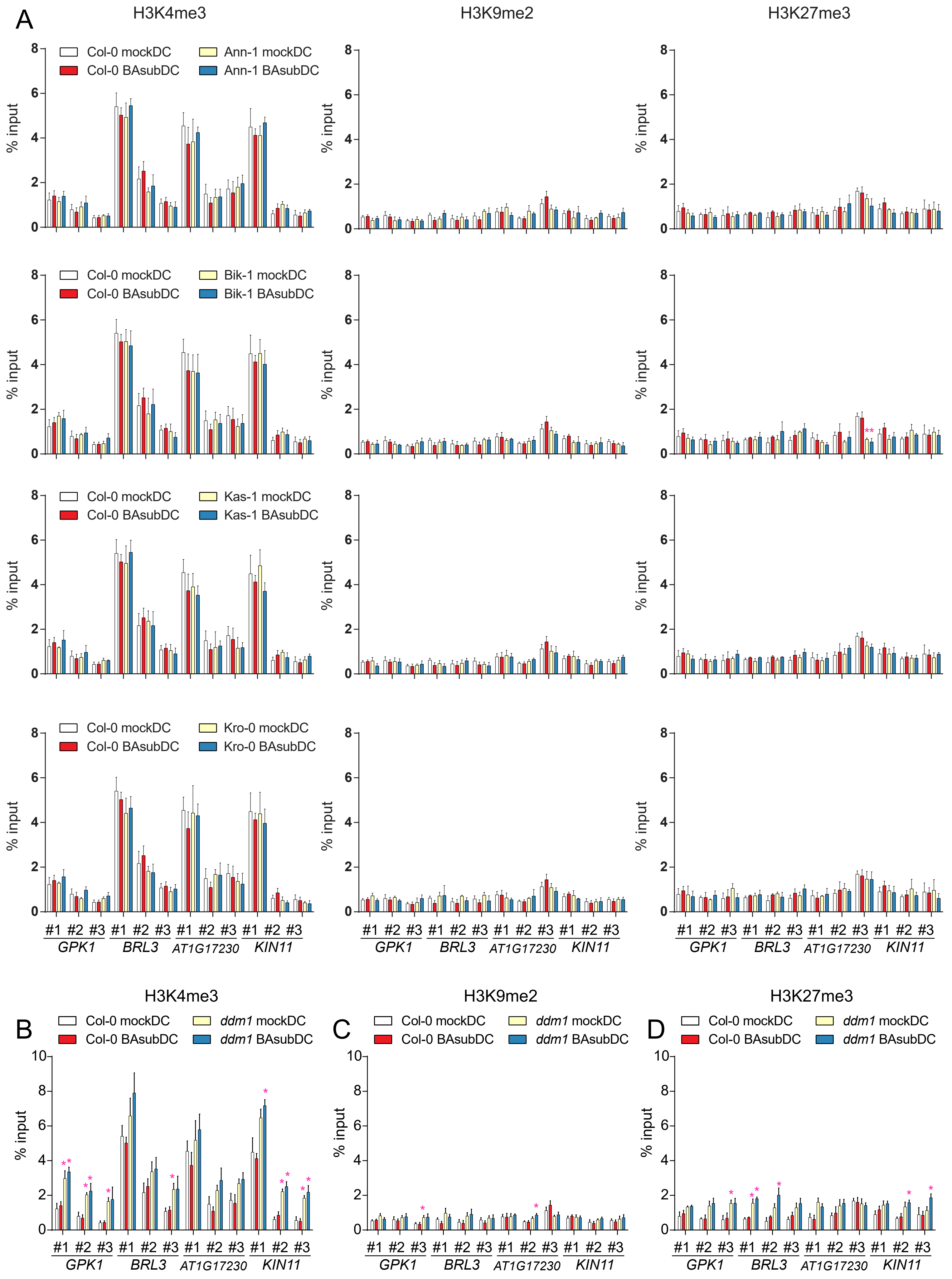
A
H3K4me3
H3K9me2
H3K27me3
Col-0 mockDC
Col-0 BAsubDC
Ann-1 mockDC
Ann-1 BAsubDC
Bik-1 mockDC
Bik-1 BAsubDC
Kas-1 mockDC
Kas-1 BAsubDC
Kro-0 mockDC
Kro-0 BAsubDC
% input
#1 #2 #3 #1 #2 #3 #1 #2 #3 #1 #2 #3
GPK1
BRL3
AT1G17230
KIN11
B
C
D
ddm1 mockDC
ddm1 BAsubDC

**Figure S12.** Histone modification patterns around novel priming regulators.

**(A)** Histone H3K4me3, H3K9me2, and H3K27me3 enrichment around novel priming regulators in mockDC and BAsubDC conditions in Col-0 and four natural variants (Ann-1, Bik-1, Kas-1, Kro-0) that both *GPK1* and *BRL3* were demethylated. #1-3 primers amplify TSS-proximal region, gene body region and TTS-proximal region, respectively. Histone enrichment data of Col-0 from Figure S12 (B, C, and D)

**(B-D)** Histone H3K4me3 (B), H3K9me2 (C), and H3K27me3 (D) enrichment around novel priming regulators in mockDC and BAsubDC condition in Col-0 and *ddm1* plants. #1-3 primers amplify TSS-proximal region, gene body region and TTS-proximal region, respectively.

**(A-D)** Asterisks indicate statistically significant change in enrichment ($p<0.05$, Col-0 mockDC vs. *ddm1* mockDC, Col-0 BAsubDC vs. *ddm1* BAsubDC, Col-0 mockDC vs. each natural variant mockDC, Col-0 BAsubDC vs. each natural variant BAsubDC). *p*-values by Student's *t*-test.

Fig S13

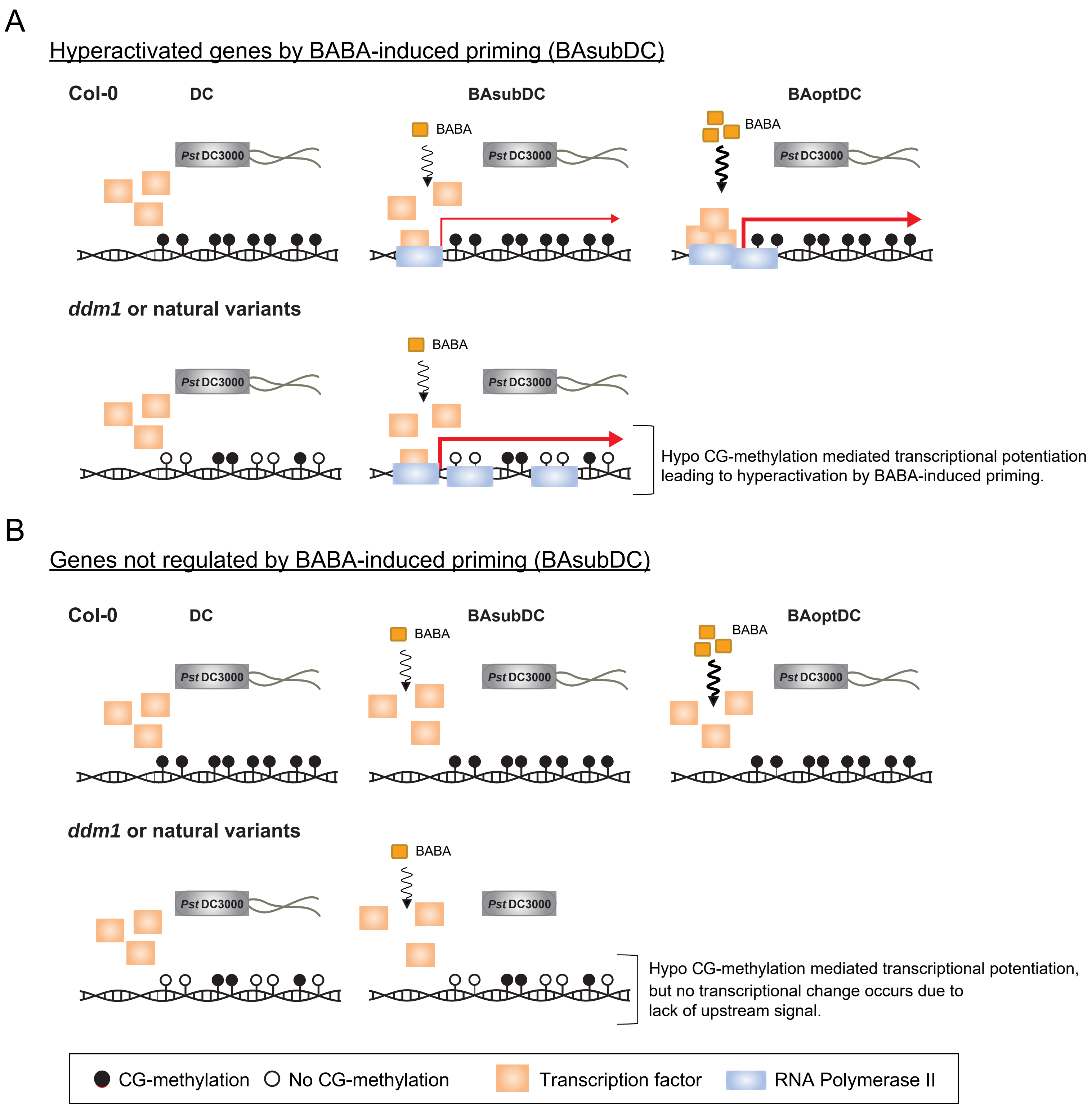


**Figure S13.** The contribution of gene body CG-hypomethylation to transcriptional potentiation during weakly-primed and fully primed defense response. (A) Demethylation of GBM$^{ddm1}$ increased transcriptional potential of priming regulators, leading to hyperactivation by weak defense priming (BAsubDC). (B) Demethylation of GBM$^{ddm1}$ increased the transcriptional potential of non-priming-target genes, but no transcriptional change occurred due to a lack of upstream signal.
