# Supplementary material for: DDM1-mediated gene body DNA methylation is associated with inducible activation of defense-related genes in Arabidopsis
Source: Genome Biol. 2023 May 5;24:106. doi: 10.1186/s13059-023-02952-7 (PMC10161647; doi:10.1186/s13059-023-02952-7)
Supplement: Supplementary file 5 — Additional file 5. Review history. [file 13059_2023_2952_MOESM5_ESM.docx]

Review History

**First round of review**

**Reviewer 1**

**Were you able to assess all statistics in the manuscript, including the appropriateness of statistical tests used?**

Yes, and I have assessed the statistics in my report.

**Comments to author:**

This study by Lee et al. found that ddm1 mutant is more highly responsive to chemical priming than the wild-type. This is a very interesting finding. The authors then spent a lot of the efforts to try to explain the mechanisms underlying this phenomenon. They found that decreased GBM is perhaps associated with hyperactivation of defense genes in ddm1 under mild chemical priming. They also showed one gene with GBM change in ddm1 and three genes without GBM changes affect chemical priming. These are interesting findings and are of broad interest to others. However, it remains unexplained why the decreased methylation in ddm1 can affect chemical priming. It is clear from Figures 4 and 5 that DNA methylation is similar under various treatment, so the question is what is changing then? Maybe look at other chromatin modifications? Is it possible that loss of DNA methylation in ddm1 mutant facilitate the deposition/removal of other chromatin modifications after BAsub priming?

The authors used Figure 3 to define TE-LM and GB-LM, and then in Figures 4 and 5, they classify genes based on whether they have TE-LM or GB-LM. While this is a reasonable, but this interrupts the flow of the manuscript, and doesn't make a strong logic connection with Figure 2. I would suggest keeping using classifications in Figure 2 and look at how DNA methylation changed in ddm1 mutants under different treatment conditions.

I also think the authors should focus on the ddm1 story. The inclusion of many other mutants doesn't provide much insight and distract the readers' attention.

Specific comments:

1. P6, line 7, delete "eTE methylation", the met1 mutation affects CG methylation at heterochromatin as well. Similarly, delete "at eTEs" in line 11, it could be CHH methylation at gene bodies or other intergenic regions. Also in lines 18-19, I don't think you have evidence to say that "eTE hypomethylation enhances resistance to DC".

2. Figure S1A and S1B, the label in these two figures are confusing. The "mock" sample (white bar) in Figure S1A doesn't correspond to the "mock" label in S1B. Maybe change the "mock" in S1A to "mockDC3" and the "BA" to "BADC3"? There is a similar labeling problem in Figure 1A.

3. P6, line 48, why do you choose these three WRKY genes, are they related to defense priming?

4. Figure 1B and Figure S1D, are these the same qRT-PCR results? In Figure 1B, the expression difference between mockDC and BAsubDC in ddm1 mutant is smaller for all three genes than that in S1D. Also, the met1 mutant treated with BAsubDC is more resistance to DC than that treated with mockDC (Figure 1A), but there is no expression difference between these two treatments for both WRKY29 and WRKY75 (S1D), how to explain?

5. Figure 2B needs better sample labels.

6. P8 line 42, why do you choose 6 or 9 hpi for the comparison? There are significant changes in other time points as well.

7. I am wondering whether the "ddm1 hyper" and "ETI hyper" genes (in Figure 2C) are activated in drm1 drm2 (or ddc) mutants after weak priming.

8. The Osakabe paper (Nat Cell Biol, 2021 Apr;23(4):391-400) already shows that DDM1 is necessary and sufficient for the deposition of H2A.Z onto some TEs, so what is the point of Figure 2B?

9. P11, lines 11-13, "All TSSs...", this sentence is hard to understand.

10. Figure 4, it seems there is no correlation between DNA methylation changes and expression changes? I was wondering whether there is any DNA methylation change after DC treatment for the "ddm1 hyper" or "ddm1 sup" (Figure 2) genes. Are there any differentially methylated regions (DMRs) in these genes?

11. Figure 4F, how many genes were in each category? Is the y-axis showing the averaged fold changes of all genes in each specified categories? What test is used here?

12. Figure 5, again as Figure 4, there seems no difference in DNA methylation after DC treatment, and the gene expression change after DC treatment is not accompanied by methylation changes. How many of the GBMddm1 overlap with the "ddm1 hyper" or "ddm1 sup" (Figure 2) genes?

13. P13, line 33-34, this is inconsistent with the idea that "genes with body methylation tends to show high expression" as suggested in the literatures.

14. Figure 6, are there any difference in DNA methylation levels between these two types of GBM genes? How many of these GBMUC genes overlap with "ddm1 hyper" or "ddm1 sup" (Figure 2) genes?

15. Figure 7, how do the three WRKY genes respond to BA treatment in cpk4 mutant? In theory, they should respond similarly as the Col since CPK4 doesn't affect priming.

16. P19, in the Conclusion part, what do you mean by "in cis"? There are many genes that don't lose DNA methylation in ddm1 but show activated expression in ddm1 mutant after treatment. "The loss of GBM in ddm1 activates BABA-induced resistance", I think you only have evidence to suggest that BABA-induced resistance is accompanied by the loss of GBM in ddm1.

**Reviewer 2**

**Were you able to assess all statistics in the manuscript, including the appropriateness of statistical tests used?**

Yes, and I have assessed the statistics in my report.

**Comments to author:**

The manuscript by Lee et al. investigates the role of DNA methylation in mediating plant pathogen defense. The authors examined rapid transcriptional response to avirulent and virulent pathogens in the wildtype and ddm1 mutant, performed rigorous genomic analysis, and provided strong evidence for the association between reduced GBM (gene body methylation) and transcriptional potential of defense-related genes in ddm1 mutant. While I find the paper technically sound and well written, I do not think the results presented are sufficient to support the main conclusion that GBM plays a regulatory role in plant pathogen response. Therefore, the results, despite interesting, are descriptive, and do not make a significant advancement to the field.

The main problem is that DDM1, being a chromatin remodeler, has profound effect on the chromatin and does not only affect DNA methylation. Furthermore, although the ddm1 mutation causes general DNA hypomethylation, it actually only affects a very small fraction of the genes. It is unclear why only the GBM at these specific genes is affected, but not the others (this is consistent with the authors' results, showing that the genes that are demethylated in ddm1 are unconventional in chromatin features (Fig. 6)). These genes are indeed more activated during defense response, however, there is no evidence that such activation is caused by reduced GBM. A plausible alternative hypothesis is that the loss of DDM1 affects the chromatin properties of these "unconventional" genes, which potentiates their transcriptional activating and causes reduced methylation. In other words, although the authors' work demonstrates the association between reduced GBM and transcription, it does not prove causal relationship. In order to prove the latter, the authors need to analyze the met1 mutant, which actually eliminates most of the GBM.

Minor points:

1. The argument that "BAsub treatment enchanced disease resistance only in met1 and ddm1 plants … and did not affect resistance in drm1 drm2, cmt3, or ddc plants, suggesting that CG hypomethylation and BABA-induced chemical priming can synergistically induce disease resistance independently of CHG and CHH methylation" is invalid. CHH hypomethylation at heterochromatic TEs could also make the difference, for example, as it is caused by met1 and ddm1, but not drm1 drm2, cmt3 or ddc mutations. To exclude this possibility, the authors need to examine cmt2 and ddcc mutants. Similar logic applies to the conclusion on Page 7 Lines 16-18.

2. Most of the genomic analyses in the manuscript did not include sample sizes. The numbers are also not included in the Methods section. This needs to be fixed as it is important to know how many loci/genes were included in each analysis.

3. The paper used a large number of acronyms. It is important to make sure that they are explained in the main text. For example, on Page 12, GBM-IND was only explained in figure legends but not in the main text, making it difficult to understand.

**Authors Response**

**Point-by-point responses to the reviewers’ comments:**

**Reviewer #1-1**

This study by Lee et al. found that ddm1 mutant is more highly responsive to chemical priming than the wild-type. This is a very interesting finding. The authors then spent a lot of the efforts to try to explain the mechanisms underlying this phenomenon. They found that decreased GBM is perhaps associated with hyperactivation of defense genes in ddm1 under mild chemical priming. They also showed one gene with GBM change in ddm1 and three genes without GBM changes affect chemical priming. These are interesting findings and are of broad interest to others. However, it remains unexplained why the decreased methylation in ddm1 can affect chemical priming. It is clear from Figures 4 and 5 that DNA methylation is similar under various treatment, so the question is what is changing then?

*Reply: Our manuscript proposes DNA hypomethylation-mediated transcriptional potentiation. In ddm1, a subset of genes including defense-related genes are hypomethylated while CG DNA methylation is nearly eliminated in met1-3 (Figure 3). In pathogen treated condition (DC), ddm1 showed similar expression of defense-related genes compared to Col. However, ddm1 showed the increased expression of these genes after treatment of pathogen together with β-aminobutyric acid, a priming-inducing chemical (BAsub + DC, BAsubDC); Figure 1-2 and S1; see the table below). On the other hand, in met1-3, expression of defense-related genes was increased in DC compared to Col (Figure 1 and the table below). Comparison of the alteration in DNA methylation and gene expression between ddm1 and met1-3 in DC and BAsubDC (see the table below) suggests that there is a threshold for induction of defense-related genes in WT upon DC treatment, and DNA hypomethylation lowers the threshold, called “transcriptional potentiation”: a nearly zero threshold for met1-3, but a non-zero threshold for ddm1, which is lower than that in WT. Accordingly, met1-3 with the zero threshold induces defense-related genes upon DC treatment, whereas ddm1 with a non-zero threshold requires an additional defense signal, such as BAsub treatment, together with DC treatment to induce defense-related genes.*

*In summary, our results suggest that DNA hypomethylation only potentiates transcription of defense-related genes, and these genes are finally induced when plants encounter sufficient defense signals to overcome the threshold set by the degree of DNA hypomethylation. Consistent with our results, a recent study (Ong-Abdullah et al., 2015) in oil palm plants suggests that the demethylation of mCHG ('Karma' loci) causes hyper-activation of a novel splice form of the DEF1 gene. DNA hypomethylation is stable throughout inflorescence development, but the expression of DEF1 was induced only after stage 3 during the course of inflorescence development, even given the demethylation at Karma loci. We elaborated on this idea at the beginning of the discussion of the revised manuscript.*

*To further support the idea of GBM-mediated transcriptional potentiation, we tested transcriptional activation of gene body demethylated genes in Arabidopsis natural variants with variable GBM. Kawakatsu et al., reported over 1,000 single-base resolution methylomes of Arabidopsis natural variants (http://signal.salk.edu/1001.php) (Kawakatsu et al., 2016). By comparing transcriptional activation of genes between gene body methylated/de-methylated natural variants of Arabidopsis, we could test the causal relationship between reduced GBM and transcription (see Revised Figure 8F and Figure S11D below). Specifically, we focused on testing test whether variable GBM levels of the selected priming regulators (GPK1, BRL3, AT1G17230, and KIN11) could modulate transcriptional potentiation of these genes in natural variants, we ordered four natural variants (Bik-1, Kro-0, Ann-1 and Kas-1) that both GPK1 and BRL3 were demethylated (average gene mCG<0.05) and two natural variants (Uk-1 and Kondara) that have GBM at all of the priming regulators (average gene mCG≧0.1).*

*We then examined the expression of the priming regulators under BAsubDC with six natural variants and Col-0 using qRT-PCR (see Revised Figure 8F below). As expected, GPK1 and BRL3 showed augmented expression under BAsubDC only in variants that lost GBM (Figure 8F, vs. mockDC). AT1G17230 and KIN11 maintained GBM in all six natural variants we tested, and thus did not show significant transcriptional changes in BAsubDC compared to mockDC (Figure S8D).*

***Figure 8F*** *from the revised manuscript. Relative expression of novel priming regulators in natural variants (Top). The average GBM level of GPK1 and BRL3 in GBM variants and demethylated variants. (Bottom) Expression of GPK1 or BRL3 under mockDC condition was normalized to 1. Six natural variants and Col-0 were grouped by their GBM level at GPK1 and BRL3. Col-0, UK, and Kondara maintained GBM at both genes, and Bik, Kro, Ann, and Kas1 lost GBM at GPK1 and BRL3. RT-qPCR data from three replicates per natural variant were averaged (n=9 for GBM variants, n=12 for demethylated variants).*

***Figure S11D*** *from the revised manuscript. Relative expression of novel priming regulators in natural variants. (Left) The average GBM level of AT1G17230 and KIN11 in GBM variants and demethylated variants. (Right) Expression of AT1G17230 and KIN11 under mockDC condition was normalized to 1. Six natural variants and Col-0 were grouped by their GBM level at GPK1 and BRL3. Col-0, UK, and Kondara maintained GBM at both genes, and Bik, Kro, Ann, Kas1 lost GBM at GPK1 and BRL3. All natural variants maintained GBM at AT1G17230 and KIN11. RT-qPCR data from three replicates per natural variants were averaged (n=9 for GBM variants, n=12 for demethylated variants).*

**Reviewer #1-2**

Maybe look at other chromatin modifications? Is it possible that loss of DNA methylation in ddm1 mutant facilitate the deposition/removal of other chromatin modifications after BAsub priming?

*Reply: We have investigated the six chromatin properties other than GBM – H2A.W, H2A.Z, H3K4me3, H3K27me3, H3K9me2, and H1 – to explore the possibility that the altered chromatin compositions would trigger transcriptional potentiation, unfortunately, we could not find epigenetic modifications that correlate with gene expression change in Col-0 and ddm1 during the weakly primed defense response (BAsubDC).*

*1) Test defense priming response in h2a.w mutants*

*Osakabe et al., suggested that DDM1 deposits histone variant H2A.W to heterochromatin (Osakabe et al., 2021). When we reanalyzed Osakabe’s data, we found that DDM1 regulated H2A.W deposition at transposable element-like methylation (TE-LM) regions and barely altered H2A.W deposition at gene body-like methylation (GB-LM) regions even if there is severe demethylation (Figure 3B and 3E in the original manuscript), indicating a weak association between H2A.W and DNA methylation at GB-LM^ddm1^.*

***Figure 3 B,E*** *from the manuscript : Transposon-like methylation (TE-LM) and gene body-like methylation (GB-LM) patterns in ddm1 plants.*

*(B) Scatter plot of CG methylation change at TE-LM in relation to H2A.W change in ddm1. (E) Scatter plot of CG methylation change at GB-LM in relation to H2A.W change in ddm1.*

*Nevertheless, following the reviewer’s suggestion, we further tested during the revision whether the loss of H2A.W can modify defense priming using h2a.w knockout plants. Pathogen growth of h2a.w between mock and BAsub condition did not show significant difference, indicating that H2A.W does* *not affect defense priming (Figure S6C in the revised manuscript). Together with the aforementioned weak association between H2A.W and DNA methylation at GB-LM^ddm1^ (Figure 3B and 3E), these results suggest that H2A.W is not likely to affect chromatin alteration associated with GBM related transcriptional potentiation of genes in defense priming under BAsubDC condition.*

***Figure S6C*** *in the revised manuscript. Bacterial growth of Pseudomonas syringae pv. tomato DC3000 (DC) in H2A variant mutants with or without sub-optimal priming. For sub-optimal priming, water (mock control) or β -amino butyric acid (BAsub; 10 ml of 30 μg/ml per plant) was applied near roots two days prior pathogen inoculation. Bacterial growth was measured three days post-inoculation (dpi) (n = 5).*

*2) Test defense priming response in h2a.z mutants*

*Based on reviewers’ concern of chromatin modification changes, there is a possibility that H2A.Z can be associated with the gene body hypomethylation in ddm1, which can potentially affect transcription. In support, genes with high H2A.Z deposition and low DNA methylation were previously reported to show higher responsiveness to stress (Coleman-Derr and Zilberman, 2012).*

*During the revision, we thus tested whether the loss of h2a.z could modify defense priming using h2a.z knockout plants. Pathogen growth of h2a.z between mockDC and BAsubDC condition did not show any significant difference, indicating no effect on defense priming (see Figure S6C above). Simiar to H2A.W, accordingly, these results suggest that H2A.Z is also not likely to affect chromatin alteration associated with transcriptional potentiation of genes in defense priming under BAsubDC condition.*

*3) H3 histone modification patterns in Col-0 and ddm1 during primed defense response*

*No apparent links of H2A.W and H2A.Z to GBM related transcriptional potentiation in ddm1* *led us to further investigate H3 histone modification patterns around the priming regulators (GPK1, BRL3, AT1G17230, and KIN11) selected in our study using ChIP-qPCR. H3K4me3 levels of these regulators tended to be higher in ddm1 than in Col-0 under both mockDC and BAsubDC conditions (see Figure S12A in the revised manuscript below). In particular, significant increases were observed for GPK1 and KIN11 in all three replicate experiments. For some regulators (e.g., KIN11), H3K27me3 levels were elevated in ddm1, compared to in Col-0, but the overall levels were significantly lower compared to their H3K4me3 levels (see Figure S12C below). In contrast, H3K9me2 levels showed only marginal changes or no changes in ddm1. These results suggest a possibility that H3K4me3 levels can be associated with the increased expression of these regulators in ddm1. However, H3K4me3 levels showed no significant differences in ddm1 between mockDC and BAsubDC with no correlation with their expression changes between the two conditions (see the table above). Besides, there has been no evidence so far that DDM1 can directly affect H3K4me3. Accordingly, H3K4me3 appears not to be associated with transcriptional potentiation of these regulators in ddm1.*

*Moreover, because KIN11 is an indirect target with GBM weakly altered in ddm1, unlike a direct target GPK1(see Revised Figure 7A), this result suggests that H3K4me3 levels are not likely associated with GBM-mediated transcriptional potentiation. H3K4me3 levels near transcription start site (#1 in Figure S12), where DNA is accessible and does not require DDM1 activity, were elevated along with that in gene bodies (#2 and #3 in Figure S12), suggesting that change in H3K4me3 levels in ddm1 might be an indirect effect of the loss of DDM1. Our results thus suggest that it is more plausible that H3K4me3 levels might reflect the elevated expression of the four priming regulators in ddm1, rather than mediate GBM-associated transcriptional potentiation.*

***Figure S12*** *from the revised manuscript****.*** *Histone modification patterns around novel priming regulators.*

*(A) H3K4me3 enrichment around novel priming regulators in mockDC and BAsubDC condition in Col-0 and ddm1 plants. #1-3 primers amplify TSS-proximal region, gene body region and TES-*proximal *regions, respectively.*

*(B) H3K9me2 enrichment around four priming regulators.*

*(C) H3K27me3 enrichment around four priming regulators.*

*(A-C) Asterisks indicate statistically significant change in enrichment (p<0.05, Col vs. ddm1). p-values by Student’s t-test.*

*4) The effect of H1 on the transcription of genes with GBM^ddm1^*

*It has been reported that ddm1 mutation does not cause general DNA hypomethylation, but causes demethylation at H1-rich regions such as heterochromatin. For example, DDM1 was reported to regulate DNA methylation in concert with histone H1 (Zemach et al., 2013; Lyons and Zilberman, 2017), and H1 was enriched at heterochromatin and gene body methylated genes (Choi et al., 2020). Collectively, these studies suggest that DDM1 enables the access of DNA methylases to the H1-rich DNA, which provides a direct causal relationship between ddm1 mutation and DNA demethylation at H1-rich, inaccessible DNA. These previous data answer why ddm1 mutation did not globally affect GBM, but only a subset of genes.*

*We next investigated H1 patterns in ddm1 for* *regions with hypomethylation (GB-LM^ddm1^) and no methylation change (GB-LM^IND^) in gene body-like methylation (GB-LM). To this end,* *we re-analyzed the H1 data previously reported by Choi et al. H1 levels of GB-LM^ddm1^ were significantly higher than those of GB-LM^IND^ (see Revised Figure 3F below), indicating enrichment of H1 in GB-LM^ddm1^. Intriguingly, the methylation of GB-LM^ddm1^ at H1-rich regions was reduced in ddm1 and rescued in h1ddm1 (plants lack of H1 and ddm1) (see Revised Figure 3G below). In ddm1, H1 inhibits DNA methylases, which can freely methylate DNA in the absence of H1 in h1ddm1. Differently from GB-LM^ddm1^, GB-LM^IND^ showed no H1-dependent alteration patterns in methylation (see Revised Figure 3H below). These data suggest that GB-LM^ddm1^ in ddm1 is demethylated due to H1, which further supports reduced GBM only at a subset of genes (unconventional gbM genes), but not globally.*

***Figure 3*** *from the revised manuscript.*

***(F)*** *Histone H1.1 and H1.2 level at demethylated GB-LMs in ddm1 (GB-LM^ddm1^) and GB-LMs that keep DNA methylation in ddm1 (GB-LM^IND^). p-values from Student’s t-test.*

***(G and H)*** *Gene body CG DNA methylation level at GB-LM^ddm1^(G) and GB-LM^IND^ (H) in Col-0, h1, ddm1, and h1ddm1 plants.*

*Both H1 and CG methylation can suppress transcription of hTEs and jointly repress transcriptional initiation of antisense transcripts within gene body (Choi et al., 2020). Because GB-LM^ddm1^ has higher H1 levels than GB-LM^IND^ (Revised Figure 3F), we next tested whether H1 could suppress the expression of genes with GBM. When we compared the expression of genes with gene body hypomethylation in ddm1 (GBM^ddm1^) or genes with no GBM changes (GBM^IND^), GBM^ddm1^ genes with higher H1 levels were hyperactivated in h1 plants compared to in Col-0 (see Revised Figure 6D below). This result suggests that both GBM^ddm1^ and histone H1 play an important role for the expression of GBM genes, consistent with the previous finding that they jointly regulate the expression of hTEs and aberrant antisense transcription (Choi et al., 2020).*

*Taken together, histone H1 is needed for hypomethylation in ddm1 (see Revised Figure 3G above), and H1 suppresses expression of GBM^ddm1^ genes (see Revised Figure 6D below). However, transcriptional potentiation involves hypomethylation of defense related genes during defense priming response, followed by induction of these genes, which is not consistent with functions of H1 in hypomethylation and suppression of gene expression. Therefore, transcriptional potentiation of defense-related genes in ddm1 appears to be independent of H1.*

***Figure 6*** *from the revised manuscript*

***(D)*** *A boxplot of gene expression fold change (FC) of differentially expressed genes in h1 plants (compared to Col-0, q-value<0.05) overlapping with unmethylated genes (unmethyl), genes with DDM1-mediated gene body methylation (GBM^ddm1^), and genes with DDM1-independent gene body methylation (GBM^IND^). p-values by Student’s t-test.*

*Given little associations of histones with GBM related transcriptional potentiation associated defense priming response, although there could be other chromatin properties that we have not tested, we carefully propose a potential model for GBM related transcriptional potentiation and induction of defense related genes during defense priming response.*

*In met1 plants, since CG methylation is completely erased, GBM^ddm1^ genes are fully potentiated, resulting in spontaneous induction of these genes with zero threshold for induction. Therefore, met1 plants showed enhanced disease resistance without priming, and sub-optimal priming further fortified defense response against DC (Figure 1). During the revision, we analyzed the basal expression level of the four priming regulators in met1 and h1met1 plants, and the expression of GPK1 (demethylated in ddm1 and met1) and BRL3 (maintained GBM in ddm1 but lost GBM in met1) was upregulated in these plants without any treatment (Figure S11E), which further supports that global loss of CG methylation would cause spontaneous induction of these genes.*

*In ddm1 plants, GBM is partially demethylated, resulting in a non-zero threshold for induction of GBM^ddm1^ genes. Thus, transcriptional induction of GBM^ddm1^ genes still require an additional activation signal, namely treatment of a sub-optimal concentration of BABA (BAsubDC), which is sufficient to overcome the non-zero threshold for transcriptional induction of GBM^ddm1^ genes (Figure 1, 5 and S13).*

*In Col-0 plants, there are basal levels of GBM, which are much higher than those of GBM in ddm1, resulting in a higher threshold for induction of genes than that in ddm1. The higher threshold thus requires a stronger activation signal, namely treatment of an optimal concentration (higher than sub-optimal concentration) of BABA (BAoptDC) for induction of defense related genes (Figure S9, S10 and S13). However, a sub-optimal BABA treatment is not sufficient to overcome a high threshold for induction of defense-related genes.*

*Currently, we are investigating how BABA treatment activates induction of defense related genes. It has been reported that MPK3 and MPK6 play an important role for defense priming response (Conrath et al., 2015). To explore their roles in BABA-induced priming, we first examined whether defense priming caused by the optimal BABA treatment (BAoptDC) would be affected in mpk3 and mpk6 and found that the defense priming was compromised in these mutants (data not shown), consistent with their previously reported roles in defense priming (Conrath et al., 2015). In Col-0, GBM levels are reduced by the optimal BABA treatment under both BAopt and BAoptDC conditions (see Figure 1 for the letter to the editor below). We thus examined whether GBM levels are affected in mpk3 and mpk6 for the four priming regulators selected in this study and found that the BABA-induced reduction of GBM was significantly restored in these mutants (see Figure 1 for the letter to the editor below). Collectively, these preliminary data suggest that BABA-MAPK signaling can further decrease GBM, thereby promoting GBM related transcriptional potentiation of defense related genes (i.e., lowering a threshold for induction of genes) to enhance disease resistance.*

***Figure 1*** *for the letter to the editor.*

*Gene body DNA methylation level at novel priming regulators in Col-0, mpk3, and mpk6 plants under BAopt and BAoptDC condition. Optimal priming induced partial demethylation in Col-0 but not in mpk3 or mpk6 plants. Bisulfite treated gDNAs were amplified by PCR, then inserted into a pGEM®-T Easy Vector for cloning. DNAs from five colonies per condition were sequenced to calculate average DNA methylation level.*

**Reviewer #1-3**

The authors used Figure 3 to define TE-LM and GB-LM, and then in Figures 4 and 5, they classify genes based on whether they have TE-LM or GB-LM. While this is a reasonable, but this interrupts the flow of the manuscript, and doesn’t make a strong logic connection with Figure 2. I would suggest keeping using classifications in Figure 2 and look at how DNA methylation changed in ddm1 mutants under different treatment conditions.

*Reply: The DNA methylation at TE-LM and GB-LM are vastly different in their chromatin composition, and TE-LM is known to suppress gene expression strongly. Therefore, to examine the function of GB-LM for transcriptional potentiation, it is necessary to separate TE-LM and GB-LM.*

**Reviewer #1-4**

I also think the authors should focus on the ddm1 story. The inclusion of many other mutants doesn’t provide much insight and distract the readers’ attention.

*Reply: The disease resistance phenotype of DNA methylation mutants provides the valuable insight that mCG would be a potential regulator of primed defense response, as we compared the mutants with methylation loss at mCG, mCHG, and mCHH. However, the absence of drm1 drm2 cmt2 cmt3 (ddcc) plants in our original experiments made it difficult to comprehensively evaluate the effect of nonCG methylation during primed defense response. Therefore, during the revision, we tested primed defense response in ddcc plants, which helped to efficiently dissect the effect of CG and nonCG methylation during weakly primed defense response (see Revised Figure 1A in the revised manuscript). We think that the inclusion of ddcc plants clarified the role of CG and nonCG methylation on dense priming, thereby made the manuscript easier to digest. Therefore, we finally thought that it might be not necessary to exclude the analyses on DNA methylation mutants other than ddm1.*

*In addition, the comparison between met1 and ddm1, both of which lost mCG, also provides an additional insight that the different levels of methylation loss could differently affect basal expression level and inducible expression of defense-related genes, although proving this requires further investigation in the following figures in the manuscript.*

**Minor comments:**

**Reviewer #1-5**. P6, line 7, delete "eTE methylation", the met1 mutation affects CG methylation at heterochromatin as well. Similarly, delete "at eTEs" in line 11, it could be CHH methylation at gene bodies or other intergenic regions. Also in lines 18-19, I don't think you have evidence to say that "eTE hypomethylation enhances resistance to DC”.

*Reply: We mentioned eTE methylation because the loss of hTE methylation in cmt3 and drm1 drm2 cmt3 did not affect disease resistance compared to Col-0 and drm1 drm2. In contrast, specific loss of eTE methylation in drm1 drm2 plants enhanced disease resistance. However, when only Figure 1 is considered, mCG methylation loss of hTEs might have a specific role on the disease resistance. We, therefore, agree to delete ”eTE methylation” in line 7, "at eTEs" in line 11, and “eTE” in line 18-19.*

**Reviewer #1-6**.    Figure S1A and S1B, the label in these two figures are confusing. The "mock" sample (white bar) in Figure S1A doesn't correspond to the "mock" label in S1B. Maybe change the "mock" in S1A to "mockDC3" and the "BA" to "BADC3"? There is a similar labeling problem in Figure 1A.

*Reply: Both Figure S1A and Figure 1A present the result of the disease resistance assay to DC, which involves the inoculation of DC to plants. Therefore, we only labeled differential treatment conditions such as “mock”, “BA”, or “BAsub”. However, it seems that these labels caused unnecessary confusion. Therefore, as reviewer suggested, we changed label in Figure S1A to mockDC and BADC. Also, we changed label in Figure 1A to mockDC and BAsubDC.*

**Reviewer #1-7**.    P6, line 48, why do you choose these three WRKY genes, are they related to defense priming?

*Reply: WRKY genes are defense marker genes (Asai et al., Nature 415:977-983, 2002; Li et al., Plant cell 16:319-331, 2004; Encinas-Villarejo et al., Journal of experimental botany 60:3043-3065, 2009). Defense priming means hyper activation of defense response upon pathogen recognition, so we monitored the expression of defense marker genes to monitor primed defense response.*

**Reviewer #1-8**.    Figure 1B and Figure S1D, are these the same qRT-PCR results? In Figure 1B, the expression difference between mockDC and BAsubDC in ddm1 mutant is smaller for all three genes than that in S1D. Also, the met1 mutant treated with BAsubDC is more resistance to DC than that treated with mockDC (Figure 1A), but there is no expression difference between these two treatments for both WRKY29 and WRKY75 (S1D), how to explain?

*Reply: Figure 1B and Figure S1D are same qRT-PCR results. To clearly show the activation of WRKY gene expression between mockDC and BAsubDC in ddm1, we re-plotted WRKY gene expression between mockDC and BAsubDC in Figure S1D. We set mock in Col-0 to 1 in Figure 1B and mockDC in Col-0 to 1 Figure S1D. Y axis scale is -1 to 5 or -1 to 7 in Figure 1B and -1 to 3 or -1 to 4 in Figure S1D.*

***Figure S1****. Weakly primed defense response in ddm1 and met1 plants.*

***(D)*** *Expression of WRKY genes in Col-0, ddm1, and met1 plants one day post Pseudomonas syringae pv.tomato DC3000 inoculation with (BAsubDC) or without (mockDC) sub-optimal concentration of BABA treatment. Error bars indicate SEM (n ≧ 3). p-values by Student’s t-test. UBQ1 expression was used as an internal control.*

*Disease resistance is regulated by the induction of hundreds of defense-related genes, and that is the reason why we used 3 WRKY genes, but not one gene, as the marker for the defense response during priming. WRKY 70 expression was hyper activated under BAsubDC (vs. mockDC) in met1-3, which is consistent with enhanced disease resistance.*

**Reviewer #1-9**.    Figure 2B needs better sample labels.

*Reply: As the reviewer suggested, we changed sample labels.*

**Reviewer #1-10**. P8 line 42, why do you choose 6 or 9 hpi for the comparison? There are significant changes in other time points as well.

*Reply: 6 and 9 hpi shows the most significant difference in gene expression between virulent (DC) and avirulent (avrRpt2 and avrRpm1) pathogen responses.*

**Reviewer #1-11**.    I am wondering whether the "ddm1 hyper" and "ETI hyper" genes (in Figure 2C) are activated in drm1 drm2 (or ddc) mutants after weak priming.

*Reply: It would be interesting points testing "ddm1 hyper" and "ETI hyper" genes (in Figure 2C) are activated in drm1 drm2 (or drm1 drm2 cmt3) mutants after weak priming as the reviewer suggested, but we think that testing the role of nonCG methylation, especially at TEs, is another interesting topic for coming researches.*

**Reviewer #1-12**.    The Osakabe paper (Nat Cell Biol, 2021 Apr;23(4):391-400) already shows that DDM1 is necessary and sufficient for the deposition of H2A.Z onto some TEs, so what is the point of Figure 2B?

*Reply: Osakabe et al. reported that DDM1 is necessary and sufficient for the deposition of H2A.W (I guess H2A.Z is a typo) onto hTEs. Consistent with Osakabe’s report, we showed in Figure 3B that highly demethylated TE-LM in ddm1 lost H2A.W in ddm1. (I guess Figure 2B is a typo, too). In contrast, GB-LM demethylation was not associated with the depletion of H2A.W (Figure 3E). This result implies that DNA methylation loss at GB-LM in ddm1 is irrelevant to the change in H2A.W.*

**Reviewer #1-13**.    P11, lines 11-13, "All TSSs...", this sentence is hard to understand.

*Reply: TSS TE-LM genes shows methylation throughout promoter and gene body, which is overlapped with promoter TE-LM (pTE-LM) and gene body TE-LM (gbTE-LM). For the analysis, if pTE-LM and/or gbTE-LM genes are overlapped with TSS TE-LM genes, we excluded these genes from pTE-LM and/or gbTE-LM genes. We revise this sentence as below.*

*“Because all TSSs with TE-LM also had TE-LM throughout the promoter and gene body, we removed these genes from the pTE-LM and/or gbTE-LM groups (Figure 4A and 4B and Figure S4A–S4C).”*
**Reviewer #1-14**.    Figure 4, it seems there is no correlation between DNA methylation changes and expression changes? I was wondering whether there is any DNA methylation change after DC treatment for the "ddm1 hyper" or "ddm1 sup" (Figure 2) genes. Are there any differentially methylated regions (DMRs) in these genes?

*Reply: We discussed this comment in the reply to Reviewer #1-1.*

**Reviewer #1-15**.    Figure 4F, how many genes were in each category? Is the y-axis showing the averaged fold changes of all genes in each specified categories? What test is used here?

*Reply: Supplemental Data 1 of the revised manuscript provides the number of genes in each category. y-axis is showing the averaged fold changes of all differentially expressed genes in each specified category. We used Student’s t-test for comparison set.*

**Reviewer #1-16**.    Figure 5, again as Figure 4, there seems no difference in DNA methylation after DC treatment, and the gene expression change after DC treatment is not accompanied by methylation changes.

*Reply: We discussed this comment in the reply to Reviewer #1-1.*

How many of the GBMddm1 overlap with the "ddm1 hyper" or "ddm1 sup" (Figure 2) genes?

*Reply: We found that 237 GBM^ddm1^ genes overlap with “ddm1 hyper” and 150 with “ddm1 sup”.*

**Reviewer #1-17**. P13, line 33-34, this is inconsistent with the idea that "genes with body methylation tends to show high expression" as suggested in the literatures.

*Reply: In P13, line 33-34, we describe that "DNA methylation at the gene body (gbTE-LM and GBM) suppressed the transcriptional potential (Figure 4D and 4F and Figure 5C and 5E).” It means that DNA methylation at the gene body suppress inducibility of gene expression upon a certain cue, pathogen treatment in this case, not basal expression (discussed in Reviewer #1-1). Therefore, there is no inconsistency between suppression of gene expression inducibility and high expression of genes with body methylation.*

**Reviewer #1-18**.    Figure 6, are there any difference in DNA methylation levels between these two types of GBM genes? How many of these GBMUC genes overlap with "ddm1 hyper" or "ddm1 sup" (Figure 2) genes?

*Reply: As reviewer suggested, we analyzed DNA methylation difference of GBM^UC^ and GBM^C^, as well as the overlap of GBM^UC^ genes with “ddm1 hyper” and “ddm1 sup”.*

*When we compared DNA methylation levels of GBM^UC^ and GBM^C^, GBM^UC^ showed a slightly lower DNA methylation level compared to GBM^C^.*

*When we compared the DNA methylation levels of genes with GBM^UC^ and GBM^C^, genes with GBM^UC^ had lower average DNA methylation levels than genes with GBM^C^, suggesting that the proportion of gbM at GBM^UC^ is smaller than GBM^C^.*

*301* *GBM^UC^ genes overlap with “ddm1 hyper” and 174 with “ddm1 sup”. This indicates that “ddm1 hyper” is more enriched in GBM^UC^ than “ddm1 sup”.*

**Reviewer #1-19**.    Figure 7, how do the three WRKY genes respond to BA treatment in cpk4 mutant? In theory, they should respond similarly as the Col since CPK4 doesn't affect priming.

*Reply: As reviewer suggested, we checked WRKY genes expression in cpk4 plants (Figure S9B in the revised manuscript). Gene expression of WRKY70 and WRKY75 was significant increased in BADC compared to mockDC condition.*

***Figure S9B*** *from the revised manuscript. WRKY genes expression in cpk4 was measured 1-day post-inoculation (dpi). UBQ1 expression was used as an internal control. Asterisks indicate p-value by Student’s t-test. Error bars indicate SEM (n = 3).*

**Reviewer #1-20**. P19, in the Conclusion part, what do you mean by "in cis"? There are many genes that don't lose DNA methylation in ddm1 but show activated expression in ddm1 mutant after treatment. "The loss of GBM in ddm1 activates BABA-induced resistance", I think you only have evidence to suggest that BABA-induced resistance is accompanied by the loss of GBM in ddm1.

*Reply: “in cis” means that DDM1-mediated GBM suppressed inducible activation not other genes but itself, but it might not necessary to indicate ‘in cis’ on the manuscript. We removed this part. Also, we revised "The loss of GBM in ddm1 activates BABA-induced resistance" as "The loss of GBM in ddm1 potentiates BABA-induced resistance".*

**Reviewer #2-1**

The manuscript by Lee et al. investigates the role of DNA methylation in mediating plant pathogen defense. The authors examined rapid transcriptional response to avirulent and virulent pathogens in the wildtype and ddm1 mutant, performed rigorous genomic analysis, and provided strong evidence for the association between reduced GBM (gene body methylation) and transcriptional potential of defense-related genes in ddm1 mutant. While I find the paper technically sound and well written, I do not think the results presented are sufficient to support the main conclusion that GBM plays a regulatory role in plant pathogen response. Therefore, the results, despite interesting, are descriptive, and do not make a significant advancement to the field.
The main problem is that DDM1, being a chromatin remodeler, has profound effect on the chromatin and does not only affect DNA methylation. Furthermore, although the ddm1 mutation causes general DNA hypomethylation, it actually only affects a very small fraction of the genes.

It is unclear why only the GBM at these specific genes is affected, but not the others (this is consistent with the authors' results, showing that the genes that are demethylated in ddm1 are unconventional in chromatin features (Fig. 6)). These genes are indeed more activated during defense response, however, there is no evidence that such activation is caused by reduced GBM.

*Reply: Reviewer #2 asked why ddm1 mutation did not globally affect GBM, but only a subset of the genes and further suggested that chromatin properties might be altered in ddm1, which could cause the reduction of GBM.*

*Regarding the first issue, it has been reported that ddm1 mutation does not cause general DNA hypomethylation, but causes demethylation at H1-rich regions such as heterochromatin. For example, DDM1 was reported to regulate DNA methylation in concert with histone H1 (Zemach et al., 2013; Lyons and Zilberman, 2017), and H1 was enriched at heterochromatin and gene body methylated genes (Choi et al., 2020). Collectively, these studies suggest that DDM1 enables the access of DNA methylases to the H1-rich DNA, which provides a direct causal relationship between ddm1 mutation and DNA demethylation at H1-rich, inaccessible DNA. These previous data answer why ddm1 mutation did not globally affect GBM, but only a subset of genes.*

*Regarding the second issue, we next investigated H1 patterns in ddm1 for regions with hypomethylation (GB-LM^ddm1^) and no methylation change (GB-LM^IND^) in gene body-like methylation (GB-LM). To this end, we re-analyzed the H1 data previously reported by Choi et al. H1 levels of GB-LM^ddm1^ were significantly higher than those of GB-LM^IND^ (see Revised Figure 3F below), indicating enrichment of H1 in GB-LM^ddm1^. Intriguingly, the methylation of GB-LM^ddm1^ at H1-rich regions was reduced in ddm1 and rescued in h1ddm1 (plants lack of H1 and ddm1) (see Revised Figure 3G below). In ddm1, H1 inhibits DNA methylases, which can freely methylate DNA in the absence of H1 in h1ddm1. Differently from GB-LM^ddm1^, GB-LM^IND^ showed no H1-dependent alteration patterns in methylation (se Revised Figure 3H below). These data suggest that GB-LM^ddm1^ in ddm1 is demethylated due to H1, which further supports reduced GBM only at a subset of genes (unconventional gbM genes), but not globally.*

***Figure 3*** *from the revised manuscript.*

***(F)*** *Histone H1.1 and H1.2 level at demethylated GB-LMs in ddm1 (GB-LM^ddm1^) and GB-LMs that keep DNA methylation in ddm1 (GB-LM^IND^). p-values from Student’s t-test.*

***(G and H)*** *Gene body CG DNA methylation level at GB-LM^ddm1^(G) and GB-LM^IND^ (H) in Col-0, h1, ddm1, and h1ddm1 plants.*

*As the Reviewer #2’s comment “These genes are indeed more activated during defense response, however, there is no evidence that such activation is caused by reduced GBM.” is related to the Reviewer’s comment #2-2, we discussed this topic below in the response to Reviewer’s comment #2-2.*

**Reviewer #2-2**

A plausible alternative hypothesis is that the loss of DDM1 affects the chromatin properties of these "unconventional" genes, which potentiates their transcriptional activating and causes reduced methylation.

*Reply: During revision, we have investigated the six chromatin properties other than GBM – H2A.W, H2A.Z, H3K4me3, H3K27me3, H3K9me2, and H1 – to explore the possibility that the altered chromatin compositions would trigger transcriptional potentiation.*

*1) Test defense priming response in h2a.w mutants*

*We reanalyzed Osakabe’s data, and found that DDM1 regulated H2A.W deposition at transposable element-like methylation (TE-LM) regions and barely altered H2A.W deposition at gene body-like methylation (GB-LM) regions even if there is severe demethylation (Figure 3B and 3E in the original manuscript), indicating a weak association between H2A.W and DNA methylation at GB-LM^ddm1^.*

***Figure 3 B,E*** *from the manuscript : Transposon-like methylation (TE-LM) and gene body-like methylation (GB-LM) patterns in ddm1 plants.*

*(B) Scatter plot of CG methylation change at TE-LM in relation to H2A.W change in ddm1. (E) Scatter plot of CG methylation change at GB-LM in relation to H2A.W change in ddm1.*

*Nevertheless, following the reviewer’s suggestion, we further tested during the revision whether the loss of H2A.W can modify defense priming using h2a.w knockout plants. Pathogen growth of h2a.w between mock and BAsub condition did not show significant difference, indicating that H2A.W does not affect defense priming (Figure S6C in the revised manuscript). Together with the aforementioned weak association between H2A.W and DNA methylation at GB-LM^ddm1^ (Figure 3B and 3E), these results suggest that H2A.W is not likely to affect chromatin alteration associated with GBM related transcriptional potentiation of genes in defense priming under BAsubDC condition.*

***Figure S6C*** *in the revised manuscript. Bacterial growth of Pseudomonas syringae pv. tomato DC3000 (DC) in H2A variant mutants with or without sub-optimal priming. For sub-optimal priming, water (mock control) or β -amino butyric acid (BAsub; 10 ml of 30 μg/ml per plant) was applied near roots two days prior pathogen inoculation. Bacterial growth was measured three days post-inoculation (dpi) (n = 5).*

*2) Test defense priming response in h2a.z mutants*

*There is another possibility that H2A.Z can be associated with the gene body hypomethylation in ddm1, which can potentially affect transcription. In support, genes with high H2A.Z deposition and low DNA methylation were previously reported to show higher responsiveness to stress (Coleman-Derr and Zilberman, 2012).*

*During the revision, we thus tested whether the loss of h2a.z could modify defense priming using h2a.z knockout plants. Pathogen growth of h2a.z between mockDC and BAsubDC condition did not show any significant difference, indicating no effect on defense priming (see Figure S6C above). Simiar to H2A.W, accordingly, these results suggest that H2A.Z is also not likely to affect chromatin alteration associated with transcriptional potentiation of genes in defense priming under BAsubDC condition.*

*3) H3 histone modification patterns in Col-0 and ddm1 during primed defense response*

*No apparent links of H2A.W and H2A.Z to GBM related transcriptional potentiation in ddm1 led us to further investigate H3 histone modification patterns around the priming regulators (GPK1, BRL3, AT1G17230, and KIN11) selected in our study using ChIP-qPCR. H3K4me3 levels of these regulators tended to be higher in ddm1 than in Col-0 under both mockDC and BAsubDC conditions (see Figure S12A in the revised manuscript below). In particular, significant increases were observed for GPK1 and KIN11 in all three replicate experiments. For some regulators (e.g., KIN11), H3K27me3 levels were elevated in ddm1, compared to in Col-0, but the overall levels were significantly lower* *compared to their H3K4me3 levels (see Figure S12C below). In contrast, H3K9me2 levels showed only marginal changes or no changes in ddm1. These results suggest a possibility that H3K4me3 levels can be associated with the increased expression of these regulators in ddm1. However, H3K4me3 levels showed no significant differences in ddm1 between mockDC and BAsubDC with no correlation with their expression changes between the two conditions (see the table above). Besides, there has been no evidence so far that DDM1 can directly affect H3K4me3. Accordingly, H3K4me3 appears not to be associated with transcriptional potentiation of these regulators in ddm1.*

*Moreover, because KIN11 is an indirect target with GBM weakly altered in ddm1, unlike a direct target GPK1 (see Revised Figure 7A), this result suggests that H3K4me3 levels are not likely associated with GBM-mediated transcriptional potentiation. H3K4me3 levels near transcription start site (#1 in Figure S12), where DNA is accessible and does not require DDM1 activity, were elevated along with that in gene bodies (#2 and #3 in Figure S12), suggesting that change in H3K4me3 levels in ddm1 might be an indirect effect of the loss of DDM1. Our results thus suggest that it is more plausible that H3K4me3 levels might reflect the elevated expression of the four priming regulators in ddm1, rather than mediate GBM-associated transcriptional potentiation.*

***Figure S12*** *from the revised manuscript****.*** *Histone modification patterns around novel priming regulators.*

*(A) H3K4me3 enrichment around novel priming regulators in mockDC and BAsubDC condition in Col-0 and ddm1 plants. #1-3 primers amplify TSS-proximal region, gene body region and TES-proximal regions, respectively.*

*(B) H3K9me2 enrichment around four priming regulators.*

*(C) H3K27me3 enrichment around four priming regulators.*

*(A-C) Asterisks indicate statistically significant change in enrichment (p<0.05, Col vs. ddm1). p-values by Student’s t-test.*

*4) The effect of H1 on the transcription of genes with GBM^ddm1^.*

*Both H1 and CG methylation can suppress transcription of hTEs and jointly repress transcriptional initiation of antisense transcripts within gene body (Choi et al., 2020). Because GB-LM^ddm1^ has higher H1 levels than GB-LM^IND^ (Revised Figure 3F), we next tested whether H1 could suppress the expression of genes with GBM. When we compared the expression of genes with gene body hypomethylation in ddm1 (GBM^ddm1^) or genes with no GBM changes (GBM^IND^), GBM^ddm1^ genes with higher H1 levels were hyperactivated in h1 plants compared to in Col-0 (see Revised Figure 6D below). This result suggests that both GBM^ddm1^ and histone H1 play an important role for the expression of GBM genes, consistent with the previous finding that they jointly regulate the expression of hTEs and aberrant antisense transcription (Choi et al., 2020).*

*Taken together, histone H1 is needed for hypomethylation in ddm1 (see Revised Figure 3G above), and H1 suppresses expression of GBM^ddm1^ genes (see Revised Figure 6D below). However, transcriptional potentiation involves hypomethylation of defense related genes during defense priming response, followed by induction of these genes, which is not consistent with functions of H1 in hypomethylation and suppression of gene expression. Therefore, transcriptional potentiation of defense-related genes in ddm1 appears to be independent of H1.*

***Figure 6*** *from the revised manuscript*

***(D)*** *A boxplot of gene expression fold change (FC) of differentially expressed genes in h1 plants (compared to Col-0, q-value<0.05) overlapping with unmethylated genes (unmethyl), genes with DDM1-mediated gene body methylation (GBM^ddm1^), and genes with DDM1-independent gene body methylation (GBM^IND^). p-values by Student’s t-test.*

*In summary, although we have investigated the six chromatin properties other than GBM – H2A.W, H2A.Z, H3K4me3, H3K27me3, H3K9me2, and H1 – to explore the possibility that the altered chromatin compositions would trigger transcriptional potentiation, unfortunately, we could not find* *epigenetic modifications that correlate with gene expression change in Col-0 and ddm1 during the weakly primed defense response (BAsubDC).*

**Reviewer #2-3**

In other words, although the authors' work demonstrates the association between reduced GBM and transcription, it does not prove causal relationship. In order to prove the latter, the authors need to analyze the met1 mutant, which actually eliminates most of the GBM.

*Reply: Reviewer #2 suggested analyzing met1 mutant to test the causal relationship between reduced GBM and transcription. However, one of the pitfalls in the use of met1 plants is that met1 completely removes mCG in Arabidopsis. Therefore, we cannot compare genes that keep gene body methylation and the gene that lose gene body methylation. In addition, the complete loss of mCG in met1 perturbs global chromatin composition e.g., triggering redistribution of H3K9me2 to genes and H3K27me3 to TEs (Deleris et al., 2012), which can further complicate the analysis to evaluate the effect of GBM on gene expression.*

*To overcome this issue, we tested transcriptional activation of gene body demethylated genes in Arabidopsis natural variants with variable GBM. Kawakatsu et al., reported over 1,000 single-base resolution methylomes of Arabidopsis natural variants (*[*http://signal.salk.edu/1001.php*](http://signal.salk.edu/1001.php)*) (Kawakatsu et al., 2016). By comparing transcriptional activation of genes between gene body methylated/de-methylated natural variants of Arabidopsis, we could test the causal relationship between reduced GBM and transcription (see Revised Figure 8F and Figure S11D below).*

*1) Test transcriptional activation of gene body demethylated genes in Arabidopsis natural variants*

*To test whether variable GBM levels of the selected priming regulators (GPK1, BRL3, AT1G17230, and KIN11) could modulate transcriptional potentiation of these genes in natural variants, we ordered four natural variants (Bik-1, Kro-0, Ann-1 and Kas-1) that both GPK1 and BRL3 were demethylated (average gene mCG<0.05) and two natural variants (Uk-1 and Kondara) that have GBM at all of the priming regulators (average gene mCG≧0.1).*

*We then examined the expression of the priming regulators under BAsubDC with six natural variants and Col-0 using qRT-PCR (see Revised Figure 8F below). As expected, GPK1 and BRL3 showed augmented expression under BAsubDC only in variants that lost GBM (Figure 8F, vs. mockDC). AT1G17230 and KIN11 maintained GBM in all six natural variants we tested, and thus did not show significant transcriptional changes in BAsubDC compared to mockDC (Figure S8D).*

***Figure 8F*** *from the revised manuscript. Relative expression of novel priming regulators in natural variants (Top). The average GBM level of GPK1 and BRL3 in GBM variants and demethylated variants. (Bottom) Expression of GPK1 or BRL3 under mockDC condition was normalized to 1. Six natural variants and Col-0 were grouped by their GBM level at GPK1 and BRL3. Col-0, UK, and Kondara maintained GBM at both genes, and Bik, Kro, Ann, and Kas1 lost GBM at GPK1 and BRL3. RT-qPCR data from three replicates per natural variant were averaged (n=9 for GBM variants, n=12 for demethylated variants).*

***Figure S11D*** *from the revised manuscript. Relative expression of novel priming regulators in natural variants. (Left) The average GBM level of AT1G17230 and KIN11 in GBM variants and demethylated variants. (Right) Expression of AT1G17230 and KIN11 under mockDC condition was normalized to 1. Six natural variants and Col-0 were grouped by their GBM level at GPK1 and BRL3. Col-0, UK, and Kondara maintained GBM at both genes, and Bik, Kro, Ann, Kas1 lost GBM at GPK1 and BRL3. All natural variants maintained GBM at AT1G17230 and KIN11. RT-qPCR data from three replicates per natural variants were averaged (n=9 for GBM variants, n=12 for demethylated variants).*

*Given little associations of histones with GBM related transcriptional potentiation associated defense priming response (response to Reviewer’s comment #2-2), and the causal relationship between GBM levels and transcriptional potentiation of novel priming regulators in natural variants, although there could be other chromatin properties that we have not tested, we carefully propose a potential model for GBM related transcriptional potentiation and induction of defense related genes during defense priming response.*

*In met1 plants, since CG methylation is completely erased, GBM^ddm1^ genes are fully potentiated, resulting in spontaneous induction of these genes with zero threshold for induction. Therefore, met1 plants showed enhanced disease resistance without priming, and sub-optimal priming further fortified defense response against DC (Figure 1). During the revision, we analyzed the basal expression level of the four priming regulators in met1 and h1met1 plants, and the expression of GPK1 (demethylated in ddm1 and met1) and BRL3 (maintained GBM in ddm1 but lost GBM in met1) was upregulated in these plants without any treatment (Figure S11E), which further supports that global loss of CG methylation would cause spontaneous induction of these genes.*

*In ddm1 plants, GBM is partially demethylated, resulting in a non-zero threshold for induction of GBM^ddm1^ genes. Thus, transcriptional induction of GBM^ddm1^ genes still require an additional activation signal, namely treatment of a sub-optimal concentration of BABA (BAsubDC), which is sufficient to overcome the non-zero threshold for transcriptional induction of GBM^ddm1^ genes (Figure 1, 5 and S13).*

*In Col-0 plants, there are basal levels of GBM, which are much higher than those of GBM in ddm1, resulting in a higher threshold for induction of genes than that in ddm1. The higher threshold thus requires a stronger activation signal, namely treatment of an optimal concentration (higher than sub-optimal concentration) of BABA (BAoptDC) for induction of defense related genes (Figure S9, S10 and S13). However, a sub-optimal BABA treatment is not sufficient to overcome a high threshold for induction of defense-related genes.*

*Currently, we are investigating how BABA treatment activates induction of defense related genes. It has been reported that MPK3 and MPK6 play an important role for defense priming response (Conrath et al., 2015). To explore their roles in BABA-induced priming, we first examined whether defense priming caused by the optimal BABA treatment (BAoptDC) would be affected in mpk3 and mpk6 and found that the defense priming was compromised in these mutants (data not shown), consistent with their previously reported roles in defense priming (Conrath et al., 2015). In Col-0, GBM levels are reduced by the optimal BABA treatment under both BAopt and BAoptDC conditions (see* *Figure 1 for the letter to the editor below). We thus examined whether GBM levels are affected in mpk3 and mpk6 for the four priming regulators selected in this study and found that the BABA-induced reduction of GBM was significantly restored in these mutants (see Figure 1 for the letter to the editor below). Collectively, these preliminary data suggest that BABA-MAPK signaling can further decrease GBM, thereby promoting GBM related transcriptional potentiation of defense related genes (i.e., lowering a threshold for induction of genes) to enhance disease resistance.*

***Figure 1*** *for the letter to the editor.*

*Gene body DNA methylation level at novel priming regulators in Col-0, mpk3, and mpk6 plants under BAopt and BAoptDC condition. Optimal priming induced partial demethylation in Col-0 but not in mpk3 or mpk6 plants. Bisulfite treated gDNAs were amplified by PCR, then inserted into a pGEM®-T Easy Vector for cloning. DNAs from five colonies per condition were sequenced to calculate average DNA methylation level.*

Minor comments:
**Reviewer #2-4**. The argument that "BAsub treatment enchanced disease resistance only in met1 and ddm1 plants … and did not affect resistance in drm1 drm2, cmt3, or ddc plants, suggesting that CG hypomethylation and BABA-induced chemical priming can synergistically induce disease resistance independently of CHG and CHH methylation" is invalid. CHH hypomethylation at heterochromatic TEs could also make the difference, for example, as it is caused by met1 and ddm1, but not drm1 drm2, cmt3 or ddc mutations. To exclude this possibility, the authors need to examine cmt2 and ddcc mutants. Similar logic applies to the conclusion on Page 7 Lines 16-18.

*Reply: As reviewer suggested, we checked pathogen growth in ddcc mutants and presented this data in the revised Figure 1, which greatly helped to dissect the effect of CG and nonCG on primed defense response. We thank for the reviewer’s suggestion.*

***Figure 1****. Augmented defense response against the biotrophic pathogen in CG-hypomethylated mutants.*

***(A)*** *Bacterial growth of Pseudomonas syringae pv. tomato DC3000 (DC) in DNA methylation mutants with or without sub-optimal priming. For sub-optimal priming, water (mock control) or β -amino butyric acid (BAsub; 10 ml of 30 μg/ml per plant) was applied near roots two days prior pathogen inoculation. Bacterial growth was measured three days post-inoculation (dpi) from at least five biological replicates (n ≧ 5). ddc, ddcc and rdd indicate drm1 drm2 cmt3, drm1 drm2 cmt2 cmt3 and ros1 dml2 dml3 plants, respectively. p-values by Student’s t-test.*

**Reviewer #2-5**. Most of the genomic analyses in the manuscript did not include sample sizes. The numbers are also not included in the Methods section. This needs to be fixed as it is important to know how many loci/genes were included in each analysis.

*Reply: As reviewer suggested, we added loci/gene numbers of genomic analyses in the Supplemental Data 1 of the revised manuscript.*

**Reviewer #2-6**.    The paper used a large number of acronyms. It is important to make sure that they are explained in the main text. For example, on Page 12, GBM-IND was only explained in figure legends but not in the main text, making it difficult to understand.

*Reply: We added explanation of GBM^IND^ in the main text as*

*.. 2) DDM1-independent GB-LM in promoter or gene body (pGB-LM^IND^ or GBM^IND^) under the BAsubDC condition (Figure 5E).*

***References***

***Choi, J., Lyons, D.B., Kim, M.Y., Moore, J.D., and Zilberman, D.*** *(2020). DNA Methylation and Histone H1 Jointly Repress Transposable Elements and Aberrant Intragenic Transcripts. Molecular Cell* ***77,*** *310-323.e317.*

***Coleman-Derr, D., and Zilberman, D.*** *(2012). Deposition of Histone Variant H2A.Z within Gene Bodies Regulates Responsive Genes. PLoS Genet* ***8,*** *e1002988.*

***Conrath, U., Beckers, G.J., Langenbach, C.J., and Jaskiewicz, M.R.*** *(2015). Priming for enhanced defense. Annual review of phytopathology* ***53,*** *97-119.*

***Deleris, A., Stroud, H., Bernatavichute, Y., Johnson, E., Klein, G., Schubert, D., and Jacobsen, S.E.*** *(2012). Loss of the DNA Methyltransferase MET1 Induces H3K9 Hypermethylation at PcG Target Genes and Redistribution of H3K27 Trimethylation to Transposons in Arabidopsis thaliana. PLOS Genetics* ***8,*** *e1003062.*

***Kawakatsu, T., Huang, S.-s.C., Jupe, F., Sasaki, E., Schmitz, R.J., Urich, M.A., Castanon, R., Nery, J.R., Barragan, C., He, Y., Chen, H., Dubin, M., Lee, C.-R., Wang, C., Bemm, F., Becker, C., O’Neil, R., O’Malley, R.C., Quarless, D.X., Alonso-Blanco, C., Andrade, J., Becker, C., Bemm, F., Bergelson, J., Borgwardt, K., Chae, E., Dezwaan, T., Ding, W., Ecker, J.R., Expósito-Alonso, M., Farlow, A., Fitz, J., Gan, X., Grimm, D.G., Hancock, A., Henz, S.R., Holm, S., Horton, M., Jarsulic, M., Kerstetter, R.A., Korte, A., Korte, P., Lanz, C., Lee, C.-R., Meng, D., Michael, T.P., Mott, R., Muliyati, N.W., Nägele, T., Nagler, M., Nizhynska, V., Nordborg, M., Novikova, P., Picó, F.X., Platzer, A., Rabanal, F.A., Rodriguez, A., Rowan, B.A., Salomé, P.A., Schmid, K., Schmitz, R.J., Seren, Ü., Sperone, F.G., Sudkamp, M., Svardal, H., Tanzer, M.M., Todd, D., Volchenboum, S.L., Wang, C., Wang, G., Wang, X., Weckwerth, W., Weigel, D., Zhou, X., Schork, N.J., Weigel, D., Nordborg, M., and Ecker, J.R.*** *(2016). Epigenomic Diversity in a Global Collection of Arabidopsis thaliana Accessions. Cell* ***166,*** *492-505.*

***Lyons, D.B., and Zilberman, D.*** *(2017). DDM1 and Lsh remodelers allow methylation of DNA wrapped in nucleosomes. eLife* ***6,*** *e30674.*

***Ong-Abdullah, M., Ordway, J.M., Jiang, N., Ooi, S.-E., Kok, S.-Y., Sarpan, N., Azimi, N., Hashim, A.T., Ishak, Z., Rosli, S.K., Malike, F.A., Bakar, N.A.A., Marjuni, M., Abdullah, N., Yaakub, Z., Amiruddin, M.D., Nookiah, R., Singh, R., Low, E.-T.L., Chan, K.-L., Azizi, N., Smith, S.W., Bacher, B., Budiman, M.A., Van Brunt, A., Wischmeyer, C., Beil, M., Hogan, M., Lakey, N., Lim, C.-C., Arulandoo, X., Wong, C.-K., Choo, C.-N., Wong, W.-C., Kwan, Y.-Y., Alwee, S.S.R.S., Sambanthamurthi, R., and Martienssen, R.A.*** *(2015). Loss of Karma transposon methylation underlies the mantled somaclonal variant of oil palm. Nature* ***525,*** *533-537.*

***Osakabe, A., Jamge, B., Axelsson, E., Montgomery, S.A., Akimcheva, S., Kuehn, A.L., Pisupati, R., Lorković, Z.J., Yelagandula, R., Kakutani, T., and Berger, F.*** *(2021). The chromatin remodeler DDM1 prevents transposon mobility through deposition of histone variant H2A.W. Nature Cell Biology* ***23,*** *391-400.*

***Zemach, A., Kim, M., Hsieh, P.-H., Coleman-Derr, D., Eshed-Williams, L., Thao, K., Harmer, S., and Zilberman, D.*** *(2013). The Arabidopsis Nucleosome Remodeler DDM1 Allows DNA Methyltransferases to Access H1-Containing Heterochromatin. Cell* ***153,*** *193-205.*

**Second round of review**

**Reviewer 1**

The authors did a nice job in revising the manuscript and it now reads much better than the first version. There are still some problems that need to be addressed.

1. The use of lots of author-defined abbreviations made the manuscript not friendly. For example, on page 7, the authors defined TE-LM (TE-like methylation) and GB-LM (gene body-like methylation). “TE-LM” is methylation that occurs in all three contexts (C methylation) and “GB-LM” is “CG methylation”? The naming system made the manuscript hard to read. It should be improved, for example, using conventional abbreviations etc.
2. The authors compared the phenotypes of different epigenetic mutants and implied that loss of CG and not CHG/CHH methylation potentiates transcription of defense-related genes. If this is the case, the vim mutants would be expected to show a similar effect as the met1 mutant.
3. The Conclusion is not precise. Page 9 Lines 47-50, “Overall, both TSS TE-LM…….”; Page 13, Lines 14-16, “We identified……” ; based on these descriptions, not only GBM potentiates transcription of defense-related genes as concluded in Conclusion.
4. Figure 2D/F, explain how the tests were performed in Methods.
5. Figure 7, BAsubDC condition was used for other experiments except this one, explain why “BADC” was used here instead of “BAsubDC”.
6. Figure 8B/8C/8E, use capitals for the first word in y axis title.
7. Figure 8F, add dots (or other symbols) to represent the value of each Arabidopsis accession.

**Reviewer 3**

In plants, gene body methylation (GBM) is known to be highly variable both at the inter- and intra-species level, and is positively associated with gene expression at many loci. In this study, Lee et al. demonstrate that GBM loci can also be responsible for potentiating transcriptional changes when methylation levels are reduced. By testing ddm-1 and Col-0 plants for responses to pathogen inoculation upon priming, the authors find that a subset of differentially regulated genes (ddm1 vs Col-0) in primed plants carry hypomethylated GBM and function in defense response. Hypomethylated GBM loci in ddm-1 also exhibit variable chromatin marks. Closer inspection of a few priming regulator genes that are upregulated in ddm1 mutants (upon pathogen inoculation after priming) reveals that the association between gene body hypomethylation and increased gene expression is consistent with patterns observed in natural variants.

The authors show that several genes are differentially expressed between ddm-1 and Col-0 when both plants are primed with BABA and infected with DC3000, but that methylation differences do not vary during all treatments (mock, BAsub, mockDC, BAsubDC). It is proposed that this may be due to a transcription potentiation effect. While gene expression changes (ddm1 vs Col-0) have been analysed under all four treatments, a similar quantitative analysis of differential methylation and differential chromatin properties in the same plants (and for the same treatments) is missing. Such an analysis would provide a clearer understanding of how priming influences epigenetic and epigenomic marks to differentially regulate genes during pathogen inoculation.

From the results, it is clear that GBMddm1 loci are distinct from other GBM loci in their expression levels and chromatin properties - yet, only a small proportion (<5%) of GBMddm1 loci overlap with all DEGs (ddm1/Col-0) in all treatment conditions (as shown in Supplementary Dataset 1). Therefore, a majority of differential expression changes in the BAsubDC condition are still not accounted for by DDM-1 mediated gene body methylation. It may also be interesting to examine GBM at genes that are differentially expressed in BAsubDC but are not differentially expressed in mockDC condition.

Overall, the manuscript is well written and presented. While the authors have carried out extensive analyses to examine hypomethylated GBM loci in ddm-1 mutants, their results are largely restricted to examining differences in methylation between ddm-1 and Col-0 in standard conditions, of which only a few loci GBMddm1 loci are associated with transcriptional changes upon pathogen inoculation. It is interesting that differentially expressed (upregulated) genes exhibiting hypomethylated GBM (GBMddm1) are associated with defense response, but it is unclear whether this is due to the direct effect of DDM-1 mediated GBM or an indirect effect involving both methylation changes and changes in chromatin properties. Therefore, despite the results being well explained, the findings do not provide sufficient advancement in our current understanding of adaptive epigenetic regulatory mechanisms.

Minor comments:

* The authors split their analysis of GBM by comparing loci that have lost methylation in ddm-1 (GBMddm1) with all other GBM loci (GBMIND), which have unchanged methylation in Col-0. However, Figure 3D shows that GBMIND loci also include some genes that gain methylation in ddm-1. Therefore, it would be more informative if hypomethylated loci, hypermethylated loci and loci with unchanged methylation would be distinguished in all downstream analyses.
* The authors could be more clear in indicating which genotypes were used for calculating methylation levels, and which genotypes were compared for identifying differentially expressed genes (both in the main text and figures). For example, it is understood that GBMddm1 methylation levels are estimated based on the ddm-1 mutant, but are methylation levels at GBMIND loci based on ddm-1 or Col-0?
* Supplementary dataset 1 and Figure 2C,2E can include number of DEGs identified in ddm-1 hyper, ddm-1 sup, ETI hyper, ETI sup and the overlaps between these DEGs with basal ddm-1 DEGs (all DEGs). The intersections between GBMddm1, GBMIND loci with ddm-1 hyper and ddm-1 sup DEGs is also not indicated in the text or supplement.

**Authors Response**

**Point-by-point responses to the reviewers’ comments:**

**Reviewer reports:**
Reviewer #1:

The authors did a nice job in revising the manuscript and it now reads much better than the first version. There are still some problems that need to be addressed.

1. The use of lots of author-defined abbreviations made the manuscript not friendly. For example, on page 7, the authors defined TE-LM (TE-like methylation) and GB-LM (gene body-like methylation). “TE-LM” is methylation that occurs in all three contexts (C methylation) and “GB-LM” is “CG methylation”? The naming system made the manuscript hard to read. It should be improved, for example, using conventional abbreviations etc.

*Reply: Thank you for the comment. As suggested, we have replaced the non-common abbreviations with the more frequently used abbreviations with further clarification of their definitions as detailed below.*

*One distinctive feature of plant DNA methylation is that gene body tends to be associated with CG methylation while transposons have both CG and nonCG (CHG and CHH) methylation. Therefore, ‘CG methylation’ have been referred to as ‘gene body methylation’ and abbreviated as ‘GBM’ in many literatures (Dubin et al., 2015; Takuno et al., 2017; Zilberman, 2017; Ji et al., 2018; Horvath et al., 2019; Liu et al., 2021; Muyle et al., 2021; Zhang et al., 2021; Srikant et al., 2022). However, because CG methylation does not always occur in gene bodies, the term ‘gene body-like methylation’ has been widely used to indicate ‘gene body CG methylation’ in non-gene bodies and abbreviated as ‘GBM-like’ as described previously (Ji et al., 2018; Srikant et al., 2022).*

*Since we previously used ‘GBM’ and ‘GB-LM’ to refer to ‘gene body CG methylation’ in gene bodies and non-gene bodies, respectively, we now changed the latter abbreviation ‘GB-LM’ with ‘GBM-like’ according to the conventions in the above literatures (Ji et al., 2018; Srikant et al., 2022). Moreover, we used ‘pGB-LM’ to specifically refer to ‘gene body-like methylation’ in the promoter, but this detailed categorization has not been reported previously. Thus, following the above convention, we changed ‘pGB-LM’ with ‘pGBM-like’.*

*On the other hand, when both CG and nonCG methylations simultaneously occur in transposons, the term ‘transposon methylation’ has been used to indicate those methylations. In a number of literatures, when both CG and nonCG methylations simultaneously occur in non-transposons (e.g., repeats or repeat-like gene body regions), the term ‘transposon-like methylation’ has been often used. Despite a bit ambiguity, however, these two types of methylations have been conventionally abbreviated as ‘TEM’ (Bewick and Schmitz, 2017; Ji et al., 2019).*

*Since we previously used ‘TE-LM’ to refer to ‘methylation occurring at both CG and nonCG contexts in either transposons or non-transposons’, we have thus changed the abbreviation ‘TE-LM’ with ‘TEM-like’ according to the conventions in the above literatures (Bewick and Schmitz, 2017; Ji et al., 2019). Of note, we used ‘TEM-like’ to be consistent with ‘GBM-like’, instead of the ambiguous ‘TEM’. Similar to ‘pGBM-like’, we previously used ‘gbTE-LM’ and ‘pTE-LM’ to specifically refer to ‘methylations occurring at both CG and nonCG contexts’ in gene bodies and promoters, respectively, but this detailed categorization has not been reported previously. Thus, following the above convention, we changed ‘gbTE-LM’ and ‘pTE-LM’ with ‘gbTEM-like’ and ‘pTEM-like’, respectively.*

*Reference for Reviewer#1-1*

***Bewick, A.J., and Schmitz, R.J.*** *(2017). Gene body DNA methylation in plants. Current Opinion in Plant Biology* ***36,*** *103-110.*

***Dubin, M.J., Zhang, P., Meng, D., Remigereau, M.-S., Osborne, E.J., Paolo Casale, F., Drewe, P., Kahles, A., Jean, G., Vilhjálmsson, B., Jagoda, J., Irez, S., Voronin, V., Song, Q., Long, Q., Rätsch, G., Stegle, O., Clark, R.M., and Nordborg, M.*** *(2015). DNA methylation in Arabidopsis has a genetic basis and shows evidence of local adaptation. eLife* ***4,*** *e05255.*

***Horvath, R., Laenen, B., Takuno, S., and Slotte, T.*** *(2019). Single-cell expression noise and gene-body methylation in Arabidopsis thaliana. Heredity* ***123,*** *81-91.*

***Ji, L., Jordan, W.T., Shi, X., Hu, L., He, C., and Schmitz, R.J.*** *(2018). TET-mediated epimutagenesis of the Arabidopsis thaliana methylome. Nature Communications* ***9,*** *895.*

***Ji, L., Mathioni, S.M., Johnson, S., Tucker, D., Bewick, A.J., Do Kim, K., Daron, J., Slotkin, R.K., Jackson, S.A., Parrott, W.A., Meyers, B.C., and Schmitz, R.J.*** *(2019). Genome-Wide Reinforcement of DNA Methylation Occurs during Somatic Embryogenesis in Soybean. The Plant Cell* ***31,*** *2315-2331.*

***Liu, W., Gallego-Bartolomé, J., Zhou, Y., Zhong, Z., Wang, M., Wongpalee, S.P., Gardiner, J., Feng, S., Kuo, P.H., and Jacobsen, S.E.*** *(2021). Ectopic targeting of CG DNA methylation in Arabidopsis with the bacterial SssI methyltransferase. Nature Communications* ***12,*** *3130.*

***Muyle, A., Ross-Ibarra, J., Seymour, D.K., and Gaut, B.S.*** *(2021). Gene body methylation is under selection in Arabidopsis thaliana. Genetics* ***218,*** *iyab061.*

***Srikant, T., Yuan, W., Berendzen, K.W., Contreras-Garrido, A., Drost, H.-G., Schwab, R., and Weigel, D.*** *(2022). Canalization of genome-wide transcriptional activity in Arabidopsis thaliana accessions by MET1-dependent CG methylation. Genome Biology* ***23,*** *263.*

***Takuno, S., Seymour, D.K., and Gaut, B.S.*** *(2017). The Evolutionary Dynamics of Orthologs That Shift in Gene Body Methylation between Arabidopsis Species. Molecular Biology and Evolution* ***34,*** *1479-1491.*

***Zhang, Y., Jang, H., Xiao, R., Kakoulidou, I., Piecyk, R.S., Johannes, F., and Schmitz, R.J.*** *(2021). Heterochromatin is a quantitative trait associated with spontaneous epiallele formation. Nature Communications* ***12,*** *6958.*

***Zilberman, D.*** *(2017). An evolutionary case for functional gene body methylation in plants and animals. Genome Biology* ***18,*** *87.*

2. The authors compared the phenotypes of different epigenetic mutants and implied that loss of CG and not CHG/CHH methylation potentiates transcription of defense-related genes. If this is the case, the vim mutants would be expected to show a similar effect as the met1 mutant.

*Reply: We thank for the reviewer’s valuable suggestion. To test defense priming phenotype of vim mutant (presumably vim1/2/3 triple knockout mutants), we first checked the ABRC and found that only vim single mutants are available in the ABRC, which show virtually no DNA methylation change* *compared to wild-type plants (Figure 1; Stroud et al, 2013, Cell, 152:352-364).*

***Figure 1.*** *Heatmaps of DNA methylation levels of vim single knockout and vim1/2/3 plants CG (Stroud et al, 2013, Cell, 152:352)*

*We thus asked several scientists, who previously reported the triple vim1/2/3 mutants that showed clear DNA methylation changes (Kim et al. Mol Plant. 2014, 7(9): 1470–1485 and Shook et al. Epigenetics, 2014, 9(7):980-986), for the seeds of these mutants. Dr. Eric Richards (Shook et al. Epigenetics, 2014, 9(7):980-986) said that he could send the vim1/2/3 mutant seeds (see Figure 2 the email communication below). Because of the delays in paper works and quarantine examination, however, we were not able to receive the vim1/2/3 mutant seeds from Dr. Eric Richards by this time (December, 23^rd^ 2022).*

***Figure 2.*** *Communication with Dr. Eric J Richards for vim1/2/3 triple mutant request.*

*Nonetheless, based on the previous reports, it appears to be a logical conclusion that the vim1/2/3 mutant is expected to show a similar effect as the met1 mutant, as the reviewer commented. VIM proteins are UHRF1 homologs that recognize mC and recruit MET1 to methylate cytosines. Therefore, as the reviewer may be aware of, CG and nonCG DNA methylation patterns in met1 and* vim1/2/3 mutants are nearly identical (Figures 3 and 4; Feng et al, 2010, PNAS, 107(19):8689-8694; Stroud et al, 2013, Cell, 152:352-364), and their gene expression patterns are almost the same as indicated by the Pearson’s correlation coefficient = 0.984 (Figure 5; Shook et al., 2014, Epigenetics, 9(7):980-986).

***Figure 3.*** *Comparison of CG methylation levels between wild-type and vim1/2/3 mutant lines from Arabidopsis (Feng et al, 2010, PNAS, 107(19):8689-8694)*

***Figure 4.*** *Heatmaps of DNA methylation levels in wild-type, met1, and vim1/2/3 plants (Stroud et al, 2013, Cell, 152:352)*

***Figure 5.*** *VIMs and MET1 regulate extensively overlapping sets of transcripts (Shook et al., 2014, Epigenetics, 9(7):980-986)*

*Moreover, the DNA methylation and gene expression regulated by VIM proteins are previously demonstrated to be mediated almost exclusively through MET1 activity (Stroud et al, 2013, Cell, 152:352-364; Shook et al., 2014, Epigenetics, 9(7):980-986). All these above previous results, in particular, the almost identical gene expression patterns between vim1/2/3 and met1 mutants suggest that defense priming phenotypes are expected to be the same in these two mutants.*

*Because we failed to receive vim1/2/3 seeds from Dr. Richards, we instead compared both the DNA methylation and mRNA expression levels of our defense-related genes (GBM^ddm1^) in met1, vim1/2/3, and Col-0 plants using the aforementioned previous data (Stroud et al, 2013, Cell, 152:352-364; Shook et al., 2014, Epigenetics, 9(7):980-986) to examine whether mCG loss in vim1/2/3 affects the expression of the defense-related genes associated with gene body methylation as in met1.*

*DNA methylation of GBM^ddm1^ genes in met1 and vim1/2/3 showed very strong correlation pattern (Pearson’s correlation coefficient R=0.999), indicating that DNA methylation of GBM^ddm1^ genes in met1 and vim1/2/3 are very similar to each other (Figure 6).*

***Figure 6.*** *DNA methylation of GBM^ddm1^ in met1 and vim1/2/3. Dots represent gene body CG DNA methylation difference of GBM^ddm1^ between met1 and Col-0 (x-axis), vim1/2/3 and Col-0 (y-axis). DNA methylation data were retrieved from* *previously published data (Stroud et al, 2013, Cell, 152:352-364)*

*Consistent with these observations, the previous study also reported that met1 and vim1/2/3 showed very strong correlation in gene expression (Pearson’s correlation coefficient R = 0.984, Shook et al., 2014, Epigenetics, 9(7):980-986). Gene expression levels of GBM^ddm1^ genes in met1 and vim1/2/3 showed a similar expression pattern, however, less correlation coefficient (Figure 7, Pearson’s correlation coefficient R=0.690).*

***Figure 7.*** *Gene expression of GBM^ddm1^ in met1 and vim1/2/3. Dots represent gene expression fold changes of GBM^ddm1^ between met1 and Col-0 (x-axis), vim1/2/3 and Col-0 (y-axis). Gene expression data were retrieved from previously published data (Shook et al., 2014, Epigenetics, 9(7):980-986)*

*Although gene expression correlation coefficient of GBM^ddm1^ genes in met1 and vim1/2/3 decreased (R = 0.984 for total genes to R=0.690 for GBM^ddm1^ genes), DNA methylation and gene expression patterns of GBM^ddm1^ genes in met1 and vim1/2/3 suggest that, as reviewer mentioned, vim1/2/3 would be expected to show a similar effect as the met1 mutant.*

3. The Conclusion is not precise. Page 9 Lines 47-50, “Overall, both TSS TE-LM…….”; Page 13, Lines 14-16, “We identified……” ; based on these descriptions, not only GBM potentiates transcription of defense-related genes as concluded in Conclusion.

*Reply: We thank for the reviewer’s comment. We agree with the reviewer that how we became to focus on GBM^ddm1^ is not clear among TSS TEM-like^ddm1^, gbTEM-like^ddm1^, and GBM^ddm1^ all of which were found to augment transcription. Thus, we clarified this point in* ***Results*** *as follows:*

**“GBM^ddm1^ is associated with defense response (page 12, top)**

We next examined whether genes with TSS TEM-like^ddm1^, gbTEM-like^ddm1^ or GBM^ddm1^ under BAsubDC were associated with defense response. To this end, we performed the enrichment analysis for these genes and found that ‘defense response to bacterium’ genes were significantly enriched in genes with GBM^ddm1^ (Figure 5E). Unlike genes with GBM^ddm1^, however, genes with TSS TEM-like^ddm1^ or gbTEM-like^ddm1^ had no enriched GO terms because a majority of them are pseudogenes or transposons with no GO terms annotated. Based on these results, we focused on the genes with GBM^ddm1^ hereafter…”

We deleted the following previous expression on page 13, lines 14-16:

“We identified an association between gbTE-LM^ddm1^ or GBM^ddm1^ level and transcriptional activation during the defense response. Both gbTE-LM^ddm1^ and GBM^ddm1^ genes were hyperactivated during a weakly primed defense response, but only GBM^ddm1^ genes were enriched in defense-related genes.”

Correspondingly, to clarify this point, we also added the underlined sentence to the previous **conclusion** on page 21, as follows:

“Of note, DDM1-mediated TE-like methylation located in the transcription start site and gene body also augmented transcription during defense priming, but there is no significant enrichment of defense-related genes among them. Overall, we hypothesize that DDM1-mediated GBM modulates the inducibility of the immune response, functioning as a fundamental mechanism for faster and stronger defense induction upon pathogen challenge.”

4. Figure 2D/F, explain how the tests were performed in Methods.

*Reply: For the statistical testing in Figure 2D/F, we performed Fisher’s exact test using the following online software: Easy Fisher Exact Test Calculator (2018, November 13) at* [*https://www.socscistatistics.com/tests/fisher/default2.aspx*](https://www.socscistatistics.com/tests/fisher/default2.aspx)*. We added the details in the “Statistical testing” section in the Methods.*

5. Figure 7, BAsubDC condition was used for other experiments except this one, explain why “BADC” was used here instead of “BAsubDC”.

*Reply: We thank for the reviewer’s comment. When we tried to select defense priming-related mutants (e.g., ddm1), we intentionally used BAsubDC, a sub-optimal condition for BA treatment (10 ml of 30 μg/ml BABA), which does not induce defense priming in Col-0 but in ddm1. Under this condition,* *whether the mutants of the DNA methylation factors suppress bacterial growth can be effectively evaluated.*

*However, to test the priming phenotypes of the priming regulator candidate knockouts in Col-0 background, we used a higher dose of BABA (10 ml of 35 μg /ml BABA; optimal BABA-induced priming condition) than the above suboptimal dose. Under this optimal priming condition, defense priming was induced in Col. Thus, if priming regulator candidates play a role in the regulation of defense priming, their mutants would have compromised defense priming phenotype during the pathogen challenge, and this phenotype change can be effectively evaluated in this optimal priming condition. We examined defense phenotypes in mutants of priming regulator candidates under mockDC and the optimal BADC conditions and found that mutations of GPK1, BRL3, KIN11, AT1G17230 exhibited compromised defense priming phenotype (Revised Figure 7A).*

*In summary, to clearly evaluate the difference in bacterial growth for selection of ddm1 and testing priming phenotypes of the priming regulator candidates, we used suboptimal and optimal concentrations of BABA, respectively, and this is why the results for BAsubDC are not included for Figure 7A. To clarify this point, we revised the corresponding descriptions in* ***Results*** *as follows (see underlined below):*

***“Loss of CG methylation enables an augmented defense response in weakly primed plants (page 4)***

*… High-dose BABA treatment (10 ml of 35 μg/ml) for 3 days can prime an immune response in Col-0 plants (Zimmerli et al., 2000; Ton et al., 2005; Conrath et al., 2006) (Figure S1A and S1B). However, lowering the dose (10 ml of 30 μg/ml BABA) and shortening the treatment time (2 days), named ‘sub-optimal’ (BAsub), did not enhance resistance in Col-0 plants (Figure 1A and Figure S1C); therefore, whether the DNA methylation mutants suppress bacterial growth can be effectively evaluated in BAsub…”*

***“A gene with DDM1-mediated GBM contributes to defense response priming. (page 15)***

*… To test the function of priming regulator candidates through their mutants in Col-0 background, we used a higher dose of BABA (10 ml of 35 μg/ml BABA) than that used for weak priming in the BAsub condition, which fully primes the defense response in Col-0 (Figure S1A and S1B; BA). Under such condition, whether the mutants of the candidates compromise BABA-induced priming can be effectively evaluated…”*

***Revised Figure 7 (A)*** *Bacterial growth in the mutants of five hyperactivated network hub genes in ddm1 during defense response with optimal priming. We included npr1-1 as a positive control. Experiments were performed as described in the legend of Figure 1, except that a higher concentration of BABA (BA; 10 ml of 35 μg/ml per plant) was applied three days before DC inoculation to fully prime plants. p-values by Student’s t-test. n.s.; not significant (p>0.05). Error bars indicate SEM (n ≧ 5). SAR, systemic acquired resistance. cfu, colony forming units.*

6. Figure 8B/8C/8E, use capitals for the first word in y axis title.

*Reply: As suggested, we used capitals for the first word in y axis title as shown below.*

***Revised Figure 8B/8C/8E***

7. Figure 8F, add dots (or other symbols) to represent the value of each Arabidopsis accession.

*Reply: As suggested, we added dots to represent the value of each Arabidopsis accession in the revised Figure 8F. For GBM variants, the individual Arabidopsis accessions are indicated in different colors. For demethylated variants, the individual Arabidopsis accessions are indicated with different dot shapes. Of note, the Arabidopsis accessions with the non-synonymous mutation in demethylated variants were further distinguished in yellow. The same schemes were used in Figure S11D.*

***Revised Figure 8 (F)*** *Relative expression of novel priming regulators in natural variants. (Top) The average GBM level of GPK1 and BRL3 in GBM variants and demethylated variants. (Bottom) Expression of GPK1 or BRL3 under mockDC condition was normalized to 1. Six natural variants and Col-0 were grouped by their GBM level at GPK1 and BRL3. Col-0, Kondara, and UK-1 maintained GBM at both genes, and Ann-1, Bik-1, Kas-1, and*

*Kro-0 lost GBM at GPK1 and BRL3. RT-qPCR data from three replicates per natural variant were averaged*

*(n=9 for GBM variants, n=12 for demethylated variants).* *Yellow color indicates demethylated variants with non-synonymous mutation in each gene (Bik-1, Kro-0 in GPK1; Ann-1, Kas-1 in BRL3). Error bars indicate standard deviation. p-values by Students t-test.*

**Reviewer #3**

In plants, gene body methylation (GBM) is known to be highly variable both at the inter- and intra-species level, and is positively associated with gene expression at many loci. In this study, Lee et al. demonstrate that GBM loci can also be responsible for potentiating transcriptional changes when methylation levels are reduced. By testing ddm-1 and Col-0 plants for responses to pathogen inoculation upon priming, the authors find that a subset of differentially regulated genes (ddm1 vs Col-0) in primed plants carry hypomethylated GBM and function in defense response. Hypomethylated GBM loci in ddm-1 also exhibit variable chromatin marks. Closer inspection of a few priming regulator genes that are upregulated in ddm1 mutants (upon pathogen inoculation after priming) reveals that the association between gene body hypomethylation and increased gene expression is consistent with patterns observed in natural variants.

The authors show that several genes are differentially expressed between ddm-1 and Col-0 when both plants are primed with BABA and infected with DC3000, but that methylation differences do not vary during all treatments (mock, BAsub, mockDC, BAsubDC). It is proposed that this may be due to a transcription potentiation effect. While gene expression changes (ddm1 vs Col-0) have been analysed under all four treatments, a similar quantitative analysis of differential methylation and differential chromatin properties in the same plants (and for the same treatments) is missing. Such an analysis would provide a clearer understanding of how priming influences epigenetic and epigenomic marks to differentially regulate genes during pathogen inoculation.

From the results, it is clear that GBMddm1 loci are distinct from other GBM loci in their expression levels and chromatin properties - yet, only a small proportion (<5%) of GBMddm1 loci overlap with all DEGs (ddm1/Col-0) in all treatment conditions (as shown in Supplementary Dataset 1). Therefore, a majority of differential expression changes in the BAsubDC condition are still not accounted for by DDM-1 mediated gene body methylation. It may also be interesting to examine GBM at genes that are differentially expressed in BAsubDC but are not differentially expressed in mockDC condition.

Overall, the manuscript is well written and presented. While the authors have carried out extensive analyses to examine hypomethylated GBM loci in ddm-1 mutants, their results are largely restricted to examining differences in methylation between ddm-1 and Col-0 in standard conditions, of which only a few loci GBMddm1 loci are associated with transcriptional changes upon pathogen inoculation. It is interesting that differentially expressed (upregulated) genes exhibiting hypomethylated GBM (GBMddm1) are associated with defense response, but it is unclear whether this is due to the direct effect of DDM-1 mediated GBM or an indirect effect involving both methylation changes and changes in chromatin properties. Therefore, despite the results being well explained, the findings do not provide sufficient advancement in our current understanding of adaptive epigenetic regulatory mechanisms.

*Reply: As requested by the reviewer, we performed H1 and H2A.Z profiling using ChIP-seq and then analyzed differential the chromatin properties (see our point-by-point response below for details).*

Minor comments:

*    The authors split their analysis of GBM by comparing loci that have lost methylation in ddm-1 (GBMddm1) with all other GBM loci (GBMIND), which have unchanged methylation in Col-0. However, Figure 3D shows that GBMIND loci also include some genes that gain methylation in ddm-1. Therefore, it would be more informative if hypomethylated loci, hypermethylated loci and loci with unchanged methylation would be distinguished in all downstream analyses.

*Reply: We thank for this suggestion. After observing ddm1 decreases CG methylation in Figure 3C (see below), we think that the next question should be whether the CG hypomethylation is driven by ddm1 or not. Given the question, it would be more logical to split the genes depending on whether their CG methylation levels are decreased (hypomethylated) or not (unchanged and hypermethylated). When we compared CG methylation levels between Col-0 and ddm1 (mock condition), the distribution of the loci* *(see Figure 3C below) clearly indicates that there are two major groups of loci, and it thus seems that it would be appropriate to focus on the analysis of these two groups, rather than artificially splitting the loci into additional groups. We previously defined these two groups as ‘GB-LM^ddm1^’ (ddm1-dependent) and ‘GB-LM^IND^’ (ddm1-independent), respectively (we now changed GB-LM to GBM-like in response to Reviewer 1’s suggestion). However, as the reviewer pointed out, the GB-LM^IND^ (now GBM-like^weak^ in the revised manuscript) group also includes hypomethylated loci (e.g., the loci below the dotted line representing the 10% decrease; see the GBM-like^weak^ regions in Figure 3C) and even seemingly hypermethylated loci, which makes the definition of ‘ddm1-independent’ (IND) misleading. Thus, we redefined the two groups of the loci as ‘loci strongly hypomethylated in ddm1 (GBM-like^ddm1^)’ and ‘loci with relatively weak or no changes in ddm1 (GBM-like^weak^)’ in the text, and abbreviated them as ‘ddm1’ and ‘weak’, respectively.*

***Revised Figure 3*** ***(B)*** *Violin plots of CG methylation levels of GBM-like in Col-0 and ddm1 plants.* ***(C)*** *Scatter plot of CG methylation level of GBM-like regions in Col-0 (x-axis) and ddm1 (y-axis) plants. Each dot represents a gene body methylation locus. Orange dots indicates GBM-like^ddm1^, and blue dots indicate GBM-like^weak^. GBM-like^ddm1^; strongly demethylated GBM-like regions in ddm1. GBM-like^weak^; GBM-like regions that their methylation levels are weakly affected or not affected in ddm1.*

*    The authors could be more clear in indicating which genotypes were used for calculating methylation levels, and which genotypes were compared for identifying differentially expressed genes (both in the main text and figures). For example, it is understood that GBMddm1 methylation levels are estimated based on the ddm-1 mutant, but are methylation levels at GBMIND loci based on ddm-1 or Col-0?

*Reply: We apologize for this misleading definition of ‘GB-LM^ddm1^’ and ‘GB-LM^IND^’, which might have caused this confusion. As described above, we changed ‘GB-LM^ddm1^’ and ‘GB-LM^IND^’ as ‘GBM-like^ddm1^’ and ‘GBM-like^weak^’, respectively. In Figure 3C, we determined the ‘GBM-like^ddm1^’ and ‘GBM-like^weak^’ loci by comparing CG methylation levels between Col-0 and ddm1 (mock condition), and the same genotypes (ddm1 vs. Col-0) were thus used for ‘GBM-like^ddm1^’ and ‘GBM-like^weak^’.*

*On the other hand, when we identified differentially expressed genes (DEGs) in Figure 2, a set of DEGs were identified by comparing gene expression levels between ddm1 and Col-0 in each of the four treatment conditions (mock, BAsub, mockDC, and BAsubDC). When we combined all DEGs identified from the four conditions, the total number of DEGs was 11138 DEGs as shown in revised Figure S2A (see below). Among these 11138 DEGs, we selected the DEGs (3125 hyperactivated and 3061 suppressed in ddm1) that were changed in BAsub, mockDC, or BAsubDC, but not changed in mock condition (see ‘No’ flow at ‘Expression changes in mock condition’ in revised Figure S2A below).* *Of note, we excluded the genes (basal up and down defined by ‘Yes’ at ‘Expression changes in mock condition’ in revised Figure S2A below) changed in mock condition from our analysis because they would not be associated with priming or defense response. We presented these DEGs (3125 hyperactivated and 3061 suppressed in ddm1) in revised Figure 2A (see below).*

*Moreover, for the analysis in revised Figure 5B (see below), in each of the four treatment conditions, we first identified a set of condition-dependent GBM-like^ddm1^ loci (e.g., revised Figure 3C displays GBM-like^ddm1^ loci in mock condition), and then divided the condition-dependent GBM-like^ddm1^ loci into pGBM-like^ddm1^, TSS GBM-like, and GBM^ddm1^ depending on whether each GBM-like^ddm1^ locus belongs to the promoter, TSS, and gene body, respectively, as illustrated in revised Figure S5A (see below). We next identified the condition-dependent DEGs in the corresponding condition, and examined log_2_-fold-change distributions of the DEGs that overlapped with genes having GBM^ddm1^ loci in the corresponding condition. For example, when we made the boxplots for mockDC condition (see the box in revised Figure 5B below), we identified the GBM^ddm1^ loci and DEGs by comparing DNA methylation and gene expression data generated from ddm1 and Col-0 plants in the mockDC condition, selected a subset of the DEGs that overlapped with genes having GBM^ddm1^ loci under mockDC condition, and then displayed the log2-fold-change distribution of the selected subset of DEGs. For the case of ‘all genes’ (now ‘all DEGs’; control for GBM^ddm1^), we showed the log2-fold-change distribution of all DEGs identified from mockDC. Therefore, our GBM^ddm1^ under mockDC indicate genes with demethylated GBM in ddm1 plants (compared to Col-0) under mockDC condition. To clarify this point, we revised the descriptions in* ***Results*** *as follows:*

***“DDM1-mediated TEM-like at gene bodies and transcriptional start sites potentiates transcription. (page 9, bottom to page 10, top)***

*… Next, we analyzed the effects of the three gene groups on gene expression. To this end, we first selected all DEGs between ddm1 and Col-0 plants in each treatment condition (e.g., BAsubDC). From the three gene groups, we then selected the DEGs with demethylated pTEM-like (pTEM-like^ddm1^), TSS TEM-like (TSS TEM-like^ddm1^), and gbTEM-like (gbTEM-like^ddm1^) in ddm1 (vs. Col-0) under the treatment condition (e.g., BAsubDC). We next compared the expression changes of all DEGs and DEGs with each of the three TEM-like^ddm1^ (Figure 4B, 4C, 4D and Supplemental Data 1) in ddm1 (vs. Col-0) under the treatment condition (e.g., BAsubDC) …”*

***Revised Figure S2 (A)*** *Categorization of differentially expressed genes in ddm1 (vs. Col-0) during weakly primed defense response.*

***Revised Figure 2 (A)*** *Heatmaps of gene expression in Col-0 and ddm1 plants before (0 dpi; mock and BAsub) and after (1 dpi; mockDC and BAsubDC) DC inoculation. For Col-0 and ddm1 columns, log_2_ fold change (FC) against Col-0 mock samples were plotted. For ddm1/Col-0 columns, log_2_ fold change in ddm1 plants against Col-0 under four different conditions were plotted.*

***Revised Figure S5 (A)*** *Number of Araport11 gene annotations with gene body-like methylation (GBM-like). Promoter GBM-like; GBM-like located at 0 to 1.5 kb upstream of genes, TSS GBM-like; GBM-like overlap with TSS, gene body GBM-like (GBM); GBM-like overlap with gene body but do not overlap with TSS, conventionally GBM. TTS, transcription termination sites.*

***Revised Figure 5 (B)*** *Boxplots of gene expression of DEGs with GBM-like strongly demethylated in ddm1 (GBM-like^ddm1^) at gene body (GBM^ddm11^). Expression fold change (log_2_ (ddm1/Col-0)) of all* *DEGs at each condition was plotted as control. DEG, differentially expressed gene. Of note, red box was added for response to reviewer. It is removed from Revised Figure 5(B).*

*    Supplementary dataset 1 and Figure 2C,2E can include number of DEGs identified in ddm-1 hyper, ddm-1 sup, ETI hyper, ETI sup and the overlaps between these DEGs with basal ddm-1 DEGs (all DEGs). The intersections between GBMddm1, GBMIND loci with ddm-1 hyper and ddm-1 sup DEGs is also not indicated in the text or supplement.

*Reply: We thank for the suggestion. We included all the numbers requested by the reviewer in Figure 2C, E, Figure 2E-F or Supplementary Data 1: 1) numbers of ddm1 hyper, ddm1 sup, ETI hyper, ETI sup in revised Figure 2C, E (see numbers in parenthesis); 2) the overlaps of the DEGs with ddm1 basal up and ddm1 basal down in Supplementary data 1 ( see ‘Figure2’ worksheets), and 3) the overlaps of GBM^ddm1^ and GBM^weak^ with ddm1 hyper or ddm1 sup DEGs in Supplementary data 1 (see ‘Figure5B-C’ worksheets).*

***Revised Figure 2C-2F***

Comments for author on the revised manuscript:

The authors have made substantial efforts to revise the manuscript using data from previously published datasets, and also generate further experimental results by examining natural variants and additional mutant lines. While this is notable, some questions from the first round of review are still not sufficiently addressed:

1) Whether DDM1-mediated GBM directly regulates transcription, or chromatin marks may be involved (Reviewer #2-1 and #2-2):

The authors examined GB-LMddm1 and GB-LMIND genes in h1, ddm1, and h1ddm1 mutants, showing that the reduction in methylation levels at GB-LMddm1 loci are dependent on H1 levels (Figure 3F-H). This demonstrates that GBMddm1 regions are overall enriched for histone variant H1 (Figure 6C). Based on previous studies, H1 is known to have a role in suppressing the expression of genes, which is in contrast to some GB-LMddm1 loci being expressed upon primed pathogen stress, and therefore the authors dismiss the involvement of H1 in the regulation of these genes. However, the role of H1 in regulating these genes is still debatable since it is not clear whether DEGs carrying GBMddm1 showed different H1 levels compared to other GBMddm1 loci, and to what extent H1 levels changed at these loci during the different treatments.

While H2A.W levels appear to show minimal variation among all GB-LM regions (Figure 3E), H3K36me3 levels are lower and H2A.Z levels are higher for GB-LMddm1 genes (Figure S6) compared to GBMIND genes. Next, the authors demonstrated that h2a.w and h2a.z mutants do not show altered response to pathogen stress upon priming. However, these mutants carry depleted H2A.W and H2A.Z not only at GBMddm1 loci, but also throughout the genome. Given that H2A.Z levels are higher for GB-LMddm1 genes, it is still unclear whether the presence of H2A.Z in these loci can also be causal for differential transcription.

The authors also carried out ChIP-qPCR to examine four priming regulator genes, and found that H3K4me3 levels in all genes are higher in ddm-1 mutants than Col-0, and show significant differences for GPK1 (direct regulator) and KIN11 (indirect regulator). No such changes were identified for H3K9me2, although H3K27me3 levels were increased for KIN11.

Together, these findings indicate that GBMddm1 genes are overall marked by higher H1 levels, higher H2A.Z levels, lower H3K36me3 levels compared to GBMIND genes, and altered levels of H3K4me3 in some priming regulators. Further investigation is required into the chromatin marks exhibited by the subset of GBMddm1 genes that are differentially transcribed. Therefore, it is recommended that the authors carry out a more systematic analyses of chromatin properties in:
a) ddm-1 and Col-0 genotypes, under all 4 treatment conditions (mock, mockDC, BAsub, BAsubDC)

*Reply: We sincerely thank the reviewer for this valuable suggestion.* *During the 2^nd^ revision, we performed chromatin immunoprecipitation (ChIP) sequencing of H1 and H2A.Z to investigate the genome-wide H1 and H2A.Z distributions under mock, BAsub, DC, and BAsubDC conditions in Col-0 and ddm1 plants. Of note, this would be the first report that systematically investigated H1 and H2A.Z distribution and DNA methylation levels in wild-type and ddm1 plants, especially during defense priming.*

*The previous study suggested that CG DNA methylation and histone H1 are largely overlapping yet independent (Choi et al. 2020, Mol. Cell, 77, 310–323). Despite the independent relationship in general, some transposons lost H1 and DNA methylation simultaneously. For these transposon loci, the loss of H1 might be associated with the gain of active histone markers (H3K4me3) or active transcription, rather than with the loss of DNA methylation. Consistent with this previous study, we found that H1 levels at TEM-like or GBM-like regions were similar regardless of demethylation levels in ddm1 plants (Revised Figure 3Gand 3H), indicating minimal variations among TEM-like or GBM-like regions, except for some TEM-like that heavily lost DNA methylation and gained DNA accessibility in ddm1 plants (magenta box in Revised Figure 3G). Of note, the genes with highly methylated TEM-like do not overlap with genes with GBM^ddm1^ associated with defense priming. These data suggest that GBM-like, including GBM^ddm1^, is not associated with H2A.Z levels.*

***Revised Figure 3*** ***(G)*** *Scatter plots of CG methylation change at TEM-like regions in relation to histone H1 and H2A.Z change in ddm1. Magenta box indicates highly demethylated TEM-like regions with increased DNA accessibility in ddm1.* ***(H)*** *Scatter plots of CG methylation change at GBM-like regions in relation to histone H1 and H2A.Z change in ddm1.* ***(G and H)*** *Dot color shows log_2_ ratio of DNA accessibility in Col-0 to ddm1.*

*It has long been suggested that H2A.Z and DNA methylation distributions are mutually exclusive in the Arabidopsis genome (Zilberman et al. 2008, Nature, 456(7218):125–129). In the previous study, Zilberman et al. compared the DNA methylation patterns between Col-0 and met1 plants by methylated DNA IP (MeDIP). MeDIP uses an antibody that specifically binds to methylated cytosine to enrich methylated DNA and thus cannot differentiate CG and nonCG cytosine contexts. Therefore, instead of using the MeDIP data, we obtained another previously reported bisulfite sequencing dataset (Stroud et al. 2013, Cell, 152:352-364) generated from Col-0 and met1 and then defined TEM-like and GBM-like regions as described in our manuscript. For H2A.Z profile, we obtained a previously reported H2A.Z ChIP-chip data (tiling array; Zilberman et al. 2008, Nature, 456(7218):125–129) generated from Col-0 and met1. After mapping the defined TEM-like and GBM-like regions to those in H2A.Z ChIP-chip data based on the locus information on the tiling array, we then compared CG methylation and H2A.Z changes between Col-0 and met1 in TEM-like or GBM-like regions (Revised Figure S3K).*

***Revised Figure S3*** ***(K)*** *Scatter plots of CG methylation change at TEM-like and GBM-like regions in relation to H2A.Z changes in met1. Magenta box indicates highly demethylated TEM-like regions with increased histone H2A.Z levels in met1. DNA methylation data and H2A.Z data are from Stroud et al. (Stroud et al, 2013, Cell, 152:352-364) and Zilberman et al. (Zilberman et al. 2008, Nature, 456(7218):125–129).*

*As previously reported, some severely demethylated TEM-like (lost more than 80% of CG methylation in met1 plants) gained H2A.Z in met1 plants. In contrast, H2A.Z levels at the GBM-like regions were not altered in met1 plants with minimal variation among the GBM-like regions. These results suggest that TEM-like, but not GBM-like, suppresses H2A.Z enrichment in Arabidopsis. Consistent with met1 plants, CG demethylation did not affect H2A.Z levels at GBM-like regions in ddm1 plants (Revised Figure 3H above). Of note, demethylated TEM-like did not gain H2A.Z in ddm1 plants either. Because met1 is a DNA methyltransferase, loss of met1 led to complete loss of CG methylation (Revised Figure S3L). Compared to met1, ddm1 plants retained more than an average 20% CG methylation at TEM-like (Revised Figure 3A). Because nonCG methylation levels of met1 and ddm1 are similar, it is possible that the remaining CG methylation at demethylated TEM-like in ddm1 plants suppresses the accumulation of H2A.Z.*

***Revised Figure S3*** ***(L)*** *Violin plots of DNA methylation levels of TEM-like regions in Col-0 and met1 plants. DNA methylation data are from Stroud et al. (Stroud et al, 2013, Cell, 152:352-364)*

***Revised Figure 3*** ***(A)*** *Violin plots of DNA methylation levels of TEM-like regions in Col-0 and ddm1 plants.*

*Next, we systematically compared H1 and H2A.Z levels at TEM-like^ddm1^ under the four treatment conditions (mock, BAsub, mockDC, and BAsubDC). First, when we identified significantly demethylated regions (TEM-like^ddm1^) in ddm1 from each condition, we found that the identified TEM-like^ddm1^ regions tended to show shared demethylation across the conditions (Revised Figure 4E below), although a small portion showing condition-specific demethylation are present (because they could not simply pass the threshold we set to identify TEM-like^ddm1^ regions). This further analysis confirmed the previous findings that DNA methylation patterns in ddm1 are essentially invariable under mock, BAsub, DC, and BAsubDC (Revised Figure 4E, CG, CHG, and CHH). We then examined H1 and H2A.Z level changes at TEM-like^ddm1^ in ddm1 plants (vs. Col-0) under the four conditions. TSS TEM-like^ddm1^, pTEM-like^ddm1^, and gbTEM-like^ddm1^ lost H1 under the four conditions (see blue in the ‘H1’ heat map of Revised Figure 4E and Figure S4H below), whereas those of TEM-like^weak^ maintained H1 (see relatively white in the ‘H1’ heat map of Revised Figure 4E and Figure S4H). We found hyperactivation of genes with TSS TEM-like^ddm1^ and gbTEM-like^ddm1^ under BAsubDC conditions (see red in the ‘expression’ heat map of Revised Figure 4E). Still, their H1 or DNA methylation patterns was constantly low under all four conditions, which had no correlation with the increasing upregulation patterns of gene expression levels over the conditions (i.e., higher activation in BAsubDC). We also found hyperactivation of genes with TSS TEM-like^ddm1^ and gbTEM-like^ddm1^ did not show altered H2A.Z levels under four conditions in ddm1 plants, and the H2A.Z levels were not significantly different between TEM-like^weak^ and TEM-like^ddm1^, suggesting that H2A.Z changes are not associated with those of the expression at TEM-like regions.*

***Revised Figure 4 (E)*** *CG, CHG, CHH methylation, histone H1, H2A.Z, and gene expression profiles of genes with TEM-like^ddm1^ at TSS, gene body, and promoter (TSS TEM-like^ddm1^, gbTEM-like^ddm1^, pTEM-like^ddm1^) and TEM-like^weak^ at TSS, gene body, and promoter (TSS TEM-like^weak^, gbTEM-like^weak^, pTEM-like^weak^) in Col-0 and ddm1 under the four treatment conditions. TEM-like^ddm1^; strongly demethylated TEM-like in ddm1, TEM-like^weak^; TEM-like that their methylation levels are weakly affected or not affected in ddm1.*

***Revised Figure S4*** ***(H)*** *Boxplots of histone H1 (left) and H2A.Z levels (right) of genes with TSS TEM-like^weak^ and TSS TEM-like^ddm1^ in each treatment conditions.*

*Next, we systematically compared H1 and H2A.Z levels at GBM-like^ddm1^ and GBM-like^weak^ under four conditions. Like TEM-like^ddm1^, GBM-like^ddm1^ (GBM^ddm1^ and pGBM-like^ddm1^) tended to show shared demethylation over the conditions (Revised Figure 5D below). When we examined H1 and H2A.Z level changes at GBM-like^ddm1^ in ddm1 plants under four conditions, we could not identify significant changes of H1 or H2A.Z levels in ddm1 plants (vs. Col-0), neither did significant differences between GBM-like^ddm1^ and GBM-like^weak^ correlating with gene expression change (Revised Figure 5D and Figure S5C below, H1 and H2A.Z). Of note, H1 level negatively correlates with active histone markers (e.g. H3K4me3; Choi et al. 2020, Mol. Cell, 77, 310–323). DNA demethylation at transposons often causes massive chromatin remodeling, which could suppress H1 enrichment. However, the DNA methylation loss is less likely to dramatically change the chromatin environment of transcribed genes, as we previously demonstrated (Revised Figure S12B-D below). Therefore, it is plausible that H1 enrichment did not affect GBM-like^ddm1^ regions as much as TEM-like^ddm1^ regions.*

***Revised Figure 5 (D)*** *CG methylation, histone H1, H2A.Z, and gene expression profiles of genes with GBM-like^ddm1^ at gene body and promoter (GBM^ddm1^, pGBM-like^ddm1^) and GBM-like^weak^ at gene body and promoter (GBM^weak^, pGBM-like^weak^) in Col-0 and ddm1 under the four treatment conditions. GBM-like^ddm1^; strongly demethylated GBM-like in ddm1, GBM-like^weak^; GBM-like that their methylation levels are weakly affected or not affected in ddm1.*

***Figure S12 (B-D)*** *Histone H3K4me3* ***(B)****, H3K9me2* ***(C)****, and H3K9me3* ***(D)*** *enrichment around novel priming regulators in mockDC and BAsubDC condition in Col-0 and ddm1 plants. #1-#3 primers amplify TSS-proximal region, gene body region and TTS-proximal region, respectively. Asterisks indicate statistically significant change in enrichment (p<0.05, Col-0 mockDC vs. ddm1 mockDC, Col-0 BAsubDC vs. ddm1 BAsubDC). p-values by Student’s t-test.*

b) GBMddm1 genes that overlap with DEGs (ddm-1 hyper, ddm-1 sup) and genes that do not overlap with DEGs

*Reply: As the reviewer suggested, we compared H1 and H2A.Z properties between DEGs with GBM^ddm1^ (‘ddm1 hyper’ or ‘ddm1 sup’ in Revised Figure S5C) and non-DEGs with GBM^ddm1^ (‘noDEG’ in Revised Figure S5C). There was a significant difference in H1 level change between ‘noDEG’ and ‘ddm1 hyper’ groups under BAsubDC. However, no significant difference between ‘ddm1 hyper’ and ‘ddm1 sup’ groups indicates that such difference appears to be not systematic. Moreover, H1 levels at ‘ddm1 hyper’ groups showed no changes across the conditions compared to Col-0 except for BAsub condition. H2A.Z levels at ‘ddm1 hyper’ and ‘noDEG’ groups changed similarly under four conditions, indicating minimal variations of H2A.Z among the DEG and noDEG groups, which are consistent with the findings from Revised Figure 3H above. Therefore, these results data suggest that H1 and H2A.Z levels did not correlate with transcriptional change.*

***Revised Figure S5 (C)*** *Boxplots of* *histone H1 (left) and H2A.Z (right) levels of GBM^ddm1^ genes (ddm1 vs. Col-0, BAsubDC) overlap with DEGs (ddm1 hyper, ddm1 sup) and GBM^ddm1^ genes that do not overlap with DEGs (noDEG) in each treatment conditions.*

This could also shed light on why there are large transcriptional changes in the BAsubDC vs mock conditions despite methylation levels being largely invariable (comment from Reviewer #1-1).

*Reply: Based on the results we present here and in the revised manuscript, we propose that gene body methylation (GBM) demethylated in ddm1 (GBM^ddm1^) suppresses transcriptional potentiation of defense-related genes rather than directly regulating transcriptional initiation as transcription factors do (Table 1). In pathogen-treated condition (DC), ddm1 showed similar expression of defense-related genes compared to Col-0. However, ddm1 showed the increased expression of these genes after treatment of pathogen together with β-aminobutyric acid, a priming-inducing chemical (BAsub + DC, BAsubDC); Revised Figure 1-2 and S1; see the table 1 below). On the other hand, in met1, expression of defense-related genes was increased in DC compared to Col-0 (Revised Figure 1 and the table below). Comparison of the alteration in DNA methylation and gene expression between ddm1 and met1 in DC and BAsubDC (see the table 1 below) suggests that there is a threshold for induction of defense-related genes in WT upon DC treatment, and DNA hypomethylation lowers the threshold, called “transcriptional potentiation”: a nearly zero threshold for met1, but a non-zero threshold for ddm1, which is lower than that in WT. Accordingly, met1 with the zero threshold induces defense-related genes upon DC treatment, whereas ddm1 with a non-zero threshold requires an additional defense signal, such as BAsub treatment, together with DC treatment to induce defense-related genes.*

*In ddm1 plants, only transposon-like methylation (TEM-like) showed perturbation of H1 (Revised Figure 3G and 3H), suggesting that H1 and H2 variant levels were not associated with transcriptional potentiation of gene body methylated defense-related genes in ddm1 plants (see the table below). Investigation of H3 modification patterns at the putative priming regulators with GBM^ddm1^ showed that H3K4me3, H3K27me3, H3K9me2 levels did not associate with hyperactivation of these genes in ddm1 plants (Revised Figure S12). These results suggest that loss of GBM in ddm1 increased the transcriptional potential of target genes, and a part of these genes could be hyperactivated when there is a signaling cue, in this case, β-aminobutyric acid -induced priming and pathogen challenge (Revised Figure S13).*

***Table 1.*** *Summary table of DNA methylation, gene expression, histone H1, H2A.Z changes in met1, ddm1 and Col-0 under DC, BAsub+DC condition. * indicates anticipated results based on data from met1 mock condition and no significant difference of DNA methylation, histone H1, H2A.Z in ddm1, Col-0 under DC, BAsub+DC condition. TEM, transposon methylation*

***Revised Figure S13.*** *The contribution of gene body CG-hypomethylation to transcriptional potentiation during weakly-primed and fully primed defense response. (A) Demethylation of GBM^ddm1^ increased transcriptional potential of priming regulators, leading to hyperactivation by weak defense priming (BAsubDC). (B) Demethylation of GBM^ddm1^ increased the transcriptional potential of non-priming-target genes, but no transcriptional change occurred due to a lack of upstream signal.*

*Consistent with our results, previous studies showed that only a tiny portion of epigenetically regulated genes (or transposons) were transcriptionally activated. For example, complete loss of nonCG methylation activates only a handful of transposons (Stroud et al., 2014, Nat Struct Mol Biol, 21:64-72), and total loss of gene body methylation affects the expression of hundreds of antisense transcripts among thousands of gene body methylated genes (Choi et al. 2020, Mol. Cell, 77, 310–323). Also, global loss of H1 regulate expression of only a subset of genes (Sun et al., 2021, bioRxiv 2021.07.08.451606; Serna-Pujol et al., 2022, Nucleic Acids Research, 50:3892–3910) Unlike transcription factors that directly regulate gene expression, epigenetic modification sets the environment for transcription. Thus, it is plausible that only a small portion of epigenetically regulated genes (or transposons) were transcriptionally activated.*

2) Whether there is a causal relationship between reduced GBM and transcription (Reviewer #2-3): The authors showed examples of natural variants that lack GBM in a few selected priming regulators, where absence of methylation is associated with increased gene expression. While these findings confirm their proposed model, the authors have not mentioned if they have accounted for
a. genetic polymorphisms: Do the four naturally demethylated variants of GPK1 and BRL3 also show polymorphisms that could contribute to transcriptional differences (in mock conditions)? For example, Shahzad et al. 2021 showed that the association between GBM and expression could be confounded by genetic variation at certain loci, such as FRIGIDA.

*Reply: We thank for the valuable comment. As the reviewer suggested, we checked the polymorphisms of BRL3 and GPK1* *in four naturally demethylated variants of BRL3 and GPK1 [Ann-1, Bik-1, Kas-1, and Kro-0].*

*First, we checked* *basal gene expression levels of BRL3 and GPK1 in Col-0 and naturally demethylated variants. We did not find significant transcriptional differences of BRL3 and GPK1 among Col-0 and naturally demethylated variants in mock condition (Revised Figure S11(F)).*

***Revised Figure S11 (F)*** *Gene expression of BRL3 and GPK1 in Col-0 and four naturally demethylated variants of BRL3 and GPK1 (Ann-1, Bik-1, Kas-1, and Kro-0). Gene expressions were measured in mock condition. UBQ1 expression was used as an internal control. Different letters indicate significant differences at p≦0.05 from one-way ANOVA with Tukey’s correction (α = 0.05). Error bars indicate standard deviation (n = 3).*

*We further investigated the* *polymorphisms of BRL3 and GPK1 in gene-coding region among four naturally demethylated variants (Polymorphism data were retrieved from 1001 Genomes Project* [*https://www.1001genomes.org*](https://www.1001genomes.org)*). We found several non-synonymous mutations leading to amino acid changes in naturally demethylated variants (Revised Figure S11G). Given these polymorphisms among naturally demethylated variants, no transcriptional differences of GPK1 and BRL3 among Col-0 and naturally demethylated variants suggest that these polymorphisms are not likely to affect* *hyperactivation of GPK1 and BRL3 during defense response.*

***Revised Figure S11 (G)*** *Polymorphisms of BRL3 and GPK1 in gene region among four naturally demethylated variants of BRL3 and GPK1 (Ann-1, Bik-1, Kas-1, and Kro-0). Red colors indicate non-synonymous substitution and altered amino acid. Polymorphism data were retrieved from 1001 Genomes Project (https://1001genomes.org/)*

b. Chromatin properties: Do the four demethylated natural variants show variation in chromatin marks at the priming regulator genes?

*Reply: We thank for the valuable suggestion. To answer the reviewer’s question, we assayed the chromatin marks (H3K4me3, H3K9me2, H3K27me3) at the priming regulator genes in four demethylated natural variants (Ann-1, Bik-1, Kas-1, and Kro-0). Except H3K27me3 of AT1G17230 in Bik-1, chromatin marks in four demethylated natural variants showed similar patterns at the priming regulator genes to those of Col-0. Altogether with polymorphism data, these results suggest that neither polymorphism nor chromatin marks are associated with transcriptional potentiation of the priming regulator genes. We included this result to revised manuscript and Revised Figure S12A.*

***Revised Figure S12 (A)*** *Histone H3K4me3, H3K9me2, and H3K27me3 enrichment around novel priming regulators in mockDC and BAsubDC conditions in Col-0 and four natural variants (Ann-1, Bik-1, Kas-1, Kro-0) that both GPK1 and BRL3 were demethylated. #1-3 primers amplify TSS-proximal region, gene body region and TTS-proximal region, respectively. Histone enrichment data of Col-0 from Figure S12 (B, C, and D). Asterisks indicate statistically significant change in enrichment (p<0.05, Col-0 mockDC vs. each natural variant mockDC, Col-0 BAsubDC vs. each natural variant BAsubDC). p-values by Student’s t-test.*

**Third round of review**

**Reviewer 1**

The authors have addressed all my concerns. I don't have other questions.

**Reviewer 3**

The authors have taken notable efforts to perform additional ChIP-seq experiments and analyses, both for samples under the various treatment conditions as well as in natural variants. The revised main text and figures (especially Figures 3-5, and associated supplementary figures) are clear to read and understand. Altogether, these changes have greatly improved the manuscript overall.

While the authors have provided adequate responses to the comments from the previous review, the following minor comments also need to be addressed:

1. Figure 1B and Figure S1D essentially convey the same information, while Figure S1D specifically compares the samples under mockDC and BAsubDC conditions. However, the expression levels seem to differ between both figures (for example, in Figure 1B the expression level of WRKY29 for met1 mutants goes upto 3, while in Figure S1D it is shown at 1). Please correct or clarify this discrepancy in the Figure legend.
2. Figure 2A and Figure S2B: Please indicate the heatmap column header as Col-0/Col-0 mock, ddm1/Col-0 mock, to make it easier for understanding the figures.
3. Page 6- Line 56 : The following sentence is ambiguous and must be made clearer: “On the other hand, among the above plants, BAsub treatment boosted immunity only in met1 plants (Figure 1A), suggesting that CHH demethylation contributed to the constant activation of basal defense.” Do the authors mean ‘CG demethylation’ contributed to increased defense response, or do they mean to indicate that CHH/CHG demethylation in the other mutants did not seem to increase defense response against the pathogen?
4. Page 7, Line 16: Please indicate in the sentence that methylation profiles were investigated for “untreated” Col-0 and ddm1 plants.
5. Figure 4E and Figure 5D: The y-axis scales for log2FC in the boxplots are from -2 to 3, while the heatmap shows only -0.7 to 0.7. Please correct or clarify this discrepancy in the Figure legend.
6. How are DEGs assigned to the TEM-like/GBM-like categories (promoter, TSS, gb) if they overlap with more than one category? For example, if a DEG has both promoter GBM/TEM and TSS GBM/TEM, is it assigned to one or both of the categories? Please clarify in Methods.
7. Page 11, Line 14 : “Like TEM-like, the GBM-like levels were reduced …”. To be more explicit, are the authors referring to methylation levels?
8. Page 13, Line 19 : “We then investigated the chromatin environment at 10,332 genes with CG methylation at gene body.” Please clarify that you are referring to GBM genes in the Col-0 background. Similarly in Figure 6(C-F) and Figure S6(A-D), it is suggested that the authors indicate in the y-axis label that the chromatin levels were measured based on the Col-0 background.
9. Page 15, Line 41 : Do the authors mean “BADC” and not “BA”?
10. Figure S11D: ‘GBM variants’ should be written as ‘GBM variants in GPK, BRL3’ and similarly, ‘demethylated variants’ as ‘demethylated variants in GPK,BRL3’. Otherwise it is confusing to see that the demethylated variants have higher DNA methylation compared to GBM variants for AT1G17230 and KIN11.
11. Figure S12: The colour scheme can be improved to better distinguish the different samples (the shades of grey are difficult to interpret).
12. Page 19, Lines 13- 18: “H3 histone modification patterns around the priming regulators (GPK1, BRL3, AT1G17230, and KIN11) showed no significant correlation with gene expression changes in ddm1 (Figure S12B-D).” Firstly, the authors have not shown any results of a correlation made between histone modifications and gene expression in these genes. Secondly, as they state in Page 18 (Lines 20-28) and show in Figure S12(B-D), H3K4me3 and H3K27me3 are present in significantly high levels in ddm1 mutants, and have a tendency to further increase during the BAsubDC condition. While these are indeed repressive marks and therefore not conventionally expected to activate gene expression, their high levels in these hub-genes is still a notable observation. Therefore, it is suggested that the authors acknowledge in the Discussion that there are alternate possibilities for these marks to indirectly affect transcription through other pathways that remain to be explored.
13. Page 21, Line 40 : “We also discover that knockout of hypomethylated gene in ddm1 impair BABA-induced resistance.” This sentence is ambiguous, and could be changed to “We also discover that knocking out candidate genes (known to be hypomethylated in ddm1 mutants) in Col-0 background can impair BABA-induced resistance.”

If the above comments are addressed, I support the publication of this manuscript.

Apart from the minor comments listed above, there are a few general comments on the Abstract and Conclusion sections:
DNA methylation does not always occur only at transposons and gene bodies (also in promoters and intergenic regions), and demethylation of transposons does not always result in gene activation for disease resistance. The sentences that refer to this on Page 1 (Line 27 -31) must therefore be corrected to a more generalized statement indicating that the above could be one of the many possible roles for DNA methylation marks in the genome.
Regarding the mention of GPK1, a more general remark may be to state that knockout of candidate genes that are hypomethylated in ddm1 can impair response to primed pathogen infection.
It could also be emphasized that the results from this study uncover GBMddm1 loci which are distinct from conventional GBM loci (such as in having variable chromatin properties).

**Authors Response**

**Point-by-point responses to the reviewers’ comments:**

Reviewer #1: The authors have addressed all my concerns. I don't have other questions.

*Reply: Thank you for the reviewer’s valuable comments, which were crucial for the improvement of this manuscript.*

Reviewer #3: The authors have taken notable efforts to perform additional ChIP-seq experiments and analyses, both for samples under the various treatment conditions as well as in natural variants. The revised main text and figures (especially Figures 3-5, and associated supplementary figures) are clear to read and understand. Altogether, these changes have greatly improved the manuscript overall.  

While the authors have provided adequate responses to the comments from the previous review, the following minor comments also need to be addressed:

1.    Figure 1B and Figure S1D essentially convey the same information, while Figure S1D specifically compares the samples under mockDC and BAsubDC conditions. However, the expression levels seem to differ between both figures (for example, in Figure 1B the expression level of WRKY29 for met1 mutants goes upto 3, while in Figure S1D it is shown at 1). Please correct or clarify this discrepancy in the Figure legend.

*Reply: The reviewer is correct in that the same data are presented after different normalization. In Figure 1B, we normalized the expression levels of WRKY genes with respect to those in Col-0 under the mock condition [i.e., log_2_(height of black bars in the mock condition) = 0]. In Figure S1D, to compare the expression difference of each gene between mockDC and BAsubDC, we normalized the expression levels of WRKY genes with respect to those in Col-0 under mockDC [i.e., log_2_(height of white bars in Col-0) = 0]. This comparison might be not straightforward from the data in Figure 1B with those from the other conditions, and we thus added Figure S1D to clarify the comparison.*

*To clarify the difference in the normalization, as the reviewer suggested, we added the following descriptions to the revised Figure 1B and Figure S1D legends (see the highlighted below).*

***Figure 1B:***

***(B)*** *Expression of WRKY genes in Col-0, ddm1, and met1 plants before (0 dpi; mock and BAsub) and after (1 dpi; mockDC and BAsubDC) DC inoculation (n ≧ 3). p-values by Student’s t-test. Expression levels for each gene were normalized by that of an internal control UBQ1, which were further normalized with respect to those in Col-0 under the mock condition. Log_2_(normalized expression levels) are presented.*

***Figure S1D:***

***(D)*** *Expression of WRKY genes in Col-0, ddm1, and met1 plants one day post Pseudomonas syringae pv. tomato DC3000 inoculation with (BAsubDC) or without (mockDC) sub-optimal concentration of BABA treatment. Error bars indicate SEM (n ≧ 3). p-values by Student’s t-test. UBQ1 expression was used as an internal control.* *Expression levels for each gene were normalized by that of an internal control UBQ1, which were further normalized with respect to those in Col-0 under mockDC. Log_2_(normalized expression levels) are presented.*

2.    Figure 2A and Figure S2B: Please indicate the heatmap column header as Col-0/Col-0 mock, ddm1/Col-0 mock, to make it easier for understanding the figures.

*Reply: We thank reviewer for suggestion. We included “Col-0/Col-0 mock” and “ddm1/Col-0 mock” on heatmap column header in the revised Figure 2A and Figure S2B.*

3.    Page 6- Line 56 : The following sentence is ambiguous and must be made clearer: “On the other hand, among the above plants, BAsub treatment boosted immunity only in met1 plants (Figure 1A), suggesting that CHH demethylation contributed to the constant activation of basal defense.” Do the authors mean ‘CG demethylation’ contributed to increased defense response, or do they mean to indicate that CHH/CHG demethylation in the other mutants did not seem to increase defense response against the pathogen?

*Reply: Comparison of the resistance between mutants and their corresponding wild-type plants under mockDC condition revealed that both CG demethylation (met1) or CHH demethylation (met1, drm1 drm2, ddc, and ddcc) enhanced resistance to DC, whereas CHG demethylation (cmt3) has little effect on resistance to DC (Figure 1A). However, comparison of the resistance between mockDC and BAsubDC showed that BAsub treatment boosted resistance to DC only in mutants showing CG demethylation (met1 and ddm1). These results suggest that global loss of CG methylation in met1 and ddm1 plants contributed to the enhanced disease resistance with weak BAsub priming, whereas loss of CHH methylation enhanced resistance to DC independent of priming.*

*Accordingly, we revised the paragraph as follows (see the highlighted below):*

*Moreover,* *met1, drm1 drm2, ddc, and ddcc plants showed enhanced resistance to DC without BAsub (Figure 1A). Comparison of the resistance between mutants and their corresponding wild-type plants under mockDC condition revealed that both CG demethylation (met1) or CHH demethylation (met1, drm1 drm2, ddc, and ddcc) enhanced resistance to DC, whereas CHG demethylation (cmt3) has little effect on resistance to DC (Figure 1A). Consistent with resistance in met1 plants, up- and* *downregulated genes in met1 plants under the mock condition [51] significantly overlapped with ETI hyper and ETI sup genes (Figure 2C–2F). However, comparison of the resistance between mockDC and BAsubDC showed that BAsub treatment boosted resistance to DC only in mutants showing CG demethylation (met1 and ddm1), consistent with significant overlaps of ETI-responsive genes with ddm1 hyper or sup genes in weakly primed conditions. These results suggest that global loss of CG methylation in met1 and ddm1 plants contributed to the enhanced disease resistance with weak BAsub priming, whereas loss of CHH methylation enhanced resistance to DC independent of priming.*

4.    Page 7, Line 16: Please indicate in the sentence that methylation profiles were investigated for “untreated” Col-0 and ddm1 plants.

*Reply: We thank reviewer for suggestion. We revised the sentence as “We first produced genome-wide DNA methylation profiles in Col-0 and ddm1 plants with no treatment of BAsub or DC.*

5.    Figure 4E and Figure 5D: The y-axis scales for log2FC in the boxplots are from -2 to 3, while the heatmap shows only -0.7 to 0.7. Please correct or clarify this discrepancy in the Figure legend.

*Reply: We previously used -0.7 to 0.7 to focus on the major body (Q1 to Q3) of the data, but this color bar scale tended to exaggerate the expression difference of the presented data. As the reviewer suggested, we thus changed the color bar scales of the heatmaps (-2 to 2 for Figure 4E and -1 to 1 for Figure 5D) to present the differences of expression levels considering the data outside of Q1 and Q3 presented by the whisker bars in the boxplots. Of note, -2 and 2 for Figure 4E included 97.0% of the data presented by the boxplots for 8 loci groups, and -1 to 1 for Figure 5D included 93.0% of the data.*

6.    How are DEGs assigned to the TEM-like/GBM-like categories (promoter, TSS, gb) if they overlap with more than one category? For example, if a DEG has both promoter GBM/TEM and TSS GBM/TEM, is it assigned to one or both of the categories? Please clarify in Methods.

*Reply: We removed all the genes that overlap with more than one category of DNA methylated region. To clarify this, we added the descriptions in Results and Methods as follows:*

***Results:***

***(page 9) DDM1-mediated TE-like methylation at gene bodies and transcription start sites potentiates transcription.***

*TEs can exist in the promoter and gene body to affect expression of nearby genes. Thus, we next investigated whether TEM-like^ddm1^ is associated with gene expression in a weakly primed defense response. To precisely evaluate the unique effect of DDM1-mediated TEM-like (TEM-like^ddm1^) at promoter, transcription start site (TSS), or gene body to gene expression, we selected the following three mutually exclusive groups of genes (Figure S4A): 1) 4,702 genes with TEM-like only at the promoter (pTEM-like), 2) 1,250 genes with TEM-like only at TSS (TSS TEM-like), 3) 1,187 genes with TEM-like only at gene body (gbTEM-like).*

***(page 11) DDM1-mediated GBM potentiates transcription.***

*We next investigated the effect of GBM-like^ddm1^ on gene expression. GBM-like can also occur in the promoter and TSS to affect expression of target genes. Similar to genes with TEM-like^ddm1^, we thus selected three mutually exclusive groups of genes (Figure S5A): 1) 2,157 genes only with pGBM-like, 2) 83 genes only with TSS GBM-like, and 3) 6,816 genes only with gbGBM-like (i.e., GBM).*

***(page 26) Methods****:*

***Categorization of genes with TEM-like and GBM-like loci***

*We first selected genes with TEM-like DNA methylation at the promoter, TSS, or gene body. To evaluate the unique effect of TEM-like DNA methylation at the promoter, TSS, and gene bodies on gene expression, we then removed genes with TEM-like DNA methylation at multiple regions (e.g., both promoter and gene body), resulting in pTEM-like, TSS TEM-like, and gbTEM-like (Figure 4). The same selection procedure was used for genes with GBM-like DNA methylation (Figure 5). Of note, if a gene had both TEM-like and GBM-like DNA methylation, this gene was excluded from our analysis.*

*7.    Page 11, Line 14 : “Like TEM-like, the GBM-like levels were reduced …”. To be more explicit, are the authors referring to methylation levels?*

*Reply: We thank reviewer for clarification. As the reviewer suggested, we revised the sentence as “Like TEM-like, CG methylation levels of the GBM-like were reduced in ddm1 plants and showed similar patterns over gene structure across the treatment conditions.”*

8.    Page 13, Line 19 : “We then investigated the chromatin environment at 10,332 genes with CG methylation at gene body.” Please clarify that you are referring to GBM genes in the Col-0 background. Similarly in Figure 6(C-F) and Figure S6(A-D), it is suggested that the authors indicate in the y-axis label that the chromatin levels were measured based on the Col-0 background.

*Reply: As the reviewer suggested, we revise following sentence as “We then investigated the chromatin environment at 10,332 genes with CG methylation at gene body in Col-0.”*

*We also included Col-0 background in y-axis label in Figure 6(C-F) and Figure S6(A-D)*

9.    Page 15, Line 41 : Do the authors mean “BADC” and not “BA”?

*Reply: We previously used “BA” to indicate higher dose of β-aminobutyric acid (BABA) (10 ml of 35 μg/ml BABA) than that used for weak priming in the “BAsub” (10 ml of 30 μg/ml BABA) condition. For clarity, we changed “BA” to “BAopt” in the text and figures as well. Thus, the corresponding sentence was revised as “To test the function of priming regulator candidates through their mutants in Col-0 background, we used a higher dose of BABA (10 ml of 35 μg/ml BABA; BAopt) than that used for weak priming in the BAsub condition, which fully primes the defense response in Col-0 (BAoptDC in Figure S1A; BAopt, BAoptDC in Figure S1B).”*

10.    Figure S11D: ‘GBM variants’ should be written as ‘GBM variants in GPK, BRL3’ and similarly, ‘demethylated variants’ as ‘demethylated variants in GPK, BRL3’. Otherwise it is confusing to see that the demethylated variants have higher DNA methylation compared to GBM variants for AT1G17230 and KIN11.

*Reply: We thank reviewer for suggestion. We revised Figure S11D legend as follows (see the highlighted below):*

***(D)*** *Relative expression of novel priming regulators in natural variants. (Left) The average GBM level of AT1G17230 and KIN11 in GBM variants in GPK1 and BRL3 and demethylated variants in GPK1 and BRL3. (Right) Expression of AT1G17230 and KIN11 under mockDC condition was normalized to 1. Six natural variants and Col-0 were grouped by their GBM level at GPK1 and BRL3. Col-0, Kondara, and UK-1 maintained GBM at both genes, and Ann-1, Bik-1, Kas-1, Kro-0 lost GBM at GPK1 and BRL3. All natural variants maintained GBM at AT1G17230 and KIN11. RT-qPCR data from three replicates per natural variants were averaged (n=9 for GBM variants, n=12 for demethylated variants). Error bars indicate standard deviation.*

11.    Figure S12: The colour scheme can be improved to better distinguish the different samples (the shades of grey are difficult to interpret).

*Reply: We thank reviewer for this suggestion. As the reviewer suggested, we changed the color scheme of Figure S12 for better interpretation.*

12.    Page 19, Lines 13- 18: “H3 histone modification patterns around the priming regulators (GPK1, BRL3, AT1G17230, and KIN11) showed no significant correlation with gene expression changes in ddm1 (Figure S12B-D).” Firstly, the authors have not shown any results of a correlation made between histone modifications and gene expression in these genes. Secondly, as they state in Page 18 (Lines 20-28) and show in Figure S12(B-D), H3K4me3 and H3K27me3 are present in significantly high levels in ddm1 mutants, and have a tendency to further increase during the BAsubDC condition. While these are indeed repressive marks and therefore not conventionally expected to activate gene expression, their high levels in these hub-genes is still a notable observation. Therefore, it is suggested that the authors acknowledge in the Discussion that there are alternate possibilities for these marks to indirectly affect transcription through other pathways that remain to be explored.

*Reply: We thank reviewer for valuable suggestion. We changed “correlation” to “association” and also added the alternate possibility that the reviewer pointed out as follows (see the highlighted below):*

*“H3 histone modification patterns around the priming regulators (GPK1, BRL3, AT1G17230, and KIN11) showed no apparent association with gene expression changes in ddm1 (Figure S12B-D). On the other hand, interestingly, the levels of the repressive marks H3K9me2 and H3K27me3 were increased in ddm1 mutants and tended to increase further under BAsubDC condition, suggesting alternate possibilities that these marks could indirectly affect transcription through pathways that remain to be explored.”*

13.    Page 21, Line 40 : “We also discover that knockout of hypomethylated gene in ddm1 impair BABA-induced resistance.” This sentence is ambiguous, and could be changed to “We also discover that knocking out candidate genes (known to be hypomethylated in ddm1 mutants) in Col-0 background can impair BABA-induced resistance.”

*Reply: We thank reviewer for suggestion. We revised the sentence as follow*

*“We also discover that knockout of candidate genes, which were hypomethylated in ddm1 mutants, in Col-0 background can impair BABA-induced resistance.”*
